# Supplementary material for: Multi-omic phenotyping reveals host-microbe responses to bariatric surgery, glycaemic control and obesity
Source: Commun Med (Lond). 2022 Oct 7;2:127. doi: 10.1038/s43856-022-00185-6 (PMC9546886; doi:10.1038/s43856-022-00185-6)
Supplement: Supplementary file 1 — Supplementary Information [file 43856_2022_185_MOESM1_ESM.pdf]

Supplementary Information

Table of Contents

Demographics **.......... 3**

Sample Demographics 3

Supplementary Table 1 – Demographics of all samples 3

Supplementary Table 2 – Demographics of microbiome analysis samples 5

Collected Samples 6

Supplementary Figure 1 – Collected samples 6

Dietary advice and surgical technique 7

Supplementary Note 1 – Dietary advice and surgical technique 7

Metabolic Analysis **.......... 8**

Example ^{1}H-NMR spectra 8

Supplementary Figure 2 – ^{1}H-NMR untargeted analysis 8

Diabetes – ^{1}H-NMR untargeted analysis 9

Supplementary Figure 3 – OPLS-DA models comparing ^{1}H-NMR analysis of participants with type 2 diabetes Vs participants without diabetes in serum, urine and faecal biofluids 9

Supplementary Table 3 – Serum metabolites measured by ^{1}H-NMR spectroscopy discriminating between participants with and without type 2 diabetes 10

Supplementary Table 4 – Urinary metabolites measured by ^{1}H-NMR spectroscopy discriminating between participants with and without type 2 diabetes 11

Supplementary Table 5 – Faecal metabolites measured by ^{1}H-NMR spectroscopy discriminating between participants with and without type 2 diabetes 11

Supplementary Data 2 – Metabolites measured with ^{1}H-NMR spectroscopy in faecal, urine and serum samples in participants with type 2 diabetes (T2D), impaired glucose tolerance (IGT) and participants without diabetes 12

Diabetes – Targeted analyses 13

Supplementary Data 3 – Bile acids in faecal and serum samples in participants with type 2 diabetes (T2D), impaired glucose tolerance (IGT) and participants without diabetes 13

Supplementary Data 4 – SCFA and other carboxylic acids in faecal, urine and serum samples in participants with type 2 diabetes (T2D), impaired glucose tolerance (IGT) and participants without diabetes 14

Supplementary Data 5 - Serum metabolites in participants with type 2 diabetes (T2D), impaired glucose tolerance (IGT) and participants without diabetes 15

Bariatric Surgery – ^{1}H-NMR untargeted analysis 16

Supplementary Figure 4 – Longitudinal RM-MCCV-PLSDA model comparing ^{1}H-NMR analysis of participants before and after bariatric surgery in serum 16

Supplementary Figure 5 – Serum ^{1}H-NMR RM-MCCV-PLSDA subgroup analysis 17

Supplementary Table 6 – Serum metabolites discriminating between participants pre- and post-surgery 18

Supplementary Figure 6 – Longitudinal RM-MCCV-PLSDA model comparing ^{1}H-NMR analysis of participants before and after bariatric surgery in urine 19

Supplementary Figure 7 – Urine ^{1}H-NMR RM-MCCV-PLSDA subgroup analysis 20
Supplementary Table 7 – Urinary metabolites discriminating between participants pre- and post-surgery 22
Supplementary Figure 8 – Longitudinal RM-MCCV-PLSDA model comparing ^{1}H-NMR analysis of participants before and after bariatric surgery in faeces 22
Supplementary Figure 9 – Faecal ^{1}H-NMR RM-MCCV-PLSDA subgroup analysis 23
Supplementary Table 8 – Faecal metabolites between participants pre- and post-surgery....... 24
Supplementary Data 6 – Change in metabolites measured with ^{1}H-NMR spectroscopy in faecal, urine and serum samples post bariatric surgery 25
Bariatric Surgery – Targeted analyses....... 26
Supplementary Data 7 – Change in bile acids post bariatric surgery in faecal and serum samples....... 26
Supplementary Data 8 – Change in SCFA and other carboxylic acids post bariatric surgery in faecal, urine and serum samples 27
Supplementary Data 9 – Changes in serum metabolites post bariatric surgery 28

Gut Microbiome Analysis....... 29

Supplementary Figure 10 – PCoA comparing gut microbiota analysis in participants with T2D Vs participants without diabetes....... 29
Supplementary Figure 11 – PCoA comparing gut microbiota analysis in participants with T2D, impaired glucose tolerance and participants without diabetes....... 30
Supplementary Figure 12 – Phylogenetic tree comparing gut microbiota of participants with T2D to participants without diabetes....... 31
Supplementary Figure 13 – PcoA comparing gut microbiota analysis of participants pre and post RYGB 32
Supplementary Figure 14 – Phylogenetic tree comparing gut microbiota of participants pre and post RYGB 33
Supplementary Figure 15 - Phylogenetic tree comparing gut microbiota KEGG pathways in participants pre and post RYGB 34
Supplementary Figure 16 – PcoA comparing gut microbiota analysis of participants pre and post VSG 35
Supplementary Figure 17 – Phylogenetic tree comparing gut microbiota of participants pre and post VSG 36

Integrative Analysis....... 37

Supplementary Figure 18 – DIABLO model comparing integrated datasets of participants with and without T2D 37
Supplementary Figure 19 – DIABLO model comparing changes in integrated datasets after RYGB Vs VSG....... 38
Supplementary Figure 20 – Metabolite – Microbiome correlations 39

Demographics

Sample Demographics

Supplementary Table 1 – Demographics of all samples

Pre-Op	
n	156
Sex, n (M:F)	48:108
Age (Years)	46±11.5
Weight (Kg)	129.1±27.6
BMI (kg/m^2)	46.0±8.1
T2D, n (%)	66(42.3)
IGT, n (%)	26(16.7)
HbA1c (mmols/mol)	48.7±16.5
Metformin, n (%)	60(38.4)
Insulin, n (%)	19(12.2)

Pre-Op Subgroups	T2D	Non-diabetic
n	66	64
Sex, n (M:F)	29:37	9:55
Age (Years)	51.15±10.09	40.47±11.66*
Weight (Kg)	126.9±26.79	127±19.76
BMI (kg/m^2)	45.46±7.46	46.02±7.41
Metformin, n (%)	57(86.4)	0(0)
Insulin, n (%)	17(25.8)	0(0)
HbA1c (mmols/mol)	62.33±16.52	35.71±7.41*
Duration of T2D (years)	7.72±7.28	

** $p<0.05$ subgroup comparison*

Operative Subgroups	RYGB Pre-Op	RYGB Post Op	VSG Pre-Op	VSG Post Op
n	23		26	
Sex, n (M:F)	2:21		7:19	
Age (Years)	47.22±10.15		42.5±9.79	
Weight (Kg)	121.3±19.85	96.65±15.45‡	128.1±25.47	106.37±24.41‡
BMI (kg/m^2)	45.6±6.22	36.34±4.87‡	46.45±7.87	38.58±7.87‡
T2D, n (%)	11(47.8)		8(30.1)	
IGT, n (%)	2(8.7)		4(15.4)	
Metformin, n (%)	10(43.4)	5(21.7)	6(23.1)	4(15.4)
Insulin, n (%)	6(26.0)	3(13.0)	1(3.8)	0(0)
HbA1c (mmols/mol)	50.74±17.78	39.0±9.42‡	45.04±13.17	37.77±5.57‡
Duration of T2D (years)	7.59±7.47		3.87±3.27	

All listed data are means ± standard deviation unless stated otherwise.

No significant difference between operation demographics at baseline.

‡ = $p < 0.05$ pre and post-op comparison

Supplementary Table 2 – Demographics of microbiome analysis samples

Pre-Op Subgroups	T2D	Non-diabetic
n	42	27
Sex, n (M:F)	23:19	4:23
Age (Years)	51.67±9.98	41.56±12.14*
Weight (Kg)	133.7±27.40	129.1±19.82
BMI (kg/m^2)	47.19±8.20	47.15±6.89
Metformin, n (%)	37(88.1)	0(0)
Insulin, n (%)	12(28.6)	0(0)
HbA1c (mmols/mol)	64.71±17.77	36.3±3.24*

** p<0.05 subgroup comparison*

	RYGB		VSG	
Operative Subgroups	Pre-Op	Post Op	Pre-Op	Post Op
n	13		14	
Sex, n (M:F)	2:11		5:9	
Age (Years)	50.15±8.05		44.14±9.52	
Weight (Kg)	121.8±19.43	94.92±14.95‡	133±23.09	110.67±20.05‡
BMI (kg/m^2)	46.55±7.73	36.31±5.36‡	47.76±8.60	39.74±7.43‡
T2D, n (%)	7(53.8)		4(28.6)	
IGT, n (%)	2(15.3)		2(14.2)	
Metformin, n (%)	7(53.8)	5(38.5)	4(28.6)	4(28.6)
Insulin, n (%)	3(23.1)	3(23.1)	1(7.1)	0(0)
HbA1c (mmols/mol)	52±15.96	39.08±6.51‡	48.64±16.61	38.07±6.78‡

All listed data are means ± standard deviation unless stated otherwise.

No significant difference between operation demographics at baseline.

‡ = $p < 0.05$ pre and post-op comparison

Collected Samples

Supplementary Figure 1 – Collected samples

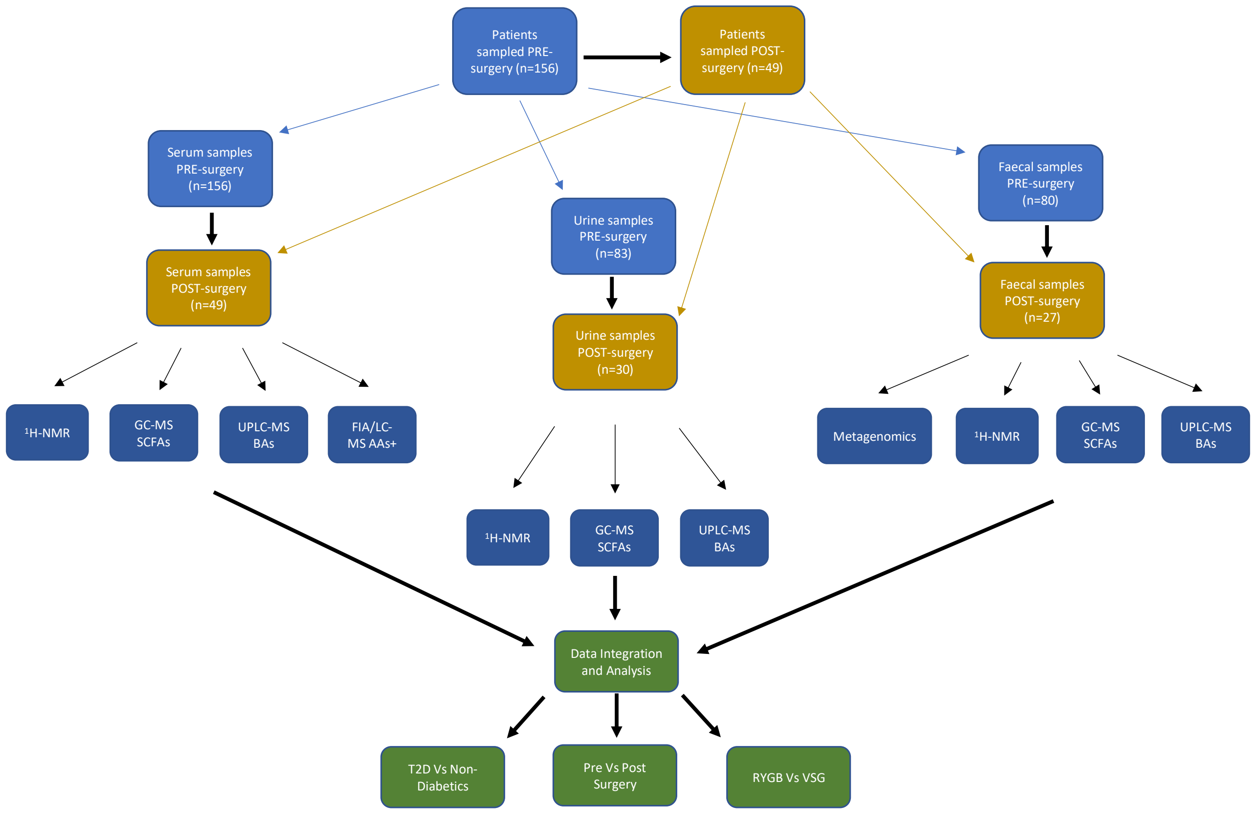

Supplementary Figure 1 – Collected samples pre- and 3-months post-surgery and analyses performed.

SCFAs: Short-chain fatty acid and other carboxylic acids assay. BAs: Bile acid assay. AAs+: Amino acid, biogenic amine, acylcarnitine, phosphatidylcholine and sphingolipid assay.

Dietary advice and surgical technique

Supplementary Note 1 – Dietary advice and surgical technique

Prior to recruitment all patients had attended and complied with a Tier 3 intense supervised multidisciplinary diet and lifestyle program for a minimum of 6 months.

Two weeks prior to surgery participants were placed on a 'liver shrinking' diet which consisted of 100g carbohydrate per day, low fat intake and limiting overall intake to 800-1000 kcal / day. Participants were given an extensive meal plan to help achieve this.

The procedures were performed by 5 consultant surgeons from within the same department. A single dose of 1.2g intravenous co-amoxiclav was given during induction of anaesthesia (clindamycin if penicillin allergic). All procedures were standardised and performed in the same manner as described in brief below.

Roux-en-Y Gastric Bypass (RYGB):

Gastric Pouch - 15 to 30cc gastric pouch created using laparoscopic staplers. Sized using a 34Fr Ewald tube.

Biliopancreatic Limb: 75cm.

Roux Limb: 100cm.

Gastrojejunostomy - Side to side anastomosis using 30mm stapler, enterotomy closed with continuous 3-0 vicryl followed by a second layer of interrupted 3-0 vicryl.

Underwater leak test used to check anastomosis.

Jejunal-Jejunal anastomosis - Side to side using 45mm stapler, enterotomy closed with continuous 3-0 vicryl.

Mesenteric and Petersen's defects were closed with IFA bond.

Vertical Sleeve Gastrectomy (VSG):

Gastric Sleeve formed over a 34Fr orogastric tube.

Stapling commenced 3cm proximal from pylorus.

4-5 firings of 45/60mm staplers with Seamguard staple line re-enforcement.

Underwater leak test used to check staple line.

Post operatively patients were given advice on the texture of foods to consume. This consisted of a liquid diet for 10 days, puree diet for 3-4 weeks, followed by a soft diet for approximately 6 weeks before resuming a normal texture diet. Patients were advised by the dietetics team to follow a healthy balanced diet with sufficient quantities of protein and consume multivitamin & mineral supplements lifelong.

Metabolic Analysis

Example ^{1}H-NMR spectra

Supplementary Figure 2 – ^{1}H-NMR untargeted analysis

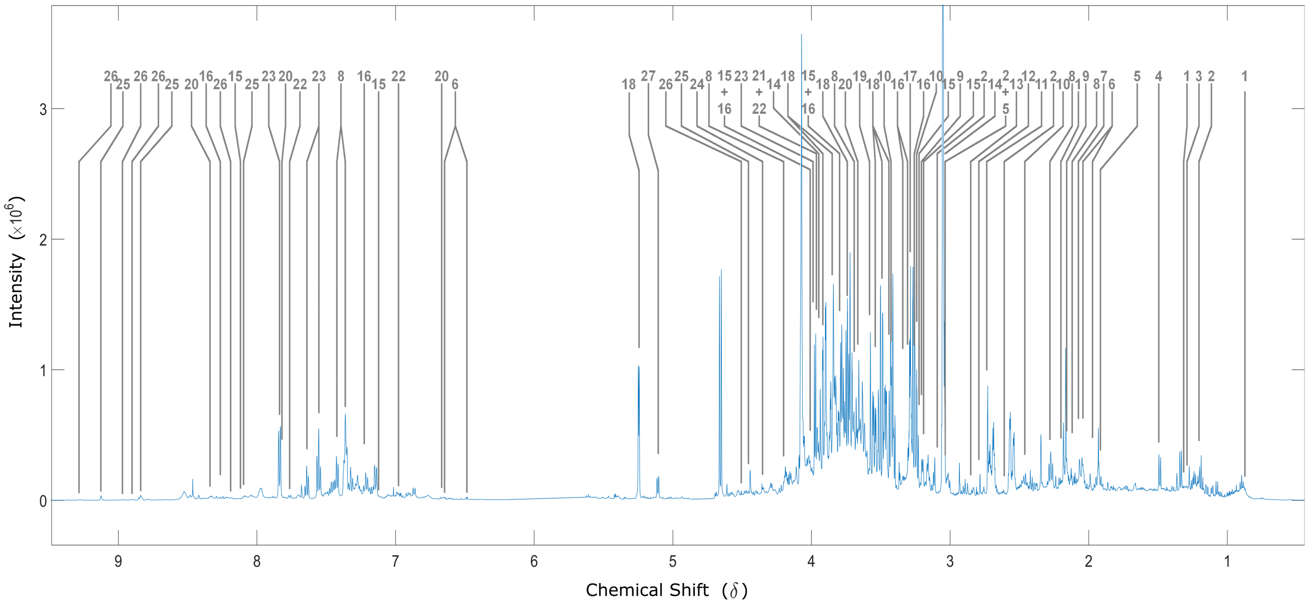

Supplementary Figure 2 – Mean urinary ^{1}H-NMR spectra. Examples of labelled metabolites are shown. Significant metabolite signals from each model are detailed in the tables below.

Numbers: 1, fatty acids (C5–C10); 2, 3-aminoisobutyrate; 3, rhamnitol; 4, alanine; 5, lysine; 6; N-acetyl-S-(1Z)-propenyl-cysteine-sulfoxide; 7, N-acetylneuraminate; 8, phenylacetylglutamine; 9, O-acetylcarnitine; 10, carnitine; 11, dimethylamine; 12, N-acetyl-S-methyl-cysteine-sulfoxide; 13, S-methyl-cysteine-sulfoxide; 14, creatine; 15, 1-methylhistine; 16, 3-methylhistidine; 17, trimethylamine-N-oxide; 18, glucose; 19, glycine; 20, N-methyl-2-pyridine-5-carboxamide; 21, glycolate; 22, 4-hydroxyhippurate; 23, hippurate; 24, tartrate; 25, N-methylnicotinate; 26, N-methylnicotinamide; 27, Acetaminophen glucuronide.

Diabetes – ^{1}H-NMR untargeted analysis

Supplementary Figure 3 – OPLS-DA models comparing ^{1}H-NMR analysis of participants with type 2 diabetes Vs participants without diabetes in serum, urine and faecal biofluids

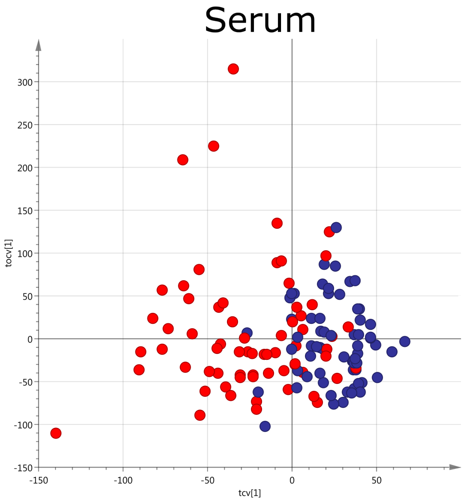

No Diabetes [ ] Vs T2 Diabetes [ ]

OPLS-DA CV Plots

	Serum		Faeces		Urine	
	n	R^2Y, Q^2Y	n	R^2Y, Q^2Y	n	R^2Y, Q^2Y
**T2D Vs No:**	130 (66:64)	0.61, 0.44	68 (42:27) (Par)	0.59, 0.35	68 (41:27)	0.80, 0.12
**T2D Vs No/IGT:**	156 (66:90)	0.55, 0.38	79 (42:38) (Par)	0.60, 0.41	80 (41:39)	0.81, 0.26

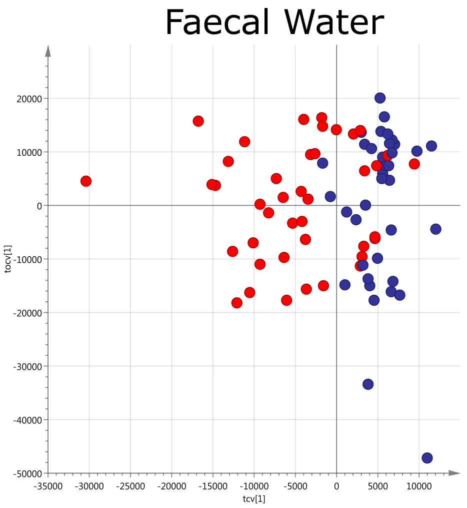

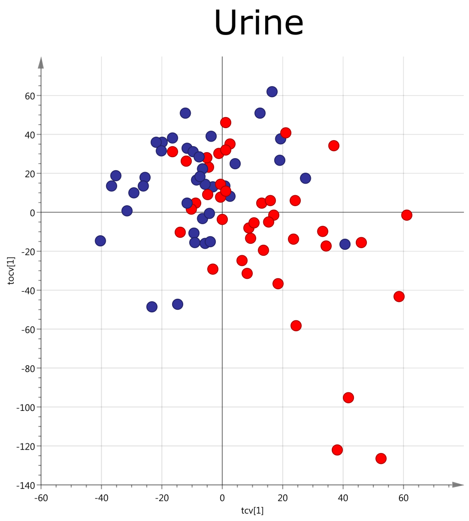

Supplementary Figure 3 – OPLS-DA scores plots of ^{1}H-NMR analysis comparing participants with type 2 diabetes Vs participants without diabetes in serum urine and faecal water samples. Colour coding (blue = non-diabetic, red = T2D) and model scores are shown.

Serum – Type 2 Diabetes			
**Metabolite**	**^{1}H chemical shift δ (multiplicity^a)**	**Association**	**Sig (UV^b)**
VLDL/LDL	0.89 (t), 1.29 (m), 1.59 (m)	↑	Yes (LDL)
Isoleucine	0.96 (t), 1.03 (d), 1.48 (m), 1.26 (m)*, 1.98 (m)*, 3.68 (d)*	↑	Yes
Leucine	0.98 (t), 1.71 (m), 3.73 (t)*	↑	Yes
Valine	1.01 (d), 1.06 (d), 2.28 (m), 3.63 (d)	↑	Yes
Lactate	1.34 (d), 4.13 (q)	↑	Yes
Alanine	1.49 (d), 3.79 (q)*	↑	Yes
Proline	2.01 (m)*, 2.07 (m)*, 2.37 (m), 3.36 (m), 3.43 (m)*, 4.13 (dd)	↑	NA
Pyruvate	2.39 (s) Storm – lactate	↑	Yes
Tyrosine	3.06 (Abx), 3.16 (Abx)*, 3.94 (Abx)*, 6.92 (d), 7.21 (d)	↑	No
α Glucose	5.25 (d) + 3.23 (dd), 3.40 (m), 3.46 (m), 3.52 (dd), 3.73 (m), 3.82 (m), 3.88 (dd)	↑	Yes
HDL	0.86 (m)	↓	Yes
Glutamine	2.15 (m), 2.47 (m), 3.77 (t)*	↓	No
Glycerophosphocholine	3.23 (s broad)	↓	Yes
Phosphocholine / choline	3.24 (s broad)	↓	Yes
Histidine	3.99 (dd), 7.08 (s), 7.80 (s)	↓	Yes

Supplementary Table 3 – Serum metabolites measured by ^{1}H-NMR spectroscopy discriminating between participants with and without type 2 diabetes

Table shows serum metabolites higher or lower in the OPLS-DA model of participants with type 2 diabetes versus participants without diabetes. ^{a}Multiplicity key is as follows: s=singlet, d=doublet, t=triplet, q=quartet, dd=doublet of doublets, m=multiplet. ^{1}H shifts marked by an asterisk (*) were not confirmed experimentally due to the absence of unambiguous resonance values and are taken from the Human Metabolome Database (HMDB). ^{b}Sig (UV) = metabolite significant on univariate analysis of quantified levels.

Urine – Type 2 Diabetes			
**Metabolite**	**^{1}H chemical shift δ (multiplicity^a)**	**Association**	**Sig (UV^b)**
α Glucose	5.25 (d) + 3.23 (dd), 3.40 (m), 3.46 (m), 3.52 (dd), 3.73 (m), 3.82 (m), 3.88 (dd)	↑	Yes
β Glucose	4.66 (d) + 3.23 (dd), 3.40 (m), 3.46 (m), 3.52 (dd), 3.73 (m), 3.82 (m), 3.88 (dd)	↑	Yes
Isobutyrate	1.11 (d), 3.02 (m)*	↓	Yes
Glycine	3.57 (s)	↓	Yes
Creatine	3.04 (s), 3.94 (s)	↓	Yes
Creatinine	3.05 (s), 4.06 (s)	↓	Yes
O-Acetylcarnitine	3.20 (s) (STORM carnitine 3.23 (s))	↓	NA
N-methyl-2-pyridone-5-carboxamide (2PY)	3.64 d)*, 6.67 (d), 7.83 (dd)*, 8.33 (d)	↓	NA
Methylnicotinamide	4.48 (s), 8.19 (t)*, 8.90 (d), 8.96 (d), 9.28 (s)	↓	Yes
Formate	8.46 (s)	↓	Yes

Supplementary Table 4 – Urinary metabolites measured by ^{1}H-NMR spectroscopy discriminating between participants with and without type 2 diabetes

Table shows urinary metabolites higher or lower in the OPLS-DA model of participants with type 2 diabetes versus participants without diabetes. ^{a}Multiplicity key is as follows: s=singlet, d=doublet, t=triplet, q=quartet, dd=doublet of doublets, m=multiplet. ^{1}H shifts marked by an asterisk (*) were not confirmed experimentally due to the absence of unambiguous resonance values and are taken from the Human Metabolome Database (HMDB). ^{b}Sig (UV) = metabolite significant on univariate analysis of quantified levels.

Faeces – Type 2 Diabetes			
**Metabolite**	**^{1}H chemical shift δ (multiplicity)**	**Association**	**Sig (UV^b)**
Dimethylbiguanide (Metformin)	3.05 (s)	↑	Yes
Glycine	3.57 (s)	↑	Yes
Lactate	1.33 (d), 4.12 (q)	↓	Yes
Uracil	5.81 (d), 7.54 (d)	↓	Yes
Leucine	0.96 (t), 1.68 (m), 3.71 (t)*	↓	Yes
Valine	0.99 (d), 1.04 (d), 2.28 (m), 3.62 (d)	↓	Yes
Isoleucine	0.94 (t), 1.01 (d), 1.48 (m), 1.26 (m)*, 1.96 (m)*, 3.66 (d)*	↓	Yes
Tyrosine	3.06 (Abx), 3.16 (Abx)*, 3.94 (Abx)*, 6.91 (d), 7.20 (d)	↓	Yes

Supplementary Table 5 – Faecal metabolites measured by ^{1}H-NMR spectroscopy discriminating between participants with and without type 2 diabetes

Table shows faecal metabolites higher or lower in the OPLS-DA model of participants with type 2 diabetes versus participants without diabetes. ^{a}Multiplicity key is as follows: s=singlet, d=doublet, t=triplet, q=quartet, dd=doublet of doublets, m=multiplet. ^{1}H shifts marked by an asterisk (*) were not confirmed experimentally due to the absence of unambiguous resonance values and are taken from the Human Metabolome Database (HMDB). ^{b}Sig (UV) = metabolite significant on univariate analysis of quantified levels.

Supplementary Data 2 – Metabolites measured with ^{1}H-NMR spectroscopy in faecal, urine and serum samples in participants with type 2 diabetes (T2D), impaired glucose tolerance (IGT) and participants without diabetes

		No (n=64)		IGT (n=26)		T2D (n=66)		Kruskal Wallis	Mann Whitney U Test			Mann Whitney U Test (BH pFDR)		
	Metabolite (selected ppm)	Mean RI (SD)	Median RI (IQR)	Mean RI (SD)	Median RI (IQR)	Mean RI (SD)	Median RI (IQR)	No Vs IGT Vs T2D	No Vs T2D	No Vs IGT	IGT VS T2D	No Vs T2D	No Vs IGT	IGT VS T2D
Serum	HDL CH3 (0.86)	405502 (75376)	399072 (103539)	373269 (72026)	347389 (86510)	339552 (75480)	321616 (92845)	0.000	0.000	0.042	0.040	0.000	0.127	0.127
	VLDL CH3 (0.89)	599622 (226594)	535416 (310022)	660750 (345125)	555730 (264357)	721713 (457679)	591753 (365575)	0.463	0.219	0.674	0.602	0.426	0.840	0.786
	Leucine (0.98)	150295 (23585)	151487 (28424)	172833 (43823)	169755 (57116)	178102 (35881)	169397 (55404)	0.000	0.000	0.020	0.400	0.000	0.071	0.576
	Isoleucine (1.03)	57224 (12050)	56279 (15250)	66751 (22893)	62488 (33904)	71059 (16995)	66999 (23914)	0.000	0.000	0.090	0.238	0.000	0.216	0.429
	Valine (1.06)	166829 (31508)	163255 (37340)	188900 (49535)	186490 (51335)	195276 (40530)	193763 (60066)	0.000	0.000	0.041	0.410	0.000	0.127	0.579
	3Hydroxybutyrate (1.19)	1446798 (782443)	1659305 (786552)	1296772 (789532)	1482672 (1231533)	1369799 (673184)	1373757 (739739)	0.515	0.272	0.471	0.833	0.460	0.647	0.889
	CH2 Lipid (1.30)	1566959 (778744)	1411666 (929480)	1862527 (1219745)	1456914 (1022799)	2161324 (1583595)	1780350 (1230901)	0.046	0.013	0.383	0.310	0.053	0.563	0.497
	CH2CH2COOC Lipid (1.59)	118702 (61232)	105641 (80303)	146254 (100249)	112342 (66708)	166967 (120156)	130788 (105189)	0.027	0.008	0.270	0.327	0.032	0.460	0.508
	Alanine (1.50)	255677 (49227)	258379 (66025)	260016 (48009)	259260 (78271)	310116 (73744)	302883 (90868)	0.000	0.000	0.687	0.002	0.000	0.840	0.009
	Acetate (1.93)	50693 (18772)	44858 (14485)	49914 (17337)	44744 (19436)	57549 (52051)	44403 (18625)	0.947	0.840	0.733	0.921	0.889	0.852	0.921
	N-acetylglycoproteins (2.06)	406216 (40973)	407386 (63967)	419361 (42024)	420502 (37053)	420976 (37370)	418974 (45877)	0.145	0.058	0.233	0.894	0.159	0.429	0.919
	Pyruvate (2.39)	113971 (47615)	104819 (60344)	117431 (39816)	113641 (50784)	157587 (65522)	139496 (81863)	0.000	0.000	0.476	0.007	0.000	0.647	0.032
	Glutamine (2.47)	78569 (14373)	75589 (19747)	78592 (16133)	79565 (21787)	72840 (13726)	74642 (19255)	0.125	0.085	0.700	0.102	0.212	0.840	0.230
	Citrate (2.57)	40201 (11046)	38336 (14196)	40861 (18797)	39180 (22052)	44473 (15573)	41101 (17161)	0.251	0.147	0.694	0.215	0.321	0.840	0.426
	Glycerophosphocholine (3.23)	409094 (85554)	392956 (91249)	387317 (93207)	367372 (124960)	335494 (82258)	330446 (93289)	0.000	0.000	0.181	0.020	0.000	0.384	0.071
	Phosphocholine (3.24)	279929 (89665)	258382 (120687)	255476 (72040)	232513 (105845)	228014 (79159)	204878 (91672)	0.001	0.000	0.285	0.063	0.002	0.467	0.167
	Acetoacetate (3.26)	34504 (12920)	30891 (10748)	34658 (14232)	31129 (13016)	34434 (11092)	31268 (9666)	0.862	0.611	0.870	0.734	0.786	0.908	0.852
	Lactate (4.13)	326492 (86828)	310850 (118247)	335334 (93764)	338124 (118908)	441637 (149057)	416682 (186958)	0.000	0.000	0.760	0.001	0.000	0.855	0.005
	Glycerol of lipid (5.22)	24526 (11687)	22139 (14880)	29752 (19841)	23335 (14684)	34556 (24859)	27257 (18468)	0.021	0.005	0.332	0.275	0.026	0.508	0.460
	a Glucose (5.25)	398621 (71492)	397024 (83845)	459255 (142926)	448856 (107237)	670555 (268768)	622376 (338630)	0.000	0.000	0.035	0.000	0.000	0.119	0.000
	Tyrosine (6.92)	19711 (3949)	19749 (4649)	23559 (6250)	23366 (8706)	21536 (5616)	20616 (6502)	0.019	0.082	0.006	0.190	0.211	0.027	0.391
	Histidine (7.08)	18707 (2835)	18921 (3420)	17784 (2845)	18044 (3170)	17502 (3703)	17272 (5254)	0.089	0.049	0.097	0.747	0.141	0.225	0.853
	Phenylalanine (7.44)	15265 (2107)	15052 (2650)	16151 (3021)	16318 (4517)	15553 (2757)	15710 (3740)	0.413	0.381	0.233	0.488	0.563	0.429	0.650
	Formate (8.48)	7203 (1785)	7182 (2867)	7312 (1844)	6783 (2378)	7305 (1757)	7111 (2383)	0.950	0.779	0.822	0.907	0.863	0.889	0.920

		No (n=27)		IGT (n=12)		T2D (n=41)		Kruskal Wallis	Mann Whitney U Test			Mann Whitney U Test (BH pFDR)		
	Metabolite (selected ppm)	Mean RI (SD)	Median RI (IQR)	Mean RI (SD)	Median RI (IQR)	Mean RI (SD)	Median RI (IQR)	No Vs IGT Vs T2D	No Vs T2D	No Vs IGT	IGT VS T2D	No Vs T2D	No Vs IGT	IGT VS T2D
Urine	2-Aminobutyrate (0.98)	64761 (23500)	59275 (19928)	64085 (22257)	59345 (21639)	62856 (19066)	57799 (20328)	0.992	0.921	1.000	0.925	0.989	1.000	0.989
	Valine(1.00)	80442 (21329)	77000 (20665)	78435 (22816)	80578 (19978)	77780 (27843)	77709 (29499)	0.884	0.654	0.964	0.761	0.943	0.989	0.989
	Isobutyrate(1.11)	216504 (56607)	211059 (74933)	235301 (139682)	185610 (95081)	181885 (65450)	171858 (78056)	0.056	0.017	0.599	0.297	0.122	0.916	0.776
	3-Hydroxyisovalerate (1.27)	403277 (199345)	368576 (140655)	394378 (154372)	368160 (165217)	362131 (186556)	302336 (155670)	0.217	0.104	0.869	0.287	0.402	0.989	0.776
	Lactate (1.34)	632763 (504349)	470381 (311061)	556812 (198465)	538320 (265310)	968406 (1070491)	553020 (844978)	0.803	0.542	0.869	0.714	0.895	0.989	0.980
	Alanine (1.49)	543120 (219892)	468674 (193956)	554490 (150200)	506428 (191015)	549226 (250895)	472186 (279981)	0.709	0.803	0.518	0.443	0.989	0.893	0.835
	Acetate (1.93)	2324476 (6094757)	524619 (304731)	2352118 (3043757)	555563 (2845122)	1367491 (3766551)	396371 (345540)	0.125	0.203	0.443	0.056	0.586	0.835	0.269
	4-Cresyl sulfate (2.34)	568611 (332439)	468195 (496426)	486832 (648392)	248966 (239728)	557666 (375053)	526470 (555218)	0.165	0.690	0.039	0.153	0.964	0.209	0.496
	Citrate (2.55)	3064680 (1390103)	2838086 (1210546)	2923704 (1955514)	2180146 (1148961)	2568489 (1415795)	2309597 (1628168)	0.289	0.130	0.343	0.809	0.458	0.806	0.989
	Trimethylamine (2.87)	297189 (129243)	259403 (105182)	334965 (136934)	303879 (243806)	234353 (112218)	202805 (158012)	0.018	0.026	0.578	0.019	0.149	0.916	0.125
	Dimethylglycine (2.93)	532219 (290342)	418560 (325528)	525608 (249159)	408607 (207690)	504381 (242742)	486091 (278264)	0.938	0.794	0.916	0.777	0.989	0.989	0.989
	Proline Betaine (3.11)	529607 (683973)	237647 (428844)	436096 (412533)	301315 (190485)	447079 (420997)	272501 (389820)	0.801	0.636	0.480	0.941	0.938	0.864	0.989
	Carnitine (3.23)	911079 (786742)	649546 (567763)	784167 (401216)	813820 (525884)	670208 (358711)	659215 (560928)	0.528	0.324	0.964	0.430	0.796	0.989	0.835
	TMAO (3.28)	5111915 (5300440)	3238925 (3655856)	5270472 (3572057)	4033048 (5258910)	5755435 (5728612)	4876206 (3536581)	0.617	0.356	0.518	0.992	0.806	0.893	1.000
	Glycine (3.57)	3892749 (2654922)	3375945 (1449371)	3979506 (2917137)	3250586 (1289335)	2377987 (2044935)	1892495 (1412042)	0.000	0.000	0.940	0.003	0.001	0.989	0.044
	Creatine (3.94)	1864468 (3294615)	878880 (908736)	2491284 (3666239)	735602 (1856611)	1048080 (1217035)	566047 (431066)	0.018	0.004	0.663	0.201	0.044	0.943	0.586
	Hippurate (3.97)	1517289 (743116)	1397574 (623214)	1356217 (653000)	1150030 (587977)	1619546 (884685)	1347041 (1401539)	0.689	0.940	0.391	0.468	0.989	0.833	0.862
	Creatinine (4.06)	14119711 (3009093)	13720578 (3729354)	12559120 (2769626)	12327916 (3571226)	11176842 (2819035)	11525132 (3096706)	0.000	0.000	0.104	0.147	0.001	0.402	0.496
	Methylnicotinate (4.44)	307546 (166536)	270277 (198881)	533780 (450363)	287597 (733141)	367688 (201728)	301383 (231171)	0.371	0.198	0.358	0.637	0.586	0.806	0.938
	B Glucose (4.65)	237787 (521030)	138461 (35310)	179162 (79432)	152232 (47872)	8284935 (19008607)	272102 (4926525)	0.000	0.000	0.075	0.009	0.000	0.323	0.082
	a Glucose (5.25)	150353 (245395)	98278 (29352)	122841 (37277)	115434 (27760)	3894927 (8674199)	192162 (2338839)	0.000	0.000	0.042	0.014	0.000	0.212	0.110
	4-Hydroxyphenylacetate (6.87)	109923 (32124)	99515 (41345)	126855 (60820)	113346 (46632)	116076 (54731)	105146 (59243)	0.729	0.931	0.391	0.536	0.989	0.833	0.895
	PAG (7.43)	287980 (139433)	280628 (195604)	294479 (233134)	225309 (244869)	328510 (170571)	286646 (225108)	0.503	0.432	0.599	0.317	0.835	0.916	0.796
	Indoxyl sulfate (7.70)	60970 (24389)	56970 (33977)	53731 (25032)	49335 (35631)	63431 (19861)	64845 (21764)	0.319	0.558	0.443	0.114	0.904	0.835	0.420
	Formate (8.46)	442205 (922663)	222061 (149467)	380070 (323294)	249050 (221932)	283380 (745654)	122163 (100561)	0.001	0.004	0.233	0.002	0.044	0.651	0.028
	Methylnicotinamide (9.28)	12388 (4808)	10962 (5309)	13927 (6713)	11779 (10472)	10222 (4266)	8799 (4851)	0.041	0.026	0.822	0.076	0.149	0.989	0.323

		No (n=27)		IGT (n=11)		T2D (n=42)		Kruskal Wallis	Mann Whitney U Test			Mann Whitney U Test (BH pFDR)		
	Metabolite (selected ppm)	Mean RI (SD)	Median RI (IQR)	Mean RI (SD)	Median RI (IQR)	Mean RI (SD)	Median RI (IQR)	No Vs IGT Vs T2D	No Vs T2D	No Vs IGT	IGT VS T2D	No Vs T2D	No Vs IGT	IGT VS T2D
Faeces	2-Methylbutyrate (0.86)	268377 (113896)	262815 (138462)	269357 (140090)	220281 (179791)	257510 (166722)	227292 (111506)	0.682	0.375	0.924	0.754	0.777	0.936	0.855
	Butyrate (0.90)	3457401 (2410171)	2636626 (3091482)	3736599 (3323791)	2769881 (2091661)	3073779 (2211840)	2437272 (2403064)	0.708	0.537	0.775	0.509	0.855	0.855	0.855
	Isovalerate (0.92)	632758 (272862)	549915 (270651)	541443 (314125)	473022 (483057)	628673 (451301)	535498 (338959)	0.409	0.389	0.213	0.481	0.777	0.699	0.853
	Leucine (0.96)	184213 (71309)	165076 (97849)	188267 (114333)	167531 (78266)	149687 (48746)	135894 (56736)	0.126	0.049	0.800	0.292	0.321	0.855	0.777
	Valine (0.99)	185165 (91648)	161769 (111986)	181971 (131544)	140803 (121496)	134298 (57480)	122519 (71598)	0.064	0.020	0.568	0.334	0.321	0.855	0.777
	Isoleucine (1.01)	134412 (59054)	124465 (81737)	138382 (94955)	117162 (88399)	105872 (41978)	95483 (46747)	0.109	0.035	0.849	0.345	0.321	0.877	0.777
	Propionate (1.06)	3885546 (2627335)	2827715 (2309545)	3653470 (2542682)	2734685 (3196206)	3262230 (1913027)	2826637 (2430918)	0.679	0.389	0.680	0.854	0.777	0.855	0.877
	Ethanol (1.18)	265217 (302191)	159659 (113940)	264356 (216301)	180450 (209165)	269802 (258894)	134700 (311622)	0.830	0.629	0.704	0.689	0.855	0.855	0.855
	Lactate (1.34)	319710 (246536)	246894 (96866)	320710 (278219)	221770 (163503)	228575 (183002)	171852 (81717)	0.002	0.001	0.590	0.070	0.013	0.855	0.420
	Lysine (1.72)	228852 (134551)	182841 (177939)	188614 (127574)	134324 (122670)	203337 (122468)	156002 (130411)	0.414	0.331	0.264	0.509	0.777	0.736	0.855
	Putrescine (1.77)	128737 (47063)	115871 (47998)	136455 (65622)	106197 (94833)	140531 (103037)	101235 (42660)	0.522	0.242	0.800	0.754	0.699	0.855	0.855
	Acetate (1.93)	24868120 (14252240)	19806156 (15081544)	22275036 (12008075)	19802355 (17973804)	19528903 (9463947)	18331165 (15420753)	0.342	0.164	0.800	0.442	0.673	0.855	0.820
	Valerate (2.17)	2364714 (1552715)	1862819 (2063790)	2607221 (2241879)	2030346 (1375954)	2089510 (1410437)	1632427 (1762836)	0.667	0.537	0.751	0.442	0.855	0.855	0.820
	Succinate (2.42)	372419 (929691)	149011 (123236)	154156 (112468)	111282 (98002)	1080363 (4107541)	137387 (87872)	0.373	0.691	0.190	0.236	0.855	0.673	0.699
	Methylamine (2.61)	117670 (42741)	101705 (79396)	116792 (43643)	111785 (41037)	104273 (36136)	96512 (43913)	0.403	0.228	1.000	0.380	0.699	1.000	0.777
	Trimethylamine (2.88)	189401 (231526)	121951 (91629)	270787 (271462)	165181 (135275)	179266 (185564)	120927 (93459)	0.246	0.784	0.149	0.113	0.855	0.673	0.616
	Dimethylbiguanide (3.05)	358779 (552238)	173365 (249689)	117575 (44380)	105157 (59104)	7053357 (5203156)	6823556 (5778478)	0.000	0.000	0.035	0.000	0.000	0.321	0.000
	Methanol (3.37)	641775 (540772)	543306 (424251)	638568 (504576)	496364 (368090)	535138 (429934)	468879 (434041)	0.639	0.382	0.849	0.596	0.777	0.877	0.855
	Glycine (3.57)	284473 (110925)	269294 (136900)	272538 (161159)	207984 (98487)	349324 (422222)	189848 (137734)	0.123	0.041	0.373	0.611	0.321	0.777	0.855
	a Glucose (5.24)	55260 (31623)	43157 (33809)	43255 (27425)	37786 (17164)	51710 (36298)	36937 (31939)	0.240	0.183	0.124	0.657	0.673	0.616	0.855
	Uracil (5.81)	31365 (13755)	28912 (16626)	22189 (7151)	21238 (6332)	24044 (6769)	23358 (6972)	0.055	0.047	0.045	0.442	0.321	0.321	0.820
	Fumarate (6.52)	30153 (15904)	24542 (22871)	29893 (19620)	19989 (27161)	27231 (12361)	22944 (12661)	0.834	0.765	0.634	0.626	0.855	0.855	0.855
	Tyrosine (6.91)	59148 (22500)	56741 (23363)	59713 (25871)	53514 (37098)	48412 (13031)	44900 (14669)	0.075	0.024	0.657	0.313	0.321	0.855	0.777
	Phenylacetate (7.31)	120854 (52910)	108197 (57064)	97848 (41132)	93701 (41811)	106709 (59050)	89843 (56268)	0.288	0.172	0.225	0.689	0.673	0.699	0.855
	Formate (8.46)	33191 (9398)	31212 (8862)	29129 (5161)	27822 (5632)	30888 (10088)	28533 (9270)	0.233	0.126	0.179	0.770	0.616	0.673	0.855

Supplementary Data 2 – Univariate analysis of semi-quantified (relative concentrations) of measured metabolites from serum, urine and faecal water ^{1}H-NMR experiments in participants with type 2 diabetes (T2D), impaired glucose tolerance (IGT) and participants without diabetes.

Diabetes – Targeted analyses

Supplementary Data 3 – Bile acids in faecal and serum samples in participants with type 2 diabetes (T2D), impaired glucose tolerance (IGT) and participants without diabetes

Faecal

	No diabetes (n=27)		IGT (n=11)		T2D (n=42)		Mann Whitney U Test			Mann Whitney U Test (BH pFDR)		
	Mean µM (SD)	Median µM (IQR)	Mean µM (SD)	Median µM (IQR)	Mean µM (SD)	Median µM (IQR)	No Vs T2D	No Vs IGT/T2D	No/IGT Vs T2D	No Vs T2D (pFDR)	No Vs IGT/T2D (pFDR)	No/IGT Vs T2D (pFDR)
All	2467291 (2206116)	2338273 (1732729)	2092381 (1364539)	1644535 (2139326)	2807605 (2430217)	2178571 (1151164)	0.976	0.832	0.649	0.985	0.949	0.868
All [1]	322324 (1156474)	63920 (133080)	461802 (905584)	41340 (154700)	598684 (1608946)	43740 (63525)	0.394	0.490	0.384	0.876	0.874	0.844
All [2]	1748871 (1150669)	1752020 (1389290)	1115424 (872393)	820960 (803790)	1594301 (799818)	1604750 (940825)	0.604	0.336	0.698	0.920	0.874	0.868
All [1c]	49373 (115588)	14643 (53541)	54826 (62289)	25743 (81655)	89685 (202133)	18093 (68474)	0.746	0.649	0.822	0.920	0.874	0.898
All [2c]	2687 (1892)	1475 (2361)	1843 (1676)	1520 (1501)	3322 (3590)	1961 (2824)	0.374	0.576	0.216	0.876	0.874	0.771
Others	344035 (433669)	295128 (254430)	458486 (585200)	186095 (249280)	521613 (823700)	293141 (137551)	0.719	0.879	0.401	0.920	0.949	0.844
[1] Cholic acid (CA)	162614 (779207)	8420 (46260)	253018 (530181)	13080 (36540)	381707 (1118407)	6100 (17460)	0.300	0.556	0.134	0.876	0.874	0.658
[1] Chenodeoxycholic acid (CDCA)	159710 (378293)	53460 (104620)	208784 (385514)	28260 (120250)	216977 (498089)	36660 (44040)	0.425	0.480	0.501	0.876	0.874	0.866
CA:CDCA ratio	0.48 (0.72)	0.34 (0.51)	0.58 (0.57)	0.34 (0.41)	0.51 (0.73)	0.17 (0.4)	0.587	0.832	0.307	0.920	0.949	0.844
[1c] Glycocholic Acid (GCA)	1586 (3688)	663 (804)	1016 (1039)	738 (868)	4244 (10996)	656 (2560)	0.294	0.348	0.271	0.876	0.874	0.844
[1c] Taurocholic acid (TCA)	275 (1051)	55 (288)	693 (1162)	110 (784)	781 (1819)	83 (516)	0.243	0.236	0.405	0.876	0.874	0.844
[1c] Glycochenodeoxycholic acid (GCDCA)	2540 (2963)	1040 (2185)	1407 (1173)	1073 (1621)	4707 (9849)	970 (3210)	0.730	0.858	0.606	0.920	0.949	0.866
[1c] Taurochenodeoxycholic acid (TCDCA)	44972 (108422)	13483 (51948)	51710 (59382)	22900 (79088)	79953 (182120)	16174 (58889)	0.728	0.620	0.844	0.920	0.874	0.898
G:T ratio	0.17 (0.13)	0.08 (0.16)	0.09 (0.07)	0.06 (0.06)	0.14 (0.09)	0.12 (0.09)	0.301	0.571	0.074	0.876	0.874	0.529
G:T-CA ratio	9.22 (5.06)	5.43 (10.81)	6.14 (5.60)	3.41 (6.50)	8.03 (6.43)	6.4 (5.91)	0.939	0.644	0.644	0.985	0.874	0.868
G:T-CDCA ratio	0.11 (0.06)	0.05 (0.09)	0.06 (0.05)	0.04 (0.04)	0.08 (0.07)	0.06 (0.05)	0.812	0.911	0.345	0.923	0.949	0.844
Conjugated CA:CA ratio	0.28 (0.12)	0.05 (0.13)	0.08 (0.13)	0.03 (0.03)	0.36 (0.71)	0.15 (0.31)	0.066	0.206	0.019	0.876	0.874	0.529
Conjugated CDCA:CDCA ratio	0.77 (1.29)	0.27 (0.95)	0.76 (1.16)	0.28 (0.52)	1.08 (1.74)	0.35 (0.82)	0.375	0.448	0.365	0.876	0.874	0.844
[1c]:[1] ratio	0.55 (0.86)	0.22 (0.69)	0.56 (0.9)	0.17 (0.32)	0.87 (1.51)	0.28 (0.52)	0.438	0.578	0.311	0.876	0.874	0.844
[2] Deoxycholic acid (DCA)	1136973 (926723)	1048080 (1012470)	687191 (617921)	506280 (645970)	1076299 (595848)	1006130 (756690)	0.784	0.454	0.511	0.920	0.874	0.866
[2] Lithocholic acid	575900 (266666)	474440 (368920)	380264 (293610)	326300 (224080)	474893 (289750)	490330 (278065)	0.252	0.135	0.748	0.876	0.874	0.890
[2] Ursodeoxycholic acid (UDCA)	35998 (52560)	18160 (46910)	47969 (94901)	13000 (33510)	43109 (96054)	13680 (18815)	0.475	0.448	0.582	0.880	0.874	0.866
[2]:[1] ratio	32.6 (41.7)	23.5 (34.9)	24.1 (21.2)	19.2 (26.9)	45.4 (35.9)	42.3 (45.4)	0.112	0.256	0.030	0.876	0.874	0.529
DCA:CA ratio	219 (321)	66 (206)	96 (135)	44 (107)	389 (886)	199 (344)	0.160	0.357	0.045	0.876	0.874	0.529
LCA:CDCA ratio	19.1 (40.6)	12.1 (15.2)	11.2 (8.1)	11.6 (11.7)	19.6 (19.1)	15.7 (16.2)	0.325	0.479	0.177	0.876	0.874	0.681
UDCA:CDCA ratio	0.47 (0.44)	0.28 (0.50)	0.34 (0.17)	0.34 (0.24)	0.54 (0.55)	0.37 (0.31)	0.430	0.551	0.340	0.876	0.874	0.844
LCA:UDCA ratio	38.1 (30.1)	35.6 (46)	36.1 (30.1)	30.8 (45.6)	39.1 (28.0)	36.9 (41.1)	0.937	0.984	0.829	0.985	0.984	0.898
[2c] Glycodeoxycholic acid (GDCA)	1516 (1506)	853 (1346)	1043 (960)	710 (1393)	1826 (1585)	1274 (2085)	0.308	0.552	0.154	0.876	0.874	0.658
[2c] Glycoursodeoxycholic acid (GUDCA)	204 (180)	128 (211)	193 (244)	100 (115)	595 (1949)	98 (343)	0.640	0.633	0.703	0.920	0.874	0.868
[2c] Glycolithocholic acid (GLCA)	118 (99)	90 (96)	97 (131)	48 (48)	94 (126)	68 (58)	0.180	0.117	0.606	0.876	0.874	0.866
[2c] Taurodeoxycholic acid (TDCA)	781 (348)	170 (359)	343 (711)	95 (148)	720 (1707)	331 (667)	0.206	0.506	0.067	0.876	0.874	0.529
[2c] Tauro-ursodeoxycholic acid (TUDCA)	68 (60)	23 (41)	167 (389)	25 (56)	88 (143)	26 (56)	0.757	0.838	0.682	0.920	0.949	0.868
2c:2 ratio	0.0017 (0.0017)	0.0014 (0.0017)	0.0023 (0.0021)	0.0013 (0.0022)	0.0027 (0.0041)	0.0012 (0.0027)	0.252	0.424	0.156	0.876	0.874	0.658
Conjugated DCA:DCA ratio	0.0031 (0.0052)	0.0015 (0.0022)	0.0028 (0.0026)	0.0017 (0.002)	0.0029 (0.0031)	0.0016 (0.0031)	0.985	0.911	0.800	0.985	0.949	0.898
Conjugated UDCA:UDCA ratio	0.018 (0.055)	0.007 (0.019)	0.014 (0.017)	0.006 (0.007)	0.02 (0.035)	0.007 (0.017)	0.755	0.872	0.712	0.920	0.949	0.868
G:T-DCA ratio	8.37 (5.30)	4.98 (9.51)	8.37 (12.57)	3.16 (6.84)	6.53 (6.45)	4.66 (4.76)	0.612	0.537	0.905	0.920	0.874	0.923
G:T-UDCA ratio	5.99 (4.77)	3.48 (5.15)	3.18 (2.01)	2.57 (2.09)	5.95 (8.91)	3.43 (4.02)	0.585	0.422	0.952	0.920	0.874	0.952
Isolithocholic acid	77590 (89989)	60480 (57670)	55795 (39053)	54520 (41970)	75052 (49896)	66040 (55890)	0.791	0.974	0.444	0.920	0.984	0.866
3a-hydroxy-12 ketolithocholic acid (5ß-cholanic acid-3a-ol-12-one)	107092 (137671)	64100 (96720)	40969 (34397)	36160 (27540)	78059 (64260)	75720 (64515)	0.765	0.395	0.524	0.920	0.874	0.866
5ß-Cholanic acid-3ß, 12a-diol	57339 (41669)	53160 (47230)	36649 (33537)	27680 (27830)	52036 (30898)	48010 (32715)	0.475	0.261	0.874	0.880	0.874	0.910
3-Ketocholanic acid	16249 (24286)	11313 (12108)	8537 (6731)	8205 (6928)	13849 (11679)	10846 (13561)	0.946	0.800	0.543	0.985	0.949	0.866
Allolithocholic acid	4286 (7377)	3085 (2351)	2588 (2381)	2195 (1646)	4428 (2961)	4216 (3231)	0.273	0.664	0.071	0.876	0.874	0.529
3,6 // 3,12-Diketocholanic Acid	2967 (5711)	1048 (2186)	938 (1121)	608 (510)	2091 (2722)	1133 (1962)	0.915	0.569	0.528	0.985	0.874	0.866
12-Dehydrocholic acid	73465 (353430)	15890 (26253)	307454 (635678)	10713 (75970)	292717 (873949)	6724 (18183)	0.172	0.274	0.127	0.876	0.874	0.658
Murocholic acid (MuroCA)	3880 (5964)	1638 (6459)	5065 (11345)	1413 (3179)	2843 (6878)	565 (1681)	0.098	0.101	0.158	0.876	0.874	0.658
5a-Cholanic acid-3a-ol-6-one	944 (710)	543 (995)	341 (443)	203 (295)	367 (403)	236 (313)	0.007	0.003	0.072	0.350	0.150	0.529
9(11), (5B)-Cholenic Acid-3a-ol-12-one	201 (74)	155 (180)	130 (74)	128 (45)	152 (109)	140 (117)	0.224	0.168	0.462	0.876	0.874	0.866
Ursocholanic acid	9.3 (1.9)	2.5 (5)	5.9 (13.9)	2.5 (3.8)	3.8 (9)	2.5 (2.5)	0.353	0.372	0.394	0.876	0.874	0.844
Glyco-ursocholanic acid	7.2 (3)	7.5 (5)	8.4 (3.8)	7.5 (5)	8 (5.8)	7.5 (5)	0.505	0.393	0.767	0.902	0.874	0.892
Tauro-ursocholanic acid (TUCA)	7.8 (6.1)	5 (6.3)	6.4 (4.2)	5 (1.3)	8.6 (7.6)	5 (4.4)	0.695	0.826	0.565	0.920	0.949	0.866

Serum

	No diabetes (n=63)		IGT (n=24)		T2D (n=66)		Kruskall-Wallis	Mann Whitney U Test			Mann Whitney U Test (BH pFDR)	
	Mean µM (SD)	Median µM (IQR)	Mean µM (SD)	Median µM (IQR)	Mean µM (SD)	Median µM (IQR)	No Vs IGT Vs T2D	No Vs T2D	IGT Vs T2D	No Vs IGT	No Vs T2D (pFDR)	IGT Vs T2D (pFDR)
All	30967 (34713)	19555 (20870)	36846 (38412)	21887 (26394)	25708 (17602)	22508 (21146)	0.946	0.947	0.748	0.788	0.983	0.909
All 1	1332 (2160)	447 (1012)	1130 (1380)	589 (722)	1095 (1884)	365 (901)	0.418	0.412	0.228	0.470	0.647	0.694
All 2	937 (966)	606 (938)	884 (815)	664 (522)	1125 (1198)	700 (864)	0.700	0.426	0.601	0.891	0.651	0.909
All 1c	26574 (33685)	17161 (20657)	32964 (36777)	18532 (25425)	21596 (16613)	17945 (20697)	0.884	0.994	0.673	0.627	0.994	0.909
All 2c	1396 (1462)	980 (1406)	1336 (1871)	640 (1084)	1214 (1111)	1034 (1144)	0.559	0.776	0.396	0.287	0.858	0.871
All Others	727 (617)	516 (454)	532 (448)	410 (370)	677 (640)	484 (395)	0.127	0.245	0.278	0.046	0.499	0.694
[1] Cholic acid (CA)	665 (1173)	154 (473)	556 (761)	259 (499)	442 (1042)	91 (245)	0.016	0.022	0.014	0.459	0.119	0.243
[1] Chenodeoxycholic acid (CDCA)	667 (1236)	247 (569)	574 (767)	242 (410)	653 (979)	245 (625)	0.915	0.979	0.680	0.724	0.994	0.909
CA:CDCA ratio	1.235 (1.22)	0.709 (1.349)	1.175 (0.869)	1.119 (1.268)	0.737 (0.808)	0.402 (0.673)	0.001	0.002	0.005	0.568	0.037	0.243
[1c] Glycocholic Acid (GCA)	376 (496)	217 (243)	399 (429)	261 (441)	347 (378)	245 (283)	0.888	0.667	0.720	0.974	0.814	0.909
[1c] Taurocholic acid (TCA)	36.4 (59.8)	17.3 (28.2)	46.7 (62)	15.4 (45.2)	36.2 (66.1)	19.8 (27.9)	0.988	0.904	0.890	0.934	0.975	0.921
[1c] Glycochenodeoxycholic acid (GCDCA)	4969 (4331)	3964 (4555)	7544 (6645)	6143 (7080)	6134 (5061)	4261 (6328)	0.176	0.178	0.443	0.098	0.490	0.900
[1c] Taurochenodeoxycholic acid (TCDCA)	21193 (32151)	11880 (18761)	24974 (30486)	13439 (21240)	15078 (13814)	10237 (14442)	0.684	0.474	0.493	0.795	0.668	0.909
G:T ratio	0.422 (0.402)	0.284 (0.319)	0.541 (0.491)	0.351 (0.388)	0.568 (0.442)	0.446 (0.451)	0.040	0.012	0.528	0.196	0.102	0.909
G:T-CA ratio	13.95 (6.95)	12.04 (8)	11.64 (5.7)	9.84 (6)	15.26 (9.08)	13.64 (11.66)	0.239	0.780	0.139	0.104	0.858	0.588
G:T-CDCA ratio	0.398 (0.389)	0.26 (0.32)	0.523 (0.485)	0.334 (0.37)	0.541 (0.44)	0.414 (0.448)	0.046	0.016	0.641	0.159	0.110	0.909
Conjugated CA:CA ratio	3.49 (4.98)	1.68 (4.04)	2.37 (3.15)	1.03 (2.7)	5.39 (7.4)	2.69 (4.66)	0.030	0.037	0.022	0.500	0.145	0.243
TCA:CA	0.425 (1.229)	0.11 (0.321)	0.235 (0.359)	0.112 (0.167)	0.504 (0.683)	0.2 (0.67)	0.098	0.065	0.087	0.810	0.210	0.478
GCA:CA	3.07 (4.482)	1.419 (3.096)	2.137 (2.803)	0.92 (2.554)	4.884 (6.851)	2.415 (4.411)	0.024	0.026	0.022	0.549	0.119	0.243
Conjugated CDCA:CDCA ratio	226.8 (596.9)	69.9 (142.1)	117.3 (145.3)	60.2 (99.4)	165.9 (307.9)	62.1 (159.8)	0.953	0.923	0.734	0.861	0.976	0.909
1c:1 ratio	115.9 (367.2)	42.2 (85.5)	59.1 (58.7)	36.1 (78)	89.2 (113)	37.2 (97.1)	0.844	0.562	0.782	0.929	0.739	0.909
[2] Deoxycholic acid (DCA)	752 (845)	475 (694)	739 (754)	565 (478)	850 (1021)	567 (726)	0.921	0.678	0.946	0.876	0.814	0.946
[2] Lithocholic acid (LCA)	17.97 (14.97)	14.33 (12.52)	25.02 (33.35)	14.76 (17.57)	27.89 (40.03)	20.14 (19.55)	0.086	0.025	0.273	0.871	0.119	0.694
[2] Ursodeoxycholic acid (UDCA)	166 (179)	87 (189)	120 (96)	115 (100)	247 (486)	110 (191)	0.531	0.337	0.386	0.823	0.618	0.871
2:1 ratio	1.88 (1.859)	1.211 (1.977)	3.065 (7.078)	1.046 (2.373)	3.483 (4.444)	2.325 (3.908)	0.083	0.037	0.136	0.929	0.145	0.588
DCA:CA ratio	5.435 (8.299)	1.969 (5.659)	5.785 (10.259)	1.808 (4.266)	12.293 (16.658)	6.077 (14.782)	0.018	0.009	0.049	0.876	0.099	0.374
LCA:CDCA ratio	0.115 (0.14)	0.07 (0.13)	0.236 (0.729)	0.06 (0.132)	0.212 (0.299)	0.104 (0.267)	0.461	0.244	0.427	0.869	0.499	0.900
UDCA:CDCA ratio	0.559 (0.565)	0.379 (0.601)	1.019 (3.214)	0.288 (0.316)	0.881 (1.363)	0.492 (0.695)	0.144	0.215	0.054	0.419	0.499	0.374
UDCA:LCA ratio	12.59 (16.14)	7.11 (11.33)	15.39 (31.86)	5.64 (9.46)	63.02 (408.55)	4.55 (8.39)	0.600	0.391	0.881	0.419	0.633	0.921
LCA:UDCA ratio	0.248 (0.305)	0.141 (0.25)	0.287 (0.261)	0.179 (0.399)	0.261 (0.238)	0.22 (0.268)	0.600	0.391	0.881	0.419	0.633	0.921
[2c] Glycolithocholic acid (GLCA)	21.7 (23.9)	12.5 (24.7)	26 (29.7)	15.7 (28.8)	36.7 (66.3)	19.4 (33.7)	0.649	0.370	0.636	0.772	0.633	0.909
[2c] Glycodeoxycholic acid (GDCA)	937 (1109)	652 (939)	878 (1286)	419 (718)	824 (897)	575 (787)	0.670	0.744	0.510	0.381	0.853	0.909
[2c] Glycoursodeoxycholic acid (GUDCA)	167 (269)	98 (140)	115 (141)	63 (81)	139 (186)	74 (100)	0.286	0.222	0.546	0.183	0.499	0.909
[2c] Taurolithocholic acid (TLCA)	7.122 (16.563)	2.812 (5.225)	5.502 (7.471)	2.698 (5.064)	6.732 (7.804)	3.097 (9.396)	0.854	0.729	0.626	0.722	0.853	0.909
[2c] Tauro-ursodeoxycholic acid (TUDCA)	8.592 (14.14)	5.814 (7.505)	6.644 (7.956)	3.287 (5.406)	5.699 (9.125)	3.61 (4.779)	0.131	0.045	0.890	0.280	0.162	0.921
[2c] Taurodeoxycholic acid (TDCA)	255 (608)	100 (179)	306 (551)	88 (249)	202 (202)	125 (282)	0.811	0.564	0.639	0.944	0.739	0.909
2c:2 ratio	2.315 (2.826)	1.366 (1.515)	1.512 (1.194)	1.242 (1.261)	1.721 (1.848)	0.989 (1.759)	0.459	0.228	0.803	0.488	0.499	0.909
Conjugated DCA:DCAratio	2.372 (3.085)	1.342 (1.645)	1.523 (1.382)	1.235 (1.388)	1.857 (2.115)	1.023 (1.647)	0.529	0.332	0.810	0.387	0.618	0.909
Conjugated UDCA:UDCA ratio	1.77 (2.149)	1.004 (1.396)	1.204 (0.962)	0.938 (1.039)	1.213 (1.817)	0.641 (0.824)	0.042	0.013	0.221	0.536	0.102	0.694
Conjugated LCA:LCA ratio	1.882 (2.551)	1.321 (1.337)	1.36 (1.077)	1.023 (0.793)	1.465 (1.195)	1.147 (1.301)	0.829	0.681	0.789	0.574	0.814	0.909
G:T-DCA ratio	6.01 (3.499)	5.357 (3.878)	4.601 (2.739)	4.624 (3.994)	5.893 (3.963)	4.866 (4.646)	0.241	0.512	0.211	0.102	0.704	0.694
G:T-UDCA ratio	24.2 (14.3)	20.3 (17.9)	24.6 (17.2)	19.6 (16.3)	29.9 (21.8)	22.9 (28.2)	0.536	0.366	0.376	0.709	0.633	0.871
G:T-LCA ratio	6.973 (12.743)	4.571 (5.099)	6.498 (3.766)	5.794 (3.486)	13.537 (31.642)	4.842 (5.793)	0.295	0.173	0.904	0.224	0.490	0.921
Glycohyodeoxycholic acid (GHDCA)	166 (269)	98 (141)	113 (142)	57 (82)	137 (187)	75 (102)	0.299	0.228	0.540	0.196	0.499	0.909
Murocholic acid (MuroCA)	158 (207)	83 (111)	82 (63)	69 (62)	235 (391)	96 (141)	0.209	0.444	0.076	0.251	0.660	0.461
Taurohyocholic acid (THCA)	193 (295)	83 (153)	156 (221)	85 (138)	88 (111)	47 (84)	0.004	0.001	0.0499	0.632	0.028	0.374
12-Dehydrocholic acid	101.8 (253.8)	15.4 (39.3)	70.5 (174.5)	21.4 (35)	71.9 (96.3)	20.3 (116.1)	0.812	0.582	0.798	0.612	0.744	0.909
5ß-Cholanic acid-3ß, 12a-diol	30.1 (28.2)	19.5 (34.6)	35.5 (35.3)	22.7 (45.8)	54.9 (65.1)	31.4 (49.2)	0.125	0.047	0.250	0.768	0.162	0.694
5-Cholenic acid-3ß-ol	19.9 (7.2)	19.6 (9.4)	21.7 (9.4)	19.7 (12.2)	16.1 (6.7)	15.8 (9.1)	0.009	0.007	0.017	0.718	0.096	0.243
3a-hydroxy-7 ketolithocholic acid (5ß-cholanic acid-3a-ol-7-one)	18.9 (28)	7.8 (15)	14.3 (12.2)	11 (19.6)	16.7 (23.5)	6.7 (13.3)	0.659	0.473	0.458	0.732	0.668	0.900
Isolithocholic acid	12.7 (11.3)	8.9 (9.7)	13.9 (11.5)	13.1 (17)	19.1 (22.4)	11 (16.5)	0.489	0.230	0.702	0.652	0.499	0.909
3a-hydroxy-12 ketolithocholic acid (5ß-cholanic acid-3a-ol-12-one)	9.96 (12.56)	5.43 (12.33)	11.45 (16.41)	6.08 (10.33)	23.48 (48.04)	9.9 (14.26)	0.050	0.023	0.096	0.783	0.119	0.478
Glycohyocholic acid (GHCA)	8.01 (8.33)	5.51 (5.7)	5.45 (4.65)	3.99 (6.04)	4.52 (4.62)	3.04 (4.92)	0.003	0.001	0.189	0.204	0.028	0.694
Allolithocholic acid	7.11 (3.68)	6.54 (2.83)	6.6 (2.63)	5.87 (3.21)	7.57 (3.48)	6.65 (4.87)	0.425	0.313	0.265	0.676	0.615	0.694
8(14),(5ß)-Cholenic acid-3a, 12a-diol	1.32 (1.33)	0.95 (1.03)	1.82 (2.37)	0.72 (1.03)	2.11 (2.76)	1.03 (1.46)	0.183	0.084	0.240	0.761	0.257	0.694

Supplementary Data 3 – Measured bile acids in serum and faeces in participants with type 2 diabetes (T2D), impaired glucose tolerance (IGT) and participants without diabetes.

Supplementary Data 4 – SCFA and other carboxylic acids in faecal, urine and serum samples in participants with type 2 diabetes (T2D), impaired glucose tolerance (IGT) and participants without diabetes

Serum	No (n=64)		IGT (n=26)		T2D (n=66)		Kruskal Wallis	Mann Whitney U Test			Mann Whitney U Test (BH pFDR)		
	Mean µM (SD)	Median µM (IQR)	Mean µM (SD)	Median µM (IQR)	Mean µM (SD)	Median µM (IQR)	No Vs IGT Vs T2D	No Vs IGT	IGT Vs T2D	No Vs T2D	No Vs IGT	IGT Vs T2D	No Vs T2D
Acetic acid	50.0 (27.3)	45.0 (38.1)	48.9 (25.8)	44.9 (18.1)	53.6 (34.1)	48.4 (28.8)	0.889	0.905	0.651	0.730	0.946	0.919	0.946
Propionic acid	4.06 (1.86)	3.79 (2.33)	3.43 (1.64)	3.2 (1.9)	4.08 (1.96)	3.88 (3.46)	0.314	0.132	0.196	0.902	0.495	0.588	0.946
Isobutyric acid	1.20 (0.45)	1.17 (0.59)	1.10 (0.32)	1.09 (0.31)	1.21 (0.45)	1.19 (0.6)	0.564	0.341	0.323	0.876	0.695	0.695	0.946
Butyric acid	0.95 (0.51)	0.9 (0.65)	1.00 (0.52)	0.96 (0.5)	1.06 (0.69)	0.87 (0.66)	0.757	0.674	0.867	0.473	0.919	0.946	0.789
2 Methylbutyric acid	0.22 (0.04)	0.21 (0.05)	0.25 (0.09)	0.22 (0.08)	0.25 (0.06)	0.23 (0.07)	0.011	0.251	0.363	0.002	0.627	0.695	0.013
Isovaleric acid	0.33 (0.14)	0.31 (0.14)	0.43 (0.22)	0.37 (0.21)	0.40 (0.14)	0.37 (0.18)	0.003	0.021	0.887	0.001	0.106	0.946	0.010
Valeric acid	0.04 (0.04)	0.03 (0.07)	0.04 (0.04)	0.04 (0.06)	0.03 (0.06)	0.02 (0.05)	0.590	0.946	0.387	0.394	0.946	0.695	0.695
Caproic acid	0.24 (0.16)	0.24 (0.2)	0.22 (0.15)	0.22 (0.17)	0.23 (0.16)	0.20 (0.20)	0.795	0.515	0.935	0.615	0.814	0.946	0.919
Lactic acid	1675 (543)	1512 (670)	1790 (537)	1698 (726)	2429 (834)	2279 (1066)	0.000	0.243	0.000	0.000	0.627	0.003	0.000
2 Hydroxybutyric acid	47.1 (20.3)	43.5 (28.1)	55.6 (27.3)	51 (30.4)	69.1 (35.3)	59.6 (37.7)	0.000	0.184	0.051	0.000	0.588	0.219	0.001

Urine	No (n=27)		IGT (n=12)		T2D (n=44)		Kruskal Wallis	Mann Whitney U Test			Mann Whitney U Test (BH pFDR)		
	Mean µM (SD)	Median µM (IQR)	Mean µM (SD)	Median µM (IQR)	Mean µM (SD)	Median µM (IQR)	No Vs IGT Vs T2D	No Vs IGT	IGT Vs T2D	No Vs T2D	No Vs IGT	IGT Vs T2D	No Vs T2D
Acetic acid	1258 (2771)	508 (321)	1259 (1093)	648 (1356)	943 (2139)	455 (179)	0.091	0.091	0.025	0.865	0.529	0.201	0.907
A (Osmo norm)	1188 (2290)	475 (305)	1411 (1348)	880 (1545)	1098 (2400)	486 (503)	0.316	0.168	0.174	0.755	0.623	0.623	0.883
A (Creat norm)	1365 (3018)	462 (415)	1593 (1759)	852 (1725)	1407 (3818)	554 (452)	0.483	0.327	0.521	0.374	0.655	0.821	0.671
Propionic acid	96 (90)	75 (40)	101 (65)	79 (48)	79 (18)	79 (26)	0.745	0.499	0.483	0.930	0.802	0.791	0.951
P (Osmo norm)	94 (78)	74 (52)	108 (76)	78 (130)	99 (70)	84 (57)	0.793	0.578	0.821	0.584	0.832	0.902	0.832
P (Creat norm)	100 (106)	76 (62)	115 (88)	80 (151)	109 (81)	94 (51)	0.383	0.685	0.805	0.148	0.845	0.902	0.623
Isobutyric acid	12.3 (1.3)	12.2 (1.5)	13.2 (1.1)	13.2 (0.9)	11.9 (1.3)	11.8 (1.3)	0.006	0.045	0.001	0.169	0.339	0.036	0.623
IB (Osmo norm)	12.7 (6.9)	10.8 (4.6)	14.4 (6.2)	13.1 (7.6)	14.4 (8.2)	12.1 (7.8)	0.518	0.313	0.672	0.380	0.655	0.845	0.671
IB (Creat norm)	13.3 (9)	10.9 (5.6)	14.9 (7.9)	13.2 (9.4)	16 (9.6)	13.8 (9)	0.183	0.358	0.867	0.064	0.658	0.907	0.440
Butyric acid	10.5 (1.9)	10.2 (2.8)	11.5 (2.3)	10.7 (2.7)	9.5 (1.3)	9.5 (1.5)	0.004	0.199	0.003	0.023	0.639	0.059	0.201
B (Osmo norm)	10.6 (5)	8.9 (3.6)	12.4 (5)	12.6 (6.9)	11.4 (6.3)	9.8 (6.4)	0.490	0.199	0.358	0.846	0.639	0.658	0.907
B (Creat norm)	11.1 (6.7)	8.9 (5.4)	12.9 (6.7)	12.4 (9.9)	12.7 (7.4)	11.4 (7.2)	0.425	0.313	0.715	0.260	0.655	0.858	0.655
2 Methylbutyric acid	0.44 (0.22)	0.41 (0.22)	0.51 (0.28)	0.41 (0.44)	0.42 (0.24)	0.37 (0.23)	0.528	0.685	0.272	0.529	0.845	0.655	0.821
2MB (Osmo norm)	0.43 (0.23)	0.34 (0.29)	0.49 (0.19)	0.53 (0.35)	0.46 (0.28)	0.39 (0.26)	0.475	0.327	0.299	0.568	0.655	0.655	0.832
2MB (Creat norm)	0.45 (0.31)	0.34 (0.27)	0.5 (0.25)	0.48 (0.36)	0.5 (0.33)	0.42 (0.27)	0.385	0.284	0.821	0.222	0.655	0.902	0.646
Isovaleric acid	0.49 (0.27)	0.37 (0.26)	0.58 (0.32)	0.48 (0.29)	0.44 (0.19)	0.39 (0.22)	0.269	0.271	0.100	0.668	0.655	0.529	0.845
IV (Osmo norm)	0.46 (0.25)	0.39 (0.28)	0.57 (0.27)	0.57 (0.28)	0.49 (0.27)	0.44 (0.35)	0.294	0.118	0.246	0.552	0.559	0.652	0.828
IV (Creat norm)	0.49 (0.34)	0.37 (0.34)	0.59 (0.33)	0.55 (0.4)	0.53 (0.3)	0.52 (0.26)	0.378	0.327	0.601	0.222	0.655	0.832	0.646
Valeric acid	0.57 (0.17)	0.55 (0.18)	0.59 (0.18)	0.54 (0.22)	0.53 (0.12)	0.52 (0.18)	0.646	0.799	0.459	0.476	0.902	0.779	0.791
V (Osom norm)	0.58 (0.29)	0.47 (0.21)	0.64 (0.34)	0.63 (0.37)	0.65 (0.38)	0.52 (0.47)	0.826	0.620	0.961	0.592	0.837	0.972	0.832
V (Creat norm)	0.59 (0.35)	0.54 (0.35)	0.66 (0.38)	0.57 (0.56)	0.72 (0.43)	0.61 (0.55)	0.410	0.642	0.701	0.180	0.837	0.852	0.623
Caproic acid	1.48 (0.5)	1.41 (0.3)	1.46 (0.42)	1.43 (0.37)	1.35 (0.23)	1.31 (0.33)	0.351	0.730	0.289	0.231	0.864	0.655	0.650
C (Osmo norm)	1.51 (0.88)	1.23 (0.41)	1.54 (0.63)	1.55 (0.72)	1.64 (0.98)	1.44 (0.87)	0.690	0.343	0.883	0.552	0.658	0.913	0.828
C (Creat norm)	1.56 (1.03)	1.27 (0.93)	1.57 (0.75)	1.49 (0.93)	1.82 (1.13)	1.61 (0.98)	0.401	0.642	0.836	0.161	0.837	0.907	0.623
Lactic acid	263 (237)	204 (155)	221 (138)	195 (83)	462 (567)	265 (316)	0.309	0.642	0.223	0.240	0.837	0.646	0.652
L (Osmo norm)	248 (205)	168 (157)	235 (159)	180 (161)	497 (720)	274 (255)	0.041	1.000	0.096	0.022	1.000	0.529	0.201
L (Creat norm)	264 (268)	167 (226)	240 (165)	207 (197)	560 (772)	291 (354)	0.024	0.822	0.077	0.012	0.902	0.495	0.154
2 Hydroxybutyric acid	6.7 (6.3)	5.1 (2.9)	6 (2.1)	6.1 (3.2)	21.1 (26.9)	8.9 (23.5)	0.018	0.408	0.113	0.008	0.706	0.559	0.135
2HB (Osmo norm)	5.9 (4.6)	4.3 (2.7)	6 (2.3)	5.5 (1.6)	19.1 (23.1)	12.5 (18.7)	0.000	0.178	0.015	0.000	0.623	0.166	0.004
2HB (Creat norm)	5.7 (3.6)	4.3 (3.7)	6 (2.5)	5.6 (3.6)	21.9 (25.2)	14.9 (24.1)	0.000	0.358	0.010	0.000	0.658	0.153	0.004

Faeces	No (n=27)		IGT (n=11)		T2D (n=42)		Kruskal Wallis	Mann Whitney U Test			Mann Whitney U Test (BH pFDR)		
	Mean µM (SD)	Median µM (IQR)	Mean µM (SD)	Median µM (IQR)	Mean µM (SD)	Median µM (IQR)	No Vs IGT Vs T2D	No Vs IGT	IGT Vs T2D	No Vs T2D	No Vs IGT	IGT Vs T2D	No Vs T2D
Acetic Acid	50439 (31338)	48123 (49514)	48661 (26052)	43920 (31149)	41607 (18204)	38796 (24612)	0.714	0.899	0.611	0.460	0.939	0.902	0.902
Propionic Acid	19536 (11796)	16856 (11696)	19263 (8322)	19652 (11806)	16727 (7632)	14485 (11153)	0.592	0.680	0.416	0.452	0.902	0.902	0.902
Isobutyric Acid	1583 (809)	1420 (1191)	1394 (724)	1439 (1055)	1473 (802)	1473 (793)	0.877	0.657	0.888	0.691	0.902	0.939	0.902
Butyric Acid	18084 (10154)	17769 (17321)	23622 (22122)	15624 (9943)	16539 (10161)	14969 (11385)	0.606	0.751	0.416	0.460	0.939	0.902	0.902
2 Methylbutyric Acid	905 (550)	835 (935)	738 (459)	798 (539)	863 (556)	800 (462)	0.712	0.390	0.537	0.840	0.902	0.902	0.939
Isovaleric Acid	1372 (829)	1201 (1235)	1125 (708)	1262 (996)	1299 (811)	1203 (701)	0.855	0.546	0.689	0.908	0.902	0.902	0.939
Valeric Acid	2608 (1469)	2361 (2451)	1858 (1221)	1811 (1213)	2648 (1719)	2386 (1281)	0.299	0.190	0.134	0.821	0.902	0.902	0.939
Caproic Acid	596 (725)	215 (805)	552 (1087)	67 (506)	608 (844)	188 (767)	0.556	0.373	0.292	0.976	0.902	0.902	0.976
Lactic Acid	343 (232)	251 (99)	273 (109)	252 (100)	307 (331)	237 (72)	0.322	0.612	0.611	0.136	0.902	0.902	0.902
2 Hydroxybutyric Acid	16.8 (13.4)	13.3 (4.8)	17.0 (7.5)	14.9 (3.3)	16.6 (8.6)	14 (5.5)	0.446	0.308	0.626	0.307	0.902	0.902	0.902

Supplementary Data 4 – Measured SCFAs and other carboxylic acids in serum, urine and faeces of participants with type 2 diabetes (T2D), impaired glucose tolerance (IGT) and participants without diabetes.

Supplementary Data 5 - Serum metabolites in participants with type 2 diabetes (T2D), impaired glucose tolerance (IGT) and participants without diabetes

		No (n=64)		IGT (n=26)		T2D (n=66)		Kruskal Wallis	Mann Whitney U Test			Mann Whitney U Test (BH pFDR)		
		Mean µM (SD)	Median µM (IQR)	Mean µM (SD)	Median µM (IQR)	Mean µM (SD)	Median µM (IQR)	No Vs IGT Vs T2D	No Vs T2D	No Vs IGT	IGT Vs T2D	No Vs T2D	No Vs IGT	IGT Vs T2D
Acylcarnitines	Carnitine (C0)	43.6 (10.6)	43.3 (14.8)	47.3 (8.9)	44.5 (11.8)	45.4 (9)	45.3 (13.9)	0.373	0.373	0.160	0.492	0.631	0.387	0.731
	Acetylcarnitine (C2)	6.59 (2.76)	6.11 (2.89)	6.69 (1.89)	6.73 (3.14)	7.41 (2.44)	7.13 (2.32)	0.141	0.016	0.464	0.281	0.085	0.717	0.544
	Propionylcarnitine (C3)	0.345 (0.152)	0.31 (0.155)	0.42 (0.163)	0.411 (0.191)	0.461 (0.174)	0.413 (0.212)	0.002	0.000	0.019	0.414	0.000	0.092	0.676
	Hydroxybutyrylcarnitine (C4-OH)	0.068 (0.037)	0.057 (0.032)	0.099 (0.097)	0.072 (0.036)	0.099 (0.062)	0.084 (0.054)	0.002	0.000	0.134	0.124	0.000	0.354	0.338
	Hydroxypropionylcarnitine (C3-OH)	0.015 (0.004)	0.015 (0.006)	0.015 (0.005)	0.015 (0.006)	0.014 (0.005)	0.012 (0.007)	0.142	0.161	0.744	0.230	0.387	0.908	0.474
	Propenoylcarnitine (C3:1)	0.012 (0.003)	0.012 (0.004)	0.012 (0.003)	0.012 (0.005)	0.011 (0.004)	0.01 (0.006)	0.022	0.006	0.956	0.077	0.039	0.976	0.244
	Butyrylcarnitine (C4)	0.184 (0.075)	0.172 (0.073)	0.226 (0.119)	0.2 (0.095)	0.239 (0.128)	0.204 (0.084)	0.001	0.000	0.041	0.539	0.003	0.158	0.770
	Butenylcarnitine (C4:1)	0.014 (0.004)	0.013 (0.005)	0.014 (0.003)	0.013 (0.003)	0.014 (0.005)	0.013 (0.007)	0.419	0.691	0.754	0.998	0.881	0.908	1.000
	Valerylcarnitine (C5)	0.137 (0.055)	0.13 (0.068)	0.18 (0.062)	0.179 (0.095)	0.182 (0.061)	0.17 (0.084)	0.001	0.000	0.002	0.841	0.000	0.017	0.951
	Glutarylcarnitine (C6-OH)	0.024 (0.007)	0.023 (0.007)	0.025 (0.005)	0.025 (0.006)	0.028 (0.011)	0.026 (0.01)	0.107	0.040	0.139	0.652	0.158	0.361	0.852
	Methylglutarylcarnitine (C5-M-DC)	0.025 (0.018)	0.021 (0.005)	0.023 (0.003)	0.024 (0.005)	0.027 (0.012)	0.024 (0.007)	0.066	0.009	0.177	0.349	0.052	0.407	0.610
	Hydroxyvalerylcarnitine (C5-OH)	0.034 (0.007)	0.033 (0.009)	0.036 (0.006)	0.036 (0.008)	0.038 (0.009)	0.036 (0.008)	0.030	0.005	0.160	0.491	0.034	0.387	0.731
	Tiglylcarnitine (C5:1)	0.021 (0.005)	0.021 (0.008)	0.023 (0.005)	0.022 (0.005)	0.024 (0.006)	0.023 (0.006)	0.044	0.010	0.178	0.458	0.057	0.407	0.714
	Glutaconylcarnitine (C5:1-DC)	0.017 (0.004)	0.016 (0.006)	0.017 (0.003)	0.016 (0.006)	0.017 (0.005)	0.016 (0.006)	0.973	0.929	0.774	0.611	0.972	0.917	0.818
	Fumarylcarnitine (C6)	0.079 (0.032)	0.075 (0.028)	0.083 (0.032)	0.077 (0.029)	0.078 (0.026)	0.073 (0.03)	0.651	0.988	0.549	0.580	0.995	0.778	0.800
	Fumarylcarnitine (C6:1)	0.015 (0.004)	0.015 (0.005)	0.015 (0.003)	0.015 (0.004)	0.016 (0.004)	0.015 (0.005)	1.000	0.846	0.850	0.909	0.952	0.952	0.967
	Pimeloylcarnitine (C7-DC)	0.027 (0.013)	0.024 (0.012)	0.025 (0.009)	0.024 (0.013)	0.028 (0.012)	0.026 (0.014)	0.457	0.333	0.698	0.214	0.598	0.881	0.451
	Octanoylcarnitine (C8)	0.172 (0.111)	0.158 (0.069)	0.174 (0.107)	0.147 (0.063)	0.157 (0.068)	0.145 (0.055)	0.315	0.542	0.854	0.755	0.771	0.952	0.908
	Nonaylcarnitine (C9)	0.027 (0.014)	0.024 (0.013)	0.028 (0.011)	0.026 (0.009)	0.031 (0.013)	0.027 (0.015)	0.544	0.034	0.508	0.330	0.145	0.737	0.596
	Decanoylcarnitine (C10)	0.229 (0.179)	0.202 (0.116)	0.218 (0.17)	0.18 (0.106)	0.212 (0.165)	0.18 (0.107)	0.498	0.478	0.344	0.631	0.724	0.606	0.832
	Decenoylcarnitine (C10:1)	0.356 (0.143)	0.334 (0.162)	0.324 (0.124)	0.322 (0.125)	0.376 (0.124)	0.352 (0.18)	0.400	0.197	0.585	0.208	0.429	0.800	0.443
	Decadienylcarnitine (C10:2)	0.056 (0.016)	0.054 (0.016)	0.056 (0.013)	0.054 (0.01)	0.06 (0.018)	0.056 (0.02)	0.430	0.094	0.732	0.449	0.274	0.908	0.706
	Dodecanoylcarnitine (C12)	0.094 (0.034)	0.09 (0.031)	0.091 (0.027)	0.082 (0.026)	0.099 (0.082)	0.087 (0.033)	0.528	0.832	0.563	0.693	0.946	0.789	0.881
	Dodecanedioylcarnitine (C12-DC)	0.047 (0.009)	0.044 (0.009)	0.046 (0.008)	0.044 (0.015)	0.047 (0.011)	0.045 (0.01)	0.978	0.852	0.789	0.882	0.952	0.921	0.960
	Dodecenoylcarnitine (C12:1)	0.247 (0.075)	0.239 (0.1)	0.233 (0.081)	0.237 (0.081)	0.253 (0.083)	0.241 (0.094)	0.940	0.898	0.416	0.414	0.962	0.677	0.676
	Tetradecanoylcarnitine (C14)	0.063 (0.009)	0.062 (0.012)	0.065 (0.01)	0.061 (0.016)	0.065 (0.018)	0.064 (0.012)	0.605	0.785	0.656	0.807	0.921	0.855	0.932
	Tetradecenoylcarnitine (C14:1)	0.07 (0.019)	0.068 (0.028)	0.064 (0.016)	0.063 (0.022)	0.056 (0.014)	0.056 (0.018)	0.001	0.000	0.223	0.033	0.000	0.463	0.141
	Hydroxytetradecenoylcarnitine (C14:1-OH)	0.013 (0.003)	0.013 (0.003)	0.014 (0.004)	0.013 (0.005)	0.014 (0.004)	0.014 (0.003)	0.461	0.128	0.514	0.629	0.347	0.744	0.831
	Tetradecadienylcarnitine (C14:2)	0.027 (0.018)	0.024 (0.015)	0.026 (0.012)	0.022 (0.012)	0.025 (0.014)	0.022 (0.013)	0.580	0.608	0.942	0.745	0.816	0.975	0.908
	Hydroxytetradecadienylcarnitine (C14:2-OH)	0.006 (0.001)	0.007 (0.001)	0.007 (0.001)	0.007 (0.003)	0.007 (0.002)	0.007 (0.001)	0.911	0.753	0.987	0.821	0.908	0.995	0.941
	Hexadecanoylcarnitine (C16)	0.109 (0.025)	0.105 (0.033)	0.112 (0.029)	0.103 (0.042)	0.105 (0.025)	0.103 (0.034)	0.793	0.500	0.765	0.457	0.734	0.914	0.714
	Hydroxyhexadecanoylcarnitine (C16-OH)	0.006 (0.001)	0.006 (0.002)	0.006 (0.001)	0.006 (0.001)	0.008 (0.002)	0.007 (0.003)	0.000	0.000	0.397	0.002	0.000	0.659	0.018
	Hexadecenoylcarnitine (C16:1)	0.029 (0.01)	0.029 (0.011)	0.027 (0.009)	0.026 (0.012)	0.025 (0.013)	0.024 (0.011)	0.041	0.017	0.615	0.187	0.086	0.818	0.421
	Hydroxyhexadecenoylcarnitine (C16:1-OH)	0.011 (0.002)	0.011 (0.003)	0.01 (0.003)	0.01 (0.003)	0.011 (0.003)	0.011 (0.003)	0.449	0.346	0.751	0.231	0.607	0.908	0.474
	Hexadecadienylcarnitine (C16:2)	0.009 (0.003)	0.008 (0.003)	0.009 (0.003)	0.009 (0.003)	0.009 (0.005)	0.008 (0.003)	0.606	0.387	0.469	0.177	0.643	0.718	0.407
	Hydroxyhexadecadienylcarnitine (C16:2-OH)	0.013 (0.002)	0.013 (0.002)	0.014 (0.002)	0.014 (0.003)	0.014 (0.002)	0.013 (0.002)	0.731	0.443	0.463	0.880	0.701	0.717	0.960
	Octadecanoylcarnitine (C18)	0.036 (0.011)	0.035 (0.013)	0.035 (0.015)	0.032 (0.013)	0.037 (0.01)	0.037 (0.014)	0.091	0.583	0.494	0.264	0.800	0.731	0.522
	Octadecenoylcarnitine (C18:1)	0.128 (0.037)	0.125 (0.051)	0.126 (0.049)	0.117 (0.041)	0.11 (0.032)	0.104 (0.049)	0.074	0.006	0.502	0.167	0.035	0.735	0.393
	Hydroxyoctadecenoylcarnitine (C18:1-OH)	0.009 (0.002)	0.008 (0.003)	0.009 (0.002)	0.009 (0.003)	0.009 (0.002)	0.009 (0.003)	0.588	0.825	0.870	0.707	0.944	0.958	0.887
	Octadecadienylcarnitine (C18:2)	0.043 (0.014)	0.04 (0.014)	0.046 (0.021)	0.04 (0.022)	0.04 (0.014)	0.036 (0.017)	0.692	0.213	0.917	0.382	0.451	0.967	0.638
Amino Acids	Alanine	396 (83)	381 (100)	421 (78)	424 (65)	502 (100)	505 (109)	0.000	0.000	0.148	0.000	0.000	0.375	0.003
	Arginine	108 (21)	108 (23)	124 (32)	117 (27)	109 (21)	106 (23)	0.025	0.839	0.015	0.018	0.950	0.080	0.088
	Asparagine	41.5 (8.1)	39.6 (9.3)	45 (7.5)	43.9 (10.9)	45 (9.1)	42.9 (13.3)	0.067	0.032	0.054	0.885	0.140	0.196	0.960
	Aspartate	25.2 (6.4)	23.5 (7.9)	27.3 (5.6)	26.3 (8.5)	24.7 (4.6)	23.9 (5.5)	0.619	0.887	0.073	0.056	0.960	0.236	0.202
	Citrulline	27.6 (7.8)	27.3 (9.1)	33 (8.1)	29.9 (10)	23.5 (13)	20.9 (13.9)	0.000	0.001	0.005	0.000	0.012	0.031	0.000
	Glutamine	630 (99)	632 (150)	670 (93)	655 (117)	623 (97)	609 (140)	0.299	0.437	0.144	0.037	0.700	0.370	0.154
	Glutamate	84 (31)	78 (48)	113 (34)	108 (45)	114 (42)	110 (53)	0.005	0.000	0.000	0.899	0.000	0.004	0.962
	Glycine	228 (42)	217 (54)	241 (60)	228 (71)	228 (55)	225 (51)	0.203	0.776	0.483	0.481	0.917	0.726	0.725
	Histidine	81 (12)	80 (14)	84 (10)	84 (15)	81 (15)	79 (22)	0.898	0.803	0.340	0.277	0.929	0.604	0.539
	Isoleucine	77 (21)	72 (27)	97 (37)	86 (54)	105 (30)	98 (37)	0.000	0.000	0.030	0.201	0.000	0.134	0.435
	Leucine	142 (35)	136 (39)	179 (59)	159 (76)	186 (51)	172 (59)	0.000	0.000	0.006	0.283	0.000	0.036	0.544
	Lysine	209 (36)	217 (46)	222 (37)	220 (46)	225 (42)	224 (35)	0.154	0.005	0.196	0.357	0.034	0.429	0.614
	Methionine	20.9 (5.9)	20.9 (6.9)	24.4 (7.8)	22.2 (10.6)	25.1 (6.6)	24.1 (9)	0.002	0.000	0.061	0.467	0.003	0.215	0.718
	Ornithine	82 (25)	75 (30)	97 (19)	99 (31)	82 (21)	80 (29)	0.067	0.679	0.002	0.002	0.874	0.017	0.014
	Phenylalanine	81 (14)	81 (17)	90 (19)	86 (26)	88 (16)	86 (20)	0.187	0.019	0.068	0.899	0.092	0.233	0.962
	Proline	203 (73)	184 (77)	228 (81)	212 (105)	264 (83)	245 (92)	0.000	0.000	0.130	0.045	0.000	0.350	0.169
	Serine	116 (20)	115 (24)	128 (20)	126 (29)	116 (21)	115 (24)	0.199	0.953	0.022	0.014	0.976	0.102	0.076
	Threonine	117 (32)	116 (40)	131 (35)	124 (52)	122 (29)	121 (41)	0.562	0.351	0.149	0.289	0.612	0.376	0.550
	Tryptophan	60 (14)	60 (20)	68 (17)	68 (27)	63 (15)	61 (17)	0.177	0.352	0.043	0.192	0.612	0.166	0.426
	Tyrosine	74 (19)	73 (23)	93 (28)	89 (34)	85 (21)	82 (26)	0.005	0.001	0.001	0.411	0.011	0.014	0.676
	Valine	233 (52)	230 (67)	273 (66)	270 (87)	284 (55)	284 (70)	0.002	0.000	0.009	0.361	0.000	0.052	0.618
Biogenic Amines	Asymmetric dimethylarginine (ADMA)	0.551 (0.108)	0.557 (0.098)	0.549 (0.09)	0.551 (0.076)	0.554 (0.106)	0.551 (0.152)	0.946	0.847	0.736	0.634	0.952	0.908	0.834
	alpha-Aminoadipic acid	0.61 (0.53)	0.55 (0.7)	1.14 (1.23)	0.91 (0.86)	1.09 (0.88)	1 (1.19)	0.001	0.000	0.022	0.745	0.004	0.102	0.908
	cis-4-Hydroxyproline	0.072 (0.079)	0.1 (0.1)	0.096 (0.082)	0.1 (0.2)	0.083 (0.08)	0.1 (0.1)	0.533	0.408	0.194	0.478	0.674	0.428	0.724
	Creatinine	86 (74)	77 (18)	78 (16)	76 (18)	94 (52)	81 (38)	0.400	0.163	0.865	0.248	0.388	0.958	0.499
	Dihydroxyphenylalanine (DOPA)	0.186 (0.061)	0.2 (0.1)	0.2 (0.057)	0.2 (0)	0.205 (0.062)	0.2 (0)	0.404	0.088	0.292	0.772	0.263	0.552	0.917
	Dopamine	0.141 (0.108)	0.1 (0.125)	0.138 (0.11)	0.1 (0.175)	0.15 (0.115)	0.2 (0.275)	0.761	0.651	0.961	0.667	0.852	0.977	0.865
	Histamine	0.173 (0.045)	0.2 (0.1)	0.173 (0.045)	0.2 (0.075)	0.156 (0.05)	0.2 (0.1)	NA	0.045	1.000	0.159	0.168	1.000	0.387
	Kynurenine	2.895 (0.926)	2.68 (0.96)	3.324 (1.116)	3.18 (1.213)	3.49 (1.57)	3.06 (1.573)	0.343	0.062	0.073	0.834	0.217	0.236	0.947
	Methionine sulfoxide	0.478 (0.224)	0.467 (0.289)	0.559 (0.261)	0.549 (0.356)	0.695 (0.364)	0.67 (0.268)	0.002	0.000	0.283	0.061	0.001	0.544	0.215
	Nitrotyrosine	0.028 (0.045)	0 (0.1)	0.027 (0.045)	0 (0.075)	0.044 (0.05)	0 (0.1)	0.110	0.070	1.000	0.159	0.236	1.000	0.387
	Putrescine	0.13 (0.06)	0.1 (0.1)	0.13 (0.05)	0.1 (0.08)	0.14 (0.1)	0.1 (0.1)	0.942	0.898	0.985	0.933	0.962	0.995	0.972
	Sarcosine	1.15 (0.49)	1.08 (0.39)	1.34 (0.49)	1.27 (0.79)	1.36 (0.54)	1.32 (0.69)	0.013	0.003	0.071	0.818	0.024	0.236	0.940
	Symmetric dimethylarginine (SDMA)	0.444 (0.206)	0.406 (0.099)	0.423 (0.137)	0.406 (0.191)	0.485 (0.21)	0.406 (0.174)	0.748	0.450	0.672	0.308	0.706	0.867	0.572
	Serotonin	0.57 (0.441)	0.573 (0.549)	0.565 (0.433)	0.565 (0.449)	0.611 (0.454)	0.552 (0.581)	0.854	0.594	0.959	0.683	0.805	0.977	0.878
	Spermidine	0.177 (0.069)	0.167 (0.026)	0.187 (0.08)	0.167 (0.138)	0.152 (0.056)	0.159 (0.088)	0.358	0.083	0.586	0.088	0.256	0.800	0.263
	Spermine	0.123 (0.043)	0.1 (0)	0.115 (0.037)	0.1 (0)	0.112 (0.033)	0.1 (0)	0.224	0.110	0.570	0.735	0.310	0.796	0.908
	trans-4-Hydroxyproline	10.53 (5.1)	9.3 (5.82)	10.87 (5.18)	9.37 (7.33)	12.53 (6.07)	11.45 (8.5)	0.263	0.063	0.765	0.248	0.219	0.914	0.499
	Taurine	104 (30)	104 (39)	112 (30)	115 (40)	88 (32)	87 (47)	0.003	0.002	0.309	0.002	0.018	0.572	0.015
Glycerophospholipids	Lyso-phosphatidylcholine C14:0	4.98 (0.84)	4.98 (1.14)	4.9 (1.45)	5.1 (1.19)	4.93 (0.82)	4.92 (1.07)	0.795	0.801	0.854	0.735	0.929	0.952	0.908
	Lyso-phosphatidylcholine C16:0	65 (17)	63 (25)	65 (19)	66 (17)	58 (15)	55 (19)	0.033	0.018	0.952	0.079	0.090	0.976	0.247
	Lyso-phosphatidylcholine C16:1	2.47 (0.76)	2.45 (0.87)	2.38 (0.85)	2.32 (0.89)	2.11 (0.8)	1.92 (1.03)	0.014	0.004	0.695	0.105	0.029	0.881	0.298
	Lyso-phosphatidylcholine C17:0	1.21 (0.47)	1.17 (0.6)	1.13 (0.45)	1.1 (0.43)	1.04 (0.3)	1.08 (0.42)	0.453	0.066	0.440	0.497	0.226	0.700	0.731
	Lyso-phosphatidylcholine C18:0	20.5 (6.2)	20.1 (9.3)	20.4 (7)	20.1 (9.2)	16.7 (4.5)	16.4 (4.8)	0.001	0.000	0.882	0.011	0.004	0.960	0.059
	Lyso-phosphatidylcholine C18:1	15.2 (5.2)	14.7 (6.6)	14.3 (4.4)	14.5 (5.5)	13.1 (3.7)	12.1 (6.2)	0.021	0.006	0.497	0.182	0.039	0.731	0.414
	Lyso-phosphatidylcholine C18:2	21.2 (6.9)	20 (10.6)	22.2 (9.4)	21.6 (15)	19.2 (7)	18.2 (10.8)	0.312	0.097	0.762	0.166	0.281	0.914	0.391
	Lyso-phosphatidylcholine C20:3	2.05 (0.75)	1.93 (0.92)	2.06 (0.82)	2.23 (1.39)	1.76 (0.59)	1.66 (0.72)	0.011	0.027	0.799	0.094	0.121	0.929	0.274
	Lyso-phosphatidylcholine C20:4	4.81 (1.68)	4.53 (1.97)	4.69 (1.92)	4.59 (1.69)	4.43 (1.46)	4.08 (1.64)	0.263	0.175	0.789	0.550	0.406	0.921	0.779
	Lyso-phosphatidylcholine C24:0	0.265 (0.102)	0.234 (0.107)	0.268 (0.108)	0.247 (0.092)	0.287 (0.15)	0.261 (0.162)	0.782	0.910	0.952	0.906	0.967	0.976	0.966
	Lyso-phosphatidylcholine C26:0	0.545 (0.317)	0.445 (0.331)	0.534 (0.275)	0.474 (0.251)	0.692 (0.577)	0.527 (0.588)	0.400	0.382	0.868	0.580	0.638	0.958	0.800
	Lyso-phosphatidylcholine C26:1	0.243 (0.135)	0.204 (0.145)	0.253 (0.136)	0.23 (0.128)	0.314 (0.209)	0.262 (0.254)	0.373	0.084	0.616	0.324	0.256	0.818	0.588
	Lyso-phosphatidylcholine C28:0	0.41 (0.197)	0.352 (0.182)	0.408 (0.19)	0.362 (0.22)	0.461 (0.347)	0.388 (0.345)	0.940	0.942	0.858	0.930	0.975	0.952	0.972
	Lyso-phosphatidylcholine C28:1	0.475 (0.214)	0.421 (0.223)	0.472 (0.202)	0.433 (0.186)	0.524 (0.32)	0.476 (0.363)	0.940	0.722	0.945	0.848	0.904	0.976	0.952
	Diacyl-phosphatidylcholine C24:0	0.224 (0.125)	0.201 (0.13)	0.222 (0.119)	0.176 (0.137)	0.276 (0.194)	0.202 (0.242)	0.428	0.249	0.956	0.459	0.499	0.976	0.715
	Diacyl-phosphatidylcholine C26:0	1.177 (0.677)	1.105 (0.887)	1.215 (0.704)	1.085 (0.969)	1.565 (1.144)	1.23 (1.368)	0.651	0.063	0.896	0.250	0.217	0.962	0.499
	Diacyl-phosphatidylcholine C28:1	2.78 (0.745)	2.8 (1.013)	2.549 (0.49)	2.56 (0.573)	2.61 (0.748)	2.55 (0.8)	0.203	0.089	0.162	0.926	0.264	0.387	0.972
	Diacyl-phosphatidylcholine C30:0	3.574 (1.177)	3.4 (1.493)	3.666 (1.213)	3.595 (1.558)	3.409 (1.336)	3.17 (1.573)	0.207	0.266	0.775	0.262	0.525	0.917	0.521
	Diacyl-phosphatidylcholine C32:0	12.5 (3.2)	12.6 (3.7)	12.7 (3.2)	12.7 (4.7)	12.2 (3.7)	11.9 (4.4)	0.795	0.183	0.974	0.371	0.415	0.987	0.630
	Diacyl-phosphatidylcholine C32:1	17.8 (8.6)	16.6 (11)	18.2 (7.8)	18.7 (10.6)	19.4 (12)	16.8 (13.8)	0.897	0.789	0.803	0.885	0.921	0.929	0.960
	Diacyl-phosphatidylcholine C32:2	3.77 (1.44)	3.76 (1.9)	3.89 (1.41)	3.89 (2.05)	3.6 (1.45)	3.44 (1.65)	0.400	0.305	0.597	0.232	0.572	0.805	0.475
	Diacyl-phosphatidylcholine C32:3	0.392 (0.104)	0.37 (0.129)	0.385 (0.099)	0.364 (0.13)	0.344 (0.098)	0.331 (0.12)	0.142	0.008	0.813	0.072	0.050	0.936	0.236
	Diacyl-phosphatidylcholine C34:1	118 (15)	119 (20)	122 (14)	125 (13)	116 (23)	118 (18)	0.048	0.597	0.203	0.078	0.805	0.437	0.245
	Diacyl-phosphatidylcholine C34:2	128 (23)	130 (23)	134 (14)	136 (13)	129 (18)	129 (20)	0.151	0.886	0.156	0.082	0.960	0.386	0.256
	Diacyl-phosphatidylcholine C34:3	9.09 (3.14)	9.12 (3.94)	9.03 (2.6)	9.13 (2.65)	8.69 (3.29)	7.82 (4.15)	0.076	0.256	0.917	0.208	0.511	0.967	0.443
	Diacyl-phosphatidylcholine C34:4	1.052 (0.466)	1.004 (0.548)	1.096 (0.452)	1.05 (0.729)	1.028 (0.38)	0.97 (0.339)	0.651	0.578	0.585	0.307	0.800	0.800	0.572
	Diacyl-phosphatidylcholine C36:0	1.639 (0.985)	1.505 (0.989)	1.687 (0.877)	1.55 (1.05)	1.712 (0.862)	1.51 (1.01)	0.882	0.542	0.606	0.858	0.771	0.815	0.952
	Diacyl-phosphatidylcholine C36:1	55.338 (19.157)	53.35 (20.975)	53.969 (14.049)	54.25 (24.575)	49.18 (16.275)	45.55 (20.125)	0.263	0.027	0.921	0.083	0.123	0.969	0.256
	Diacyl-phosphatidylcholine C36:2	112.759 (13.06)	114.5 (17)	116.404 (12.281)	116 (13.25)	111.421 (16.194)	110.5 (20.25)	0.096	0.497	0.200	0.073	0.731	0.434	0.236
	Diacyl-phosphatidylcholine C36:3	92.489 (16.431)	95.05 (17.775)	93.058 (15.619)	96.85 (13.225)	88.209 (19.77)	89.25 (21.7)	0.127	0.131	0.931	0.148	0.350	0.972	0.375
	Diacyl-phosphatidylcholine C36:4	112.8 (14.7)	114 (16.5)	115.088 (18.028)	118.5 (12.75)	111 (17.865)	112 (18.75)	0.157	0.448	0.250	0.073	0.706	0.499	0.236
	Diacyl-phosphatidylcholine C36:5	14.889 (6.762)	14.15 (9.063)	15.343 (6.976)	14.7 (5.7)	15.915 (8.741)	14.5 (6.6)	0.897	0.650	0.702	0.916	0.852	0.883	0.967
	Diacyl-phosphatidylcholine C36:6	0.402 (0.159)	0.379 (0.196)	0.41 (0.175)	0.396 (0.258)	0.379 (0.143)	0.349 (0.164)	0.622	0.271	0.827	0.373	0.529	0.944	0.631
	Diacyl-phosphatidylcholine C38:0	2.147 (0.527)	2.055 (0.64)	2.201 (0.785)	2.01 (0.74)	1.874 (0.767)	1.635 (0.833)	0.109	0.001	0.886	0.038	0.011	0.960	0.155
	Diacyl-phosphatidylcholine C38:3	53.6 (15.8)	53.4 (17.7)	52.4 (16.6)	49.2 (22.7)	43.8 (13.4)	42.3 (11.3)	0.001	0.000	0.561	0.014	0.001	0.787	0.075
	Diacyl-phosphatidylcholine C38:4	88.6 (23.4)	86.9 (23.7)	87.9 (21.9)	84.9 (27.6)	84 (19.3)	85 (25)	0.619	0.184	0.699	0.492	0.415	0.881	0.731
	Diacyl-phosphatidylcholine C38:5	36.8 (10.3)	35.9 (13.4)	36 (13.2)	34.1 (13.7)	34.9 (10.8)	32.7 (11.8)	0.288	0.136	0.558	0.745	0.358	0.787	0.908
	Diacyl-phosphatidylcholine C38:6	49.5 (13.6)	48.7 (20.7)	49.5 (19.1)	47.4 (22.6)	46.5 (17.7)	41 (23.4)	0.373	0.084	0.689	0.431	0.256	0.881	0.696
	Diacyl-phosphatidylcholine C40:1	0.319 (0.059)	0.312 (0.081)	0.315 (0.052)	0.303 (0.053)	0.304 (0.056)	0.291 (0.052)	0.247	0.117	1.000	0.159	0.321	1.000	0.387
	Diacyl-phosphatidylcholine C40:2	0.342 (0.084)	0.331 (0.109)	0.332 (0.091)	0.291 (0.105)	0.317 (0.078)	0.304 (0.083)	0.141	0.051	0.472	0.671	0.187	0.719	0.867
	Diacyl-phosphatidylcholine C40:3	0.558 (0.153)	0.547 (0.162)	0.516 (0.11)	0.5 (0.141)	0.491 (0.112)	0.475 (0.141)	0.013	0.001	0.107	0.343	0.012	0.303	0.606
	Diacyl-phosphatidylcholine C40:4	3.75 (1.28)	3.56 (1.52)	3.58 (1.16)	3.62 (1.54)	3.32 (1.03)	3.19 (1.47)	0.076	0.022	0.616	0.333	0.102	0.818	0.598
	Diacyl-phosphatidylcholine C40:5	8.32 (2.57)	8.03 (2.61)	8.15 (3.06)	8.56 (3.39)	7.63 (2.42)	7.2 (3.25)	0.142	0.114	0.830	0.505	0.315	0.946	0.736
	Diacyl-phosphatidylcholine C40:6	20.9 (5.5)	20.3 (6.8)	21.9 (10.4)	20.2 (8.5)	18.5 (7.5)	16.5 (6.9)	0.005	0.005	0.732	0.127	0.033	0.908	0.345
	Diacyl-phosphatidylcholine C42:0	0.447 (0.129)	0.428 (0.145)	0.412 (0.101)	0.428 (0.146)	0.379 (0.111)	0.376 (0.156)	0.033	0.001	0.284	0.111	0.006	0.545	0.312
	Diacyl-phosphatidylcholine C42:1	0.218 (0.057)	0.208 (0.078)	0.201 (0.047)	0.208 (0.066)	0.188 (0.05)	0.18 (0.075)	0.142	0.003	0.377	0.224	0.021	0.635	0.463
	Diacyl-phosphatidylcholine C42:2	0.196 (0.044)	0.192 (0.044)	0.196 (0.052)	0.182 (0.058)	0.177 (0.04)	0.17 (0.048)	0.020	0.004	0.745	0.139	0.029	0.908	0.361
	Diacyl-phosphatidylcholine C42:4	0.191 (0.04)	0.188 (0.055)	0.184 (0.045)	0.173 (0.051)	0.17 (0.033)	0.167 (0.039)	0.028	0.002	0.292	0.270	0.014	0.552	0.528
	Diacyl-phosphatidylcholine C42:5	0.294 (0.068)	0.284 (0.063)	0.288 (0.097)	0.261 (0.103)	0.266 (0.071)	0.261 (0.079)	0.067	0.006	0.248	0.473	0.037	0.499	0.719
	Diacyl-phosphatidylcholine C42:6	0.459 (0.126)	0.455 (0.121)	0.452 (0.141)	0.454 (0.137)	0.42 (0.109)	0.398 (0.143)	0.029	0.029	0.660	0.268	0.132	0.858	0.526
	Acyl-alkyl-phosphatidylcholine C30:0	0.298 (0.085)	0.286 (0.096)	0.273 (0.08)	0.265 (0.101)	0.273 (0.1)	0.257 (0.103)	0.215	0.039	0.213	0.774	0.158	0.451	0.917
	Acyl-alkyl-phosphatidylcholine C30:1	0.245 (0.126)	0.231 (0.137)	0.267 (0.146)	0.235 (0.156)	0.284 (0.196)	0.231 (0.192)	1.000	0.574	0.561	0.940	0.799	0.787	0.975
	Acyl-alkyl-phosphatidylcholine C30:2	0.13 (0.041)	0.123 (0.05)	0.124 (0.034)	0.128 (0.042)	0.128 (0.048)	0.119 (0.06)	0.444	0.468	0.893	0.788	0.718	0.962	0.921
	Acyl-alkyl-phosphatidylcholine C32:1	2.31 (0.54)	2.22 (0.62)	2.11 (0.39)	2.09 (0.46)	1.97 (0.47)	1.96 (0.56)	0.019	0.000	0.135	0.098	0.001	0.356	0.282
	Acyl-alkyl-phosphatidylcholine C32:2	0.626 (0.16)	0.587 (0.18)	0.571 (0.115)	0.586 (0.2)	0.532 (0.141)	0.519 (0.154)	0.263	0.001	0.220	0.089	0.007	0.458	0.264
	Acyl-alkyl-phosphatidylcholine C34:0	1.194 (0.383)	1.155 (0.423)	1.111 (0.282)	1.155 (0.351)	1.147 (0.404)	1.105 (0.497)	0.403	0.516	0.531	0.778	0.744	0.761	0.917
	Acyl-alkyl-phosphatidylcholine C34:1	7.9 (2.39)	7.84 (3.02)	7.41 (1.33)	7.14 (2.18)	7 (2.16)	6.33 (2.66)	0.019	0.008	0.307	0.114	0.050	0.572	0.315
	Acyl-alkyl-phosphatidylcholine C34:2	8.07 (2.05)	7.85 (2.56)	7.28 (1.51)	7.11 (2.64)	6.46 (1.72)	6.2 (1.76)	0.000	0.000	0.131	0.017	0.000	0.350	0.088
	Acyl-alkyl-phosphatidylcholine C34:3	4.95 (1.42)	4.92 (2.02)	4.64 (1.3)	4.88 (1.4)	3.94 (1.17)	3.72 (1.25)	0.000	0.000	0.442	0.014	0.000	0.701	0.076
	Acyl-alkyl-phosphatidylcholine C36:0	0.635 (0.141)	0.623 (0.155)	0.657 (0.264)	0.606 (0.151)	0.602 (0.167)	0.599 (0.195)	0.471	0.199	0.686	0.586	0.433	0.879	0.800
	Acyl-alkyl-phosphatidylcholine C36:1	16.5 (4.8)	16.6 (4.8)	15 (4.3)	15.4 (4.8)	15.8 (4)	14.9 (5.7)	0.261	0.311	0.358	0.902	0.573	0.614	0.964
	Acyl-alkyl-phosphatidylcholine C36:2	9.6 (2.8)	9.4 (3.1)	9.1 (2.1)	8.8 (2.5)	8.8 (2.9)	8.7 (3.1)	0.256	0.094	0.591	0.434	0.274	0.804	0.698
	Acyl-alkyl-phosphatidylcholine C36:3	5.21 (1.24)	5.14 (1.47)	4.64 (0.78)	4.48 (1.13)	3.95 (1.18)	3.65 (1.11)	0.000	0.000	0.038	0.001	0.000	0.155	0.010
	Acyl-alkyl-phosphatidylcholine C36:4	12.4 (2.9)	12.5 (4.4)	10.9 (2.1)	10.7 (2)	10.3 (3)	9.7 (3)	0.000	0.000	0.019	0.044	0.000	0.091	0.167
	Acyl-alkyl-phosphatidylcholine C36:5	7.7 (2.6)	7.3 (3.4)	7.3 (1.7)	7.2 (2.4)	7 (2.1)	6.5 (2)	0.014	0.016	0.365	0.188	0.083	0.623	0.421
	Acyl-alkyl-phosphatidylcholine C38:0	0.883 (0.254)	0.852 (0.348)	0.835 (0.262)	0.777 (0.368)	0.763 (0.228)	0.709 (0.283)	0.019	0.003	0.356	0.217	0.025	0.614	0.456
	Acyl-alkyl-phosphatidylcholine C38:1	1.634 (0.607)	1.545 (0.588)	1.546 (0.442)	1.585 (0.478)	1.484 (0.692)	1.46 (0.508)	0.400	0.054	0.752	0.162	0.196	0.908	0.387
	Acyl-alkyl-phosphatidylcholine C38:2	2.25 (0.68)	2.17 (0.7)	2.2 (0.43)	2.21 (0.59)	1.94 (0.56)	1.89 (0.5)	0.007	0.002	0.914	0.009	0.015	0.967	0.052
	Acyl-alkyl-phosphatidylcholine C38:3	5.74 (1.48)	5.8 (2.08)	5.25 (1.24)	5.03 (1.48)	4.94 (1.39)	4.65 (1.45)	0.000	0.000	0.087	0.150	0.004	0.263	0.376
	Acyl-alkyl-phosphatidylcholine C38:4	8.96 (2.31)	8.58 (3.07)	8.07 (1.5)	7.74 (1.75)	7.93 (2.16)	7.41 (2.33)	0.028	0.003	0.088	0.339	0.024	0.263	0.604
	Acyl-alkyl-phosphatidylcholine C38:5	11.4 (2.5)	11.2 (3.5)	10.5 (2.2)	10.4 (3)	9.5 (2.5)	9.1 (2.5)	0.000	0.000	0.140	0.024	0.000	0.361	0.111
	Acyl-alkyl-phosphatidylcholine C38:6	4.08 (0.98)	3.97 (1.32)	3.83 (0.98)	3.74 (0.81)	3.39 (1.12)	3.25 (1.43)	0.029	0.000	0.320	0.038	0.001	0.584	0.155
	Acyl-alkyl-phosphatidylcholine C40:1	1.064 (0.313)	1.01 (0.425)	1.03 (0.336)	0.985 (0.358)	1.02 (0.318)	0.985 (0.338)	0.782	0.379	0.517	0.943	0.637	0.744	0.975
	Acyl-alkyl-phosphatidylcholine C40:2	2.07 (0.63)	2.13 (0.67)	1.78 (0.35)	1.77 (0.47)	1.76 (0.39)	1.78 (0.53)	0.013	0.000	0.011	0.697	0.003	0.060	0.881
	Acyl-alkyl-phosphatidylcholine C40:3	1.5 (0.33)	1.44 (0.38)	1.34 (0.21)	1.31 (0.34)	1.24 (0.24)	1.24 (0.3)	0.000	0.000	0.031	0.071	0.000	0.136	0.236
	Acyl-alkyl-phosphatidylcholine C40:4	1.72 (0.37)	1.65 (0.46)	1.57 (0.27)	1.55 (0.34)	1.48 (0.32)	1.46 (0.37)	0.000	0.000	0.071	0.153	0.001	0.236	0.381
	Acyl-alkyl-phosphatidylcholine C40:5	3.22 (0.71)	3.09 (0.92)	2.85 (0.5)	2.78 (0.66)	2.73 (0.62)	2.71 (0.71)	0.005	0.000	0.022	0.320	0.001	0.102	0.584
	Acyl-alkyl-phosphatidylcholine C40:6	2.74 (0.73)	2.78 (1)	2.6 (0.5)	2.48 (0.52)	2.43 (0.72)	2.36 (0.83)	0.019	0.003	0.416	0.071	0.023	0.677	0.236
	Acyl-alkyl-phosphatidylcholine C42:0	0.61 (0.114)	0.607 (0.156)	0.585 (0.106)	0.557 (0.159)	0.591 (0.131)	0.57 (0.119)	0.428	0.437	0.324	0.505	0.700	0.588	0.736
	Acyl-alkyl-phosphatidylcholine C42:1	0.406 (0.131)	0.378 (0.161)	0.382 (0.128)	0.352 (0.14)	0.388 (0.15)	0.35 (0.175)	0.261	0.168	0.419	0.950	0.393	0.679	0.976
	Acyl-alkyl-phosphatidylcholine C42:2	0.504 (0.137)	0.493 (0.13)	0.456 (0.109)	0.423 (0.169)	0.436 (0.109)	0.439 (0.14)	0.080	0.002	0.075	0.439	0.015	0.238	0.700
	Acyl-alkyl-phosphatidylcholine C42:3	0.732 (0.182)	0.706 (0.167)	0.678 (0.16)	0.642 (0.256)	0.634 (0.16)	0.612 (0.157)	0.013	0.001	0.169	0.341	0.010	0.395	0.605
	Acyl-alkyl-phosphatidylcholine C42:4	0.702 (0.175)	0.658 (0.139)	0.609 (0.153)	0.579 (0.139)	0.572 (0.126)	0.563 (0.175)	0.001	0.000	0.005	0.522	0.000	0.033	0.750
	Acyl-alkyl-phosphatidylcholine C42:5	1.95 (0.38)	1.9 (0.4)	1.71 (0.29)	1.68 (0.46)	1.65 (0.34)	1.64 (0.5)	0.000	0.000	0.004	0.339	0.000	0.028	0.604
	Acyl-alkyl-phosphatidylcholine C44:3	0.147 (0.044)	0.138 (0.035)	0.136 (0.039)	0.121 (0.038)	0.14 (0.049)	0.124 (0.057)	0.148	0.114	0.193	0.974	0.315	0.426	0.987
	Acyl-alkyl-phosphatidylcholine C44:4	0.383 (0.104)	0.359 (0.103)	0.339 (0.076)	0.326 (0.088)	0.312 (0.077)	0.306 (0.075)	0.004	0.000	0.037	0.144	0.000	0.155	0.370
	Acyl-alkyl-phosphatidylcholine C44:5	1.38 (0.408)	1.33 (0.5)	1.201 (0.302)	1.19 (0.233)	1.118 (0.322)	1.1 (0.438)	0.001	0.000	0.013	0.313	0.000	0.072	0.576
	Acyl-alkyl-phosphatidylcholine C44:6	0.834 (0.217)	0.805 (0.222)	0.755 (0.191)	0.737 (0.269)	0.701 (0.178)	0.69 (0.233)	0.019	0.000	0.148	0.162	0.003	0.375	0.387
Sphingomyelins	Sphingomyelin ((OH) C14:1)	6.02 (1.52)	6.1 (1.86)	5.32 (1.7)	5.25 (1.97)	5.27 (1.47)	5.36 (1.5)	0.033	0.003	0.103	0.735	0.024	0.295	0.908
	Sphingomyelin ((OH) C16:1)	3.47 (0.9)	3.41 (1.11)	3.19 (0.76)	2.96 (1.1)	3.01 (0.68)	2.95 (0.8)	0.003	0.002	0.176	0.303	0.014	0.407	0.571
	Sphingomyelin ((OH) C22:1)	12 (3.1)	12.1 (4)	12.3 (3)	12.1 (4.2)	10.8 (2.4)	10.7 (3.1)	0.067	0.030	0.748	0.044	0.134	0.908	0.167
	Sphingomyelin ((OH) C22:2)	10.1 (2.3)	10.4 (3.5)	9.8 (2.5)	9.8 (3.5)	8.5 (2.1)	8.1 (2.8)	0.001	0.000	0.469	0.041	0.001	0.718	0.158
	Sphingomyelin ((OH) C24:1)	1.3 (0.36)	1.25 (0.49)	1.22 (0.28)	1.2 (0.34)	1.09 (0.27)	1.08 (0.36)	0.014	0.001	0.409	0.060	0.011	0.674	0.214
	Sphingomyelin (C16:0)	107 (18)	109 (20)	104 (17)	109 (17)	93 (18)	93 (23)	0.000	0.000	0.622	0.013	0.001	0.824	0.072
	Sphingomyelin (C16:1)	19.1 (3.8)	18.9 (4.8)	18.2 (5.1)	17.9 (6.5)	15.8 (3.9)	15.8 (5.8)	0.001	0.000	0.353	0.041	0.000	0.612	0.158
	Sphingomyelin (C18:0)	22.7 (5.1)	22.9 (7.4)	21.8 (6.6)	21.8 (6.1)	20 (4.8)	19 (6)	0.021	0.003	0.868	0.042	0.023	0.958	0.160
	Sphingomyelin (C18:1)	13.3 (3)	12.9 (4)	13.1 (4.1)	12.9 (3.6)	11.4 (3.4)	10.9 (3.9)	0.005	0.000	0.497	0.041	0.001	0.731	0.158
	Sphingomyelin (C20:2)	0.488 (0.182)	0.456 (0.192)	0.392 (0.146)	0.383 (0.131)	0.381 (0.108)	0.367 (0.106)	0.005	0.000	0.033	0.597	0.001	0.142	0.805
	Sphingomyelin (C24:0)	20.8 (5.1)	20.1 (6.7)	21.1 (5.3)	21.7 (7.3)	17.9 (4.3)	17.6 (5.8)	0.542	0.002	0.745	0.004	0.015	0.908	0.030
	Sphingomyelin (C24:1)	53.8 (9.1)	54.5 (12.6)	51.5 (8.8)	51.1 (9)	44.1 (12)	42.5 (14.7)	0.000	0.000	0.218	0.004	0.000	0.457	0.026
	Sphingomyelin (C26:0)	0.166 (0.042)	0.167 (0.058)	0.149 (0.034)	0.141 (0.045)	0.14 (0.034)	0.133 (0.048)	0.004	0.000	0.050	0.287	0.003	0.184	0.548
	Sphingomyelin (C26:1)	0.367 (0.107)	0.353 (0.131)	0.34 (0.079)	0.326 (0.076)	0.306 (0.092)	0.289 (0.12)	0.004	0.000	0.190	0.037	0.002	0.423	0.155
Sugar	Sugar	4883 (1165)	4649 (1181)	5543 (1528)	5151 (1045)	7616 (2872)	6990 (3195)	0.000	0.000	0.018	0.000	0.000	0.089	0.002

Supplementary Data 5 – Measured serum acylcarnitines, amino acids, biogenic amines, glycerophospholipids and sphingomyelins in participants with type 2 diabetes (T2D), impaired glucose tolerance (IGT) and participants without diabetes.

Bariatric Surgery – ^{1}H-NMR untargeted analysis

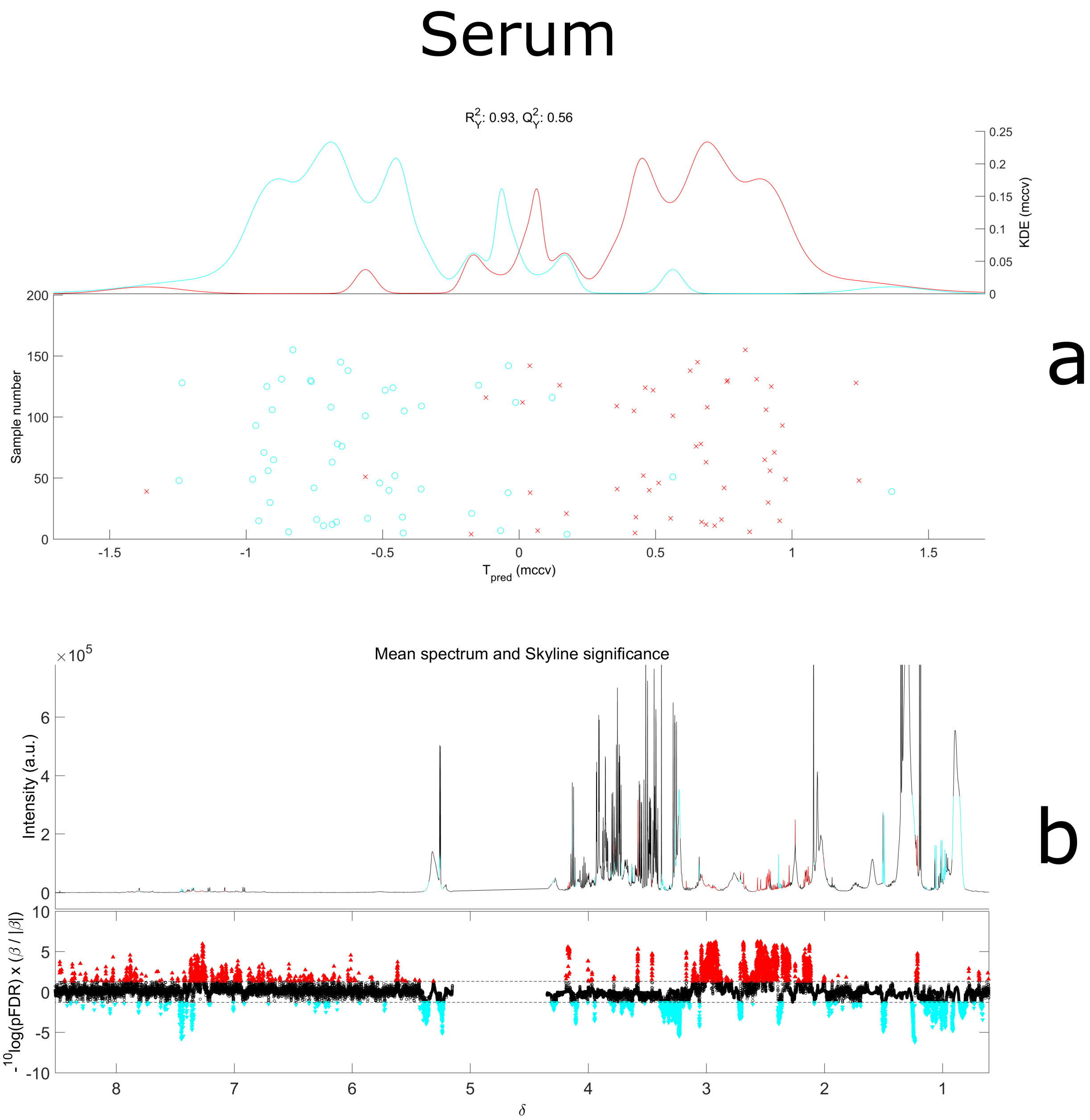

Supplementary Figure 4 – Longitudinal RM-MCCV-PLSDA model comparing ^{1}H-NMR analysis of participants before and after bariatric surgery in serum

a) RM-MCCV-PLSDA scores plots of ^{1}H-NMR analysis participant serum samples pre and 3-months post bariatric surgery (n=49). Models are comprised of 1 predictive and 1 orthogonal component. Model score: R^2Y 0.93, Q^2Y 0.56. b) Mean spectrum and Manhattan plot. Manhattan plot showing -log_{10}(pFDR) x sign of the variable regression coefficient for each variable within the RM-MCCV-PLSDA model. Dotted lines illustrate the pFDR significance cut off level (0.01) on the log_{10} scale. Spectra considered significant are highlighted in the Manhattan plot and mean spectrum. Red ^{1}H-NMR signals are significantly increased after surgery, blue ^{1}H-NMR signals are significantly decreased.

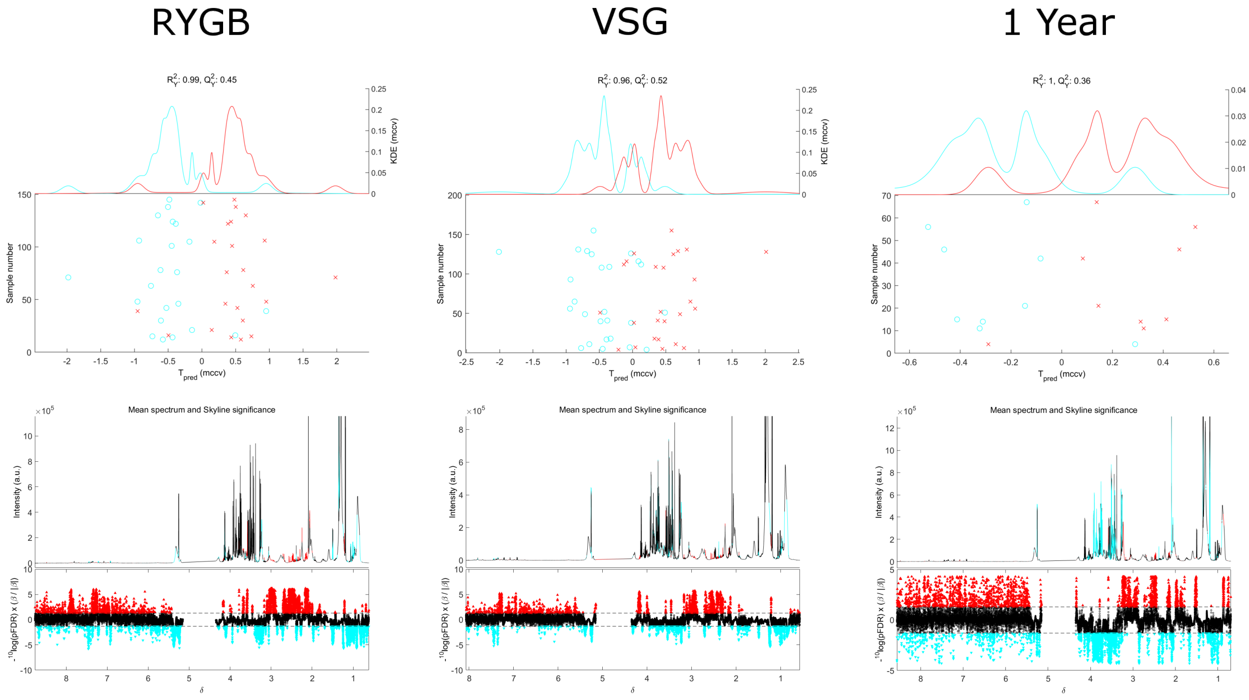

Supplementary Figure 5 – Serum ^{1}H-NMR RM-MCCV-PLSDA subgroup analysis

a) RM-MCCV-PLSDA scores plots of ^{1}H-NMR analysis comparing participant serum samples pre and 3-month post Roux-en-Y Gastric Bypass (RYGB) (n=23) or Vertical Sleeve Gastrectomy (VSG) surgery (n=26) and 1-year post bariatric surgery (n=9). Models are comprised of 1 predictive and 1 orthogonal component. Model scores: i) RYGB 3 months R^2Y 0.99, Q^2Y 0.45, ii) VSG 3 months R^2Y 0.96, Q^2Y 0.52, iii) 1 Year R^2Y 1.00, Q^2Y 0.36. b) Mean spectrum and Manhattan plots. Manhattan plots show -log_{10}(pFDR) x sign of the variable regression coefficient for each variable within the RM-MCCV-PLSDA model. Dotted lines illustrate the pFDR significance cut off level (0.01) on the log_{10} scale. Spectra considered significant are highlighted in the Manhattan plot and mean spectrum. Red ^{1}H-NMR signals are significantly increased after surgery, blue ^{1}H-NMR signals are significantly decreased.

Serum – Post Bariatric Surgery				
**Metabolite**	**^{1}H chemical shift δ (multiplicity[a])**	**Association**	**Model**	**Sig (UV[b])**
3-Hydroxybutyrate	1.22 (d), 2.32 (m), 2.42 (m), 4.17 (m)	↑	All	No
Glycine	3.58 (s)	↑	All	RYGB + VSG (Bioc)
Citrate	2.56 (ABx), 2.70 (ABx)	↑	All	All
Dimethylglycine	2.93 (s)	↑	All	NA
Glutamine	2.15 (m), 2.47 (m), 3.77 (t)*	↑	All	All
Formate	8.47 (s)	↑	All	3m + RYGB + 1Yr
Unknown A	7.88 (d)	↑	All	NA
Acetoacetate	3.46 (s), 2.30 (s)	↑	All	3m + RYGB + VSG
Acetone	2.25 (s)	↑	All	NA
Unknown B	3.17 (s) no stocsy/storm	↑	All	NA
Histidine	3.99 (dd), 7.08 (s), 7.80 (s)	↑	3m + RYGB + 1Yr	3m + RYGB
HDL	0.86 (m)	↑	1Yr	1Yr
Phosphocholine / choline	3.24 (s broad)	↑	1Yr	1Yr
Phenylalanine	7.35 (m), 7.39 (m), 7.45 (m), 4.00 (m)	↓	All	VSG
Glycerol from lipid	5.22	↓	All	3m + RYGB + VSG
Alanine	1.49 (d), 3.79 (q)*	↓	All	No
Lysine	[1.48 (m), 1.73 (m), 1.91 (m), 3.03 (t), 3.76 (t)]	↓	All	NA
VLDL/LDL	0.89 (t), 1.29 (m), 1.59 (m)	↓	All	3m + RYGB + VSG
Valine	1.01 (d), 1.06 (d), 2.28 (m), 3.63 (d)	↓	All	3m + RYGB + VSG
Isoleucine	0.96 (t), 1.03 (d), 1.48 (m), 1.26 (m)*, 1.98 (m)*, 3.68 (d)*	↓	All	3m + VSG
Leucine	0.98 (t), 1.71 (m), 3.73 (t)*	↓	All	3m + RYGB + VSG
Proline	2.01 (m)*, 2.07 (m)*, 2.37 (m), 3.36 (m), 3.43 (m)*, 4.13 (dd)	↓	All	RYGB + VSG (Bioc)
Pyruvate	2.39 (s) Storm – lactate	↓	All	VSG
Creatine	3.06 (s), 3.95 (s)	↓	All	NA
Glycerophosphocholine	3.23 (s broad)	↓	RYGB + VSG	3m + RYGB + VSG
Lactate	1.34 (d), 4.13 (q)	↓	RYGB + 1Yr	3m + RYGB (Quant)
Tyrosine	3.06 (Abx), 3.16 (Abx)*, 3.94 (Abx)*, 6.92 (d), 7.21 (d)	↓	RYGB	No
α Glucose	5.25 (d) + 3.23 (dd), 3.40 (m), 3.46 (m), 3.52 (dd), 3.73 (m), 3.82 (m), 3.88 (dd)	↓	1Yr	No
Glycoproteins	2.09 (s)	↓	1Yr	No

Supplementary Table 6 – Serum metabolites discriminating between participants pre- and post-surgery

Table shows serum metabolites increasing or decreasing after bariatric surgery in individual or all RM-MCCV-PLSDA models as indicated. [a]Multiplicity key is as follows: s=singlet, d=doublet, t=triplet, q=quartet, dd=doublet of doublets, m=multiplet. ^{1}H shifts marked by an asterisk (*) were not confirmed experimentally due to the absence of unambiguous resonance values and are taken from the Human Metabolome Database (HMDB). [b]Sig (UV) = metabolite significant on univariate analysis of quantified levels.

Urine

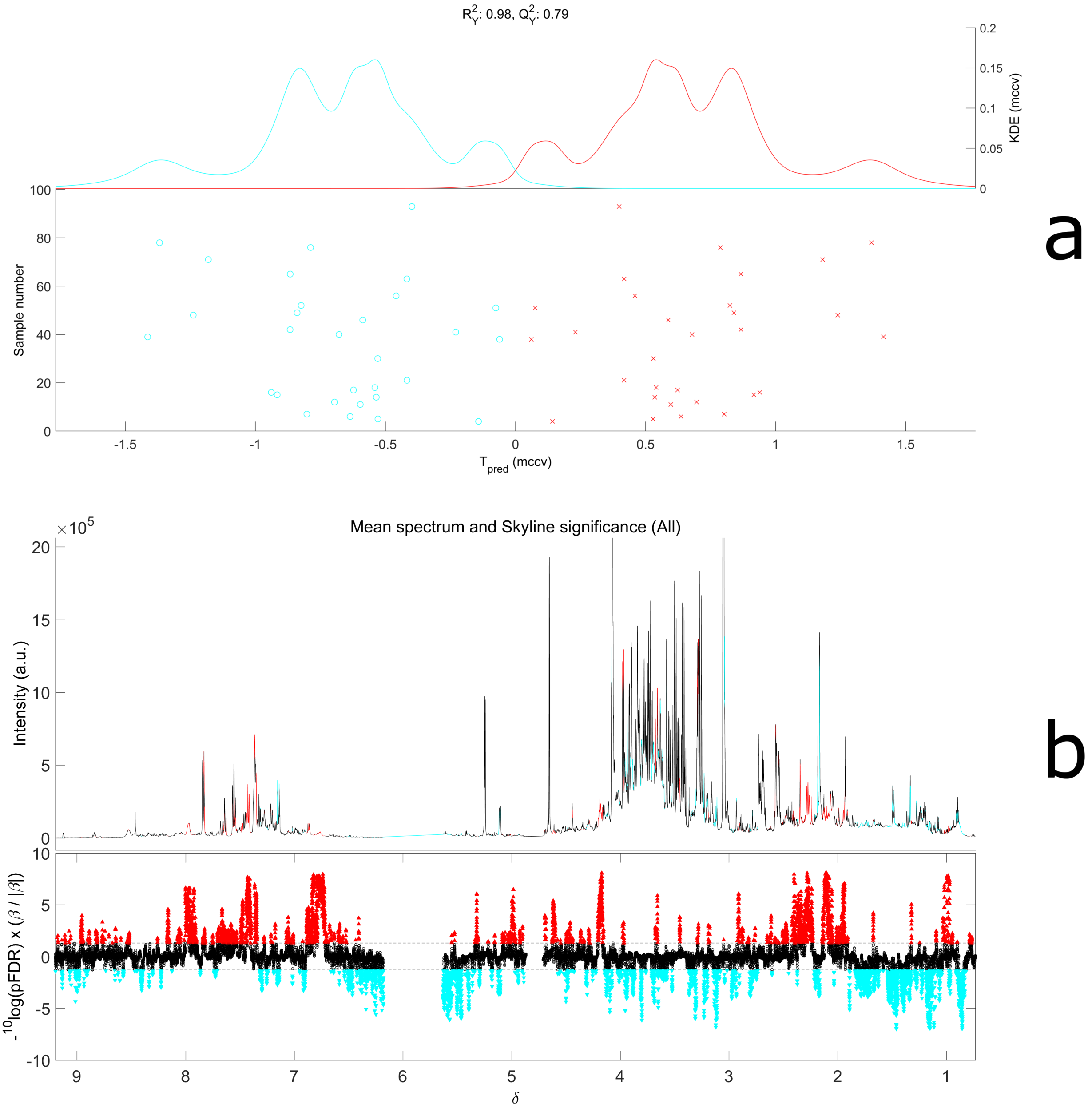

Supplementary Figure 6 – Longitudinal RM-MCCV-PLSDA model comparing ^{1}H-NMR analysis of participants before and after bariatric surgery in urine

a) RM-MCCV-PLSDA scores plots of ^{1}H-NMR analysis comparing participant 24-hour urine samples pre and 3-months post bariatric surgery (n=30). Models are comprised of 1 predictive and 1 orthogonal component. Model score: R^2Y 0.98, Q^2Y 0.79. b) Mean spectrum and Manhattan plot. Manhattan plot showing -log_{10}(pFDR) x sign of the variable regression coefficient for each variable within the RM-MCCV-PLSDA model. Dotted lines illustrate the pFDR significance cut off level (0.01) on the log_{10} scale. Spectra considered significant are highlighted in the Manhattan plot and mean spectrum. Red ^{1}H-NMR signals are significantly increased after surgery, blue ^{1}H-NMR signals are significantly decreased.

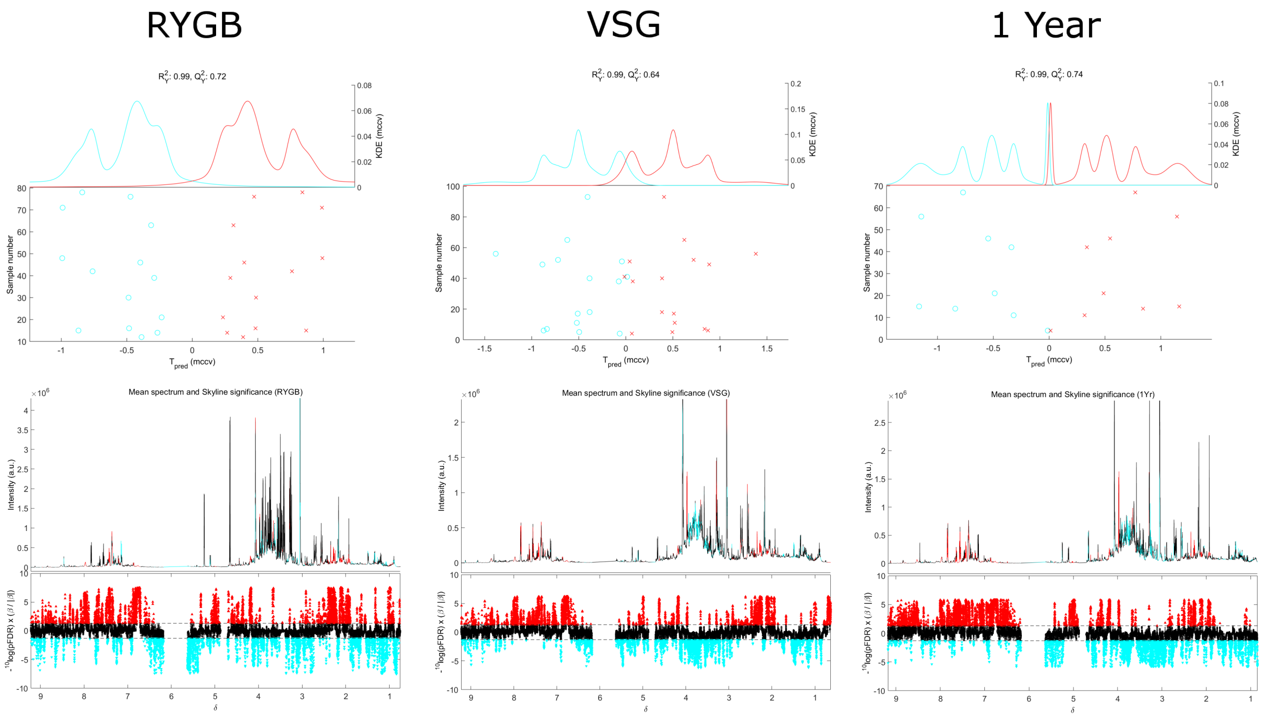

Supplementary Figure 7 – Urine ^{1}H-NMR RM-MCCV-PLSDA subgroup analysis

a) RM-MCCV-PLSDA scores plots of ^{1}H-NMR analysis comparing participant 24-hour urine samples pre and 3-month post Roux-en-Y Gastric Bypass (RYGB) (n=14) or Vertical Sleeve Gastrectomy (VSG) surgery (n=16) and 1-year post bariatric surgery (n=9). Models are comprised of 1 predictive and 1 orthogonal component. Model scores: i) RYGB 3 months R^2Y 0.99, Q^2Y 0.72, ii) VSG 3 months R^2Y 0.99, Q^2Y 0.64, iii) 1 Year R^2Y 0.99, Q^2Y 0.74. b) Mean spectrum and Manhattan plots. Manhattan plots show -$\log_{10}$(pFDR) x sign of the variable regression coefficient for each variable within the RM-MCCV-PLSDA model. Dotted lines illustrate the pFDR significance cut off level (0.01) on the $\log_{10}$ scale. Spectra considered significant are highlighted in the Manhattan plot and mean spectrum. Red ^{1}H-NMR signals are significantly increased after surgery, blue ^{1}H-NMR signals are significantly decreased.

Urine – Post Bariatric Surgery				
**Metabolite**	**^{1}H chemical shift δ (multiplicity[a])**	**Association**	**Model**	**Sig (UV[b])**
Phenylacetylglutamine (PAG)	2.11 (m), 2.27 (m), 3.67 (m), 4.19 (m), 7.36 (t), 7.43 (t)	↑	All	All
Unknown C	6.76 (m) 7.97 (d) (STORM – PAG, Glutamine related)	↑	All	NA
Hippurate	3.97 (d), 7.55 (t), 7.64 (t), 7.84 (d), 8.52 (m)	↑	All	All
4 Hydroxyphenylacetate	6.86 (m), 7.17 (m), 3.45 (s)	↑	All	All
TMAO	3.28 (s)	↑	All	3m + RYGB
Citrate	2.55~ (d), 2.69~ (d)	↑	All	No
4-cresyl sulfate	2.34 (s), 7.21 (d), 7.28 (d)	↑	All	All
Indoxyl sulfate	7.2 (m), 7.28 (m), 7.51 (d), 7.71 (d)	↑	All	All
Unknown D	1.32 (s)	↑	All	NA
2-aminobutyrate[ϕ]	0.98 (t), 1.90 (m)*, 3.72 (t)*	↑	All	NA
Butanone[ϕ]	1.02(t)	↑	All	NA
Levoglucosan	5.46 (s)	↓	All	NA
Acetaminophen (paracetamol) glucuronide	2.16 (s), 3.63 (m), 5.11(d), 7.14 (d), 7.36 (d)	↓	All	NA
Acetaminophen (paracetamol) sulfate	2.17 (s), 7.31 (d), 7.45 (d)	↓	All	NA
Creatine	3.04 (s), 3.94 (s)	↓	All	3m + RYGB + VSG
Lysine	1.48 (m), 1.73 (m), 1.91 (m), 3.03 (t), 3.76 (t)	↓	All	NA
Alanine	1.49 (d), 3.79 (q)*	↓	All	VSG
Unknown E	1.52 (m)	↓	All	NA
Ibuprofen[ϕ]	1.39 (d)	↓	All	NA
Ibuprofen Glucuronide[ϕ]	1.15 (d) (STORM 1.66 (s), 5.50 (m))	↓	All	NA
Isobutyrate	1.11 (d), 3.02 (m)*	↓	All	3m +RYGB
Lactate	1.34 (d), 4.13 (q)	↓	All	3m + RYGB + 1Yr
3 hydroxyisovaleric acid	1.27 (s), 2.37 (s)	↓	All	3m + RYGB + 1Yr
Leucine	0.97 (t), 1.71 (m)*, 3.73 (t)*	↓	3m + VSG + 1Yr	NA
Valine	1.00 (d), 1.05 (d), 2.28 (m)*, 3.63 (d)	↓	3m + RYGB + 1Yr	3m + 1Yr
Creatinine	3.05 (s), 4.06 (s)	↓	RYGB + 1Yr	1Yr
Formate	8.46 (s)	↓	RYGB	No
α Glucose	5.25 (d) + 3.23 (dd), 3.40 (m), 3.46 (m), 3.52 (dd), 3.73 (m), 3.82 (m), 3.88 (dd)	↓	1Yr	1Yr
β Glucose	4.66 (d) + 3.23 (dd), 3.40 (m), 3.46 (m), 3.52 (dd), 3.73 (m), 3.82 (m), 3.88 (dd)	↓	1Yr	No

Supplementary Table 7 – Urinary metabolites discriminating between participants pre- and post-surgery

Table shows urinary metabolites increasing or decreasing after bariatric surgery in individual or all RM-MCCV-PLSDA models as indicated. [a]Multiplicity key is as follows: s=singlet, d=doublet, t=triplet, q=quartet, dd=doublet of doublets, m=multiplet. ^{1}H shifts marked by an asterisk (*) were not confirmed experimentally due to the absence of unambiguous resonance values and are taken from the Human Metabolome Database (HMDB). $^{\phi}$ = tentative assignment. [b]Sig (UV) = metabolite significant on univariate analysis of quantified levels.

Faeces

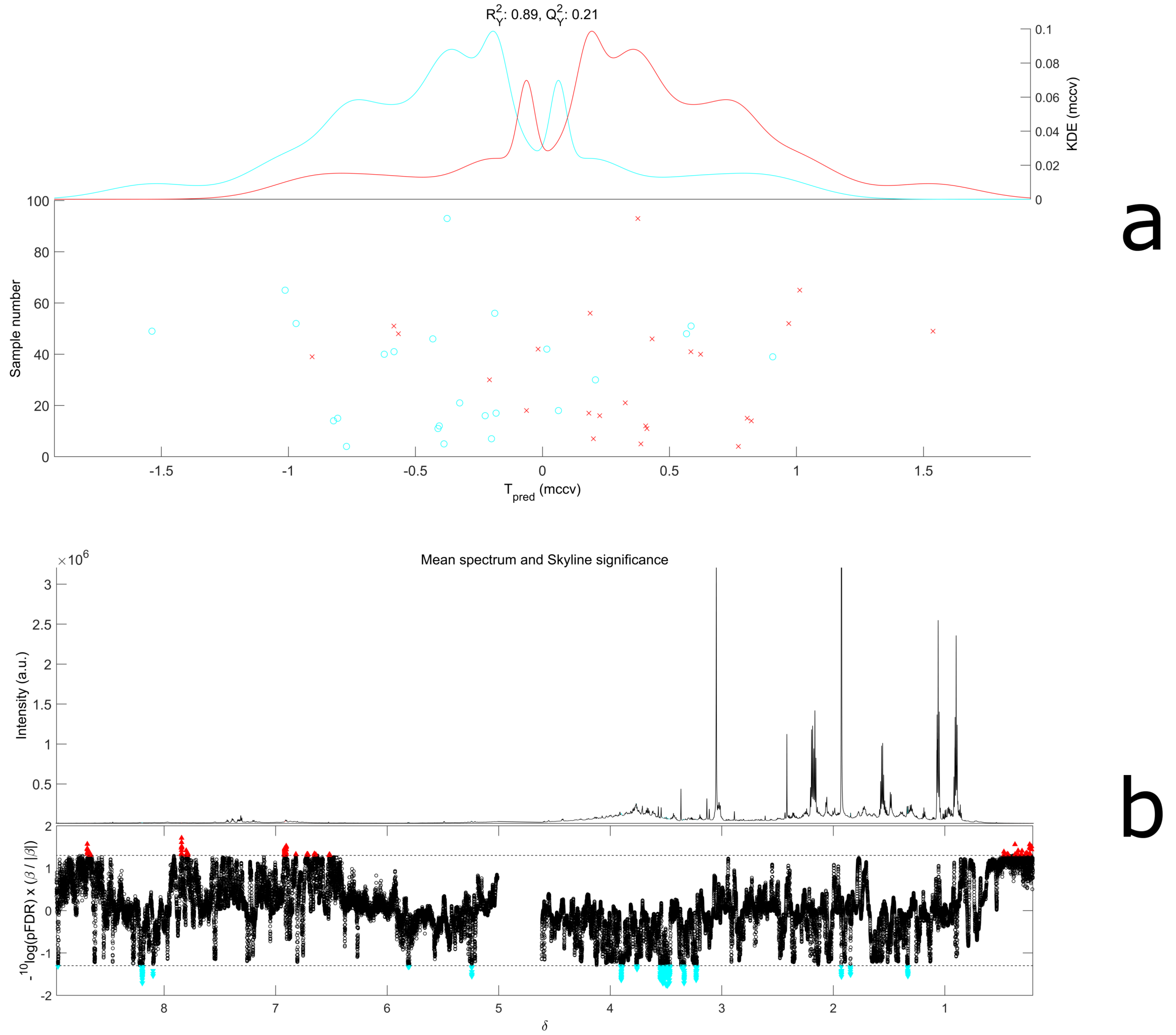

Supplementary Figure 8 – Longitudinal RM-MCCV-PLSDA model comparing ^{1}H-NMR analysis of participants before and after bariatric surgery in faeces

a) RM-MCCV-PLSDA scores plots of ^{1}H-NMR analysis participant faecal samples pre and 3-months post bariatric surgery (n=24). Models are comprised of 1 predictive and 1 orthogonal component. Model score: R^2Y 0.89, Q^2Y 0.21. b) Mean spectrum and Manhattan plot. Manhattan plot showing -log$_{10}$(pFDR) x sign of the variable regression coefficient for each variable within the RM-MCCV-PLSDA model. Dotted lines illustrate the pFDR significance cut off level (0.01) on the log$_{10}$ scale. Spectra considered significant are highlighted in the Manhattan plot and mean spectrum. Red ^{1}H-NMR signals are significantly increased after surgery, blue ^{1}H-NMR signals are significantly decreased.

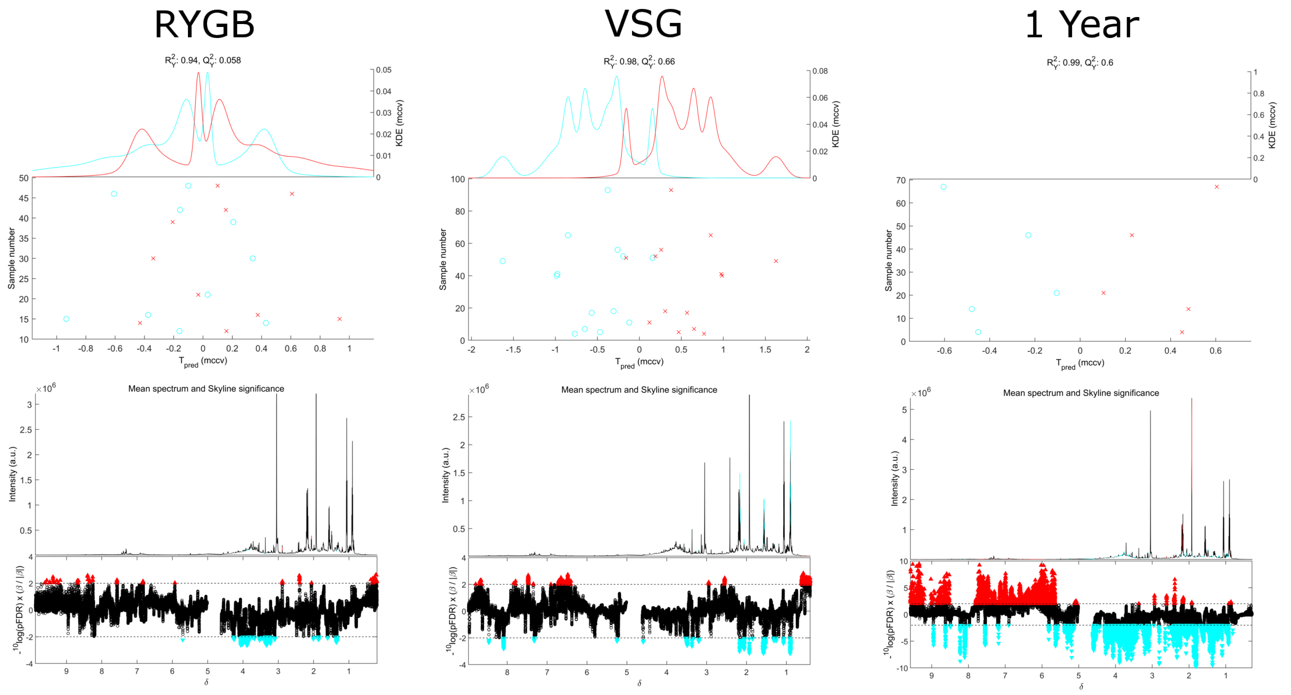

Supplementary Figure 9 – Faecal ^{1}H-NMR RM-MCCV-PLSDA subgroup analysis

a) RM-MCCV-PLSDA scores plots of ^{1}H-NMR analysis comparing participant faecal samples pre and 3-month post Roux-en-Y Gastric Bypass (RYGB) (n=10) or Vertical Sleeve Gastrectomy (VSG) surgery (n=14) and 1-year post bariatric surgery (n=5). Models are comprised of 1 predictive and 1 orthogonal component. Model scores: i) RYGB 3 months R^2Y 0.94, Q^2Y 0.06, ii) VSG 3 months R^2Y 0.98, Q^2Y 0.66, iii) 1 Year R^2Y 1.0, Q^2 0.60. b) Mean spectrum and Manhattan plots. Manhattan plots show -log_{10}(pFDR) x sign of the variable regression coefficient for each variable within the RM-MCCV-PLSDA model. Dotted lines illustrate the pFDR significance cut off level (0.01) on the log_{10} scale. Spectra considered significant are highlighted in the Manhattan plot and mean spectrum. Red ^{1}H-NMR signals are significantly increased after surgery, blue ^{1}H-NMR signals are significantly decreased.

Faeces – Post Bariatric Surgery				
**Metabolite**	**^{1}H chemical shift δ (multiplicity[a])**	**Association**	**Model**	**Sig (UV[b])**
Tyramine	6.92 (d), 7.22 (d), 2.93 (t), 3.24 (t)	↑	3m	NA
Beta-Alanine	2.56 (t), 3.18 (t)	↑	VSG	NA
Lactate	1.33 (d), 4.12 (q)	↓	3m + VSG	No
α Glucose	5.25 (d) + 3.23 (dd), 3.40 (m), 3.46 (m), 3.52 (dd), 3.73 (m), 3.82 (m), 3.88 (dd)	↓	3m + VSG	VSG
Isovalerate	0.91 (d), 1.96 (m), 2.06 (d)	↓	VSG	VSG
Formate	8.46 (s)	↓	VSG	3m + VSG
Trimethylamine	2.89 (s)	↓	VSG	VSG
Hypoxanthine	8.21 (d)	↓	3m	NA
Unknown A	8.09 (t)	↓	3m + VSG	NA
Acetate	1.93 (s)	↓	VSG	3m + VSG
Methanol	3.37 (s) – STORM ethanol + acetate	↓	3m + VSG	3m + VSG
Butyrate	0.90 (t), 1.56 (m), 2.16 (t)	↓	VSG	3m + VSG
Valerate	0.89 (t), 1.30 (m), 1.54 (m), 2.18 (t)	↓	VSG	3m + VSG
Phenylacetate	3.54 (s), 7.31 (m), 7.39 (m)	↓	VSG	VSG

Supplementary Table 8 – Faecal metabolites between participants pre- and post-surgery

Table shows faecal metabolites increasing or decreasing after bariatric surgery in individual or all RM-MCCV-PLSDA models as indicated. [a]Multiplicity key is as follows: s=singlet, d=doublet, t=triplet, q=quartet, dd=doublet of doublets, m=multiplet. ^{1}H shifts marked by an asterisk (*) were not confirmed experimentally due to the absence of unambiguous resonance values and are taken from the Human Metabolome Database (HMDB). [b]Sig (UV) = metabolite significant on univariate analysis of quantified levels.

Supplementary Data 6 – Change in metabolites measured with ^{1}H-NMR spectroscopy in faecal, urine and serum samples post bariatric surgery

		All 3 months (n=49)		Wilcoxon Rank Test		RYGB 3 months (n=23)		Wilcoxon Rank Test		VSG 3 months (n=26)		Wilcoxon Rank Test		1Yr (n=9)		Wilcoxon Rank Test		
	Metabolite (ppm)	Mean change RI (SD)	Median change RI (IQR)	p	pFDR	Mean change RI (SD)	Median change RI (IQR)	p	pFDR	Mean change RI (SD)	Median change RI (IQR)	p	pFDR	Mean change RI (SD)	Median change RI (IQR)	p	pFDR	
Serum	HDL CH3 (0.86)	4747 (51646)	12570 (52340)	0.289	0.331	9166 (48788)	12570 (46870)	0.181	0.241	838 (54706)	12555 (88176)	0.770	0.804	74578 (51981)	81365 (88240)	0.004	0.047	Serum
	VLDL CH3 (0.89)	-138876 (329834)	-62503 (206337)	0.001	0.001	-166415 (346051)	-106324 (174715)	0.001	0.004	-114515 (319659)	-42762 (263666)	0.091	0.156	-107983 (215984)	-112350 (272196)	0.129	0.309	
	Leucine (0.98)	-24998 (51075)	-25943 (54187)	0.001	0.002	-26074 (58027)	-28919 (44267)	0.031	0.062	-24046 (45206)	-22625 (62476)	0.018	0.038	10518 (69952)	208 (89089)	0.820	0.856	
	Isoleucine (1.03)	-10660 (29299)	-8605 (27356)	0.002	0.004	-7843 (36101)	-8342 (17115)	0.068	0.117	-13152 (22080)	-11747 (37066)	0.012	0.038	5459 (35153)	5549 (43394)	0.652	0.839	
	Valine (1.06)	-24179 (55897)	-24996 (57991)	0.001	0.003	-28757 (67653)	-38459 (55122)	0.029	0.062	-20130 (43989)	-20172 (56360)	0.025	0.049	-7263 (64916)	-3215 (123296)	0.652	0.839	
	3Hydroxybutyrate (1.19)	-89225 (1263602)	2722 (1236097)	0.754	0.823	172741 (963163)	219048 (957025)	0.429	0.468	-320963 (1459554)	-116406 (1473013)	0.304	0.361	-446344 (1232404)	-235923 (1102833)	0.250	0.400	
	CH2 Lipid (1.30)	-592539 (1136197)	-340355 (670245)	0.000	0.000	-595293 (1201551)	-328372 (625007)	0.001	0.005	-590103 (1099145)	-411124 (740496)	0.003	0.019	-411250 (827931)	-144824 (847346)	0.129	0.309	
	CH2CH2COOC Lipid (1.59)	-45461 (90116)	-29747 (55937)	0.000	0.000	-44782 (90192)	-29747 (41725)	0.002	0.005	-46062 (91830)	-34371 (59089)	0.005	0.019	-34268 (67700)	-11084 (68643)	0.164	0.328	
	Alanine (1.50)	-13031 (67644)	-16842 (85832)	0.140	0.168	-8719 (67807)	-24983 (89859)	0.274	0.344	-16846 (68609)	-12210 (76364)	0.292	0.361	24718 (108686)	30942 (99563)	0.734	0.839	
	Acetate (1.93)	10918 (21874)	8701 (24062)	0.002	0.003	11881 (24636)	2983 (21808)	0.059	0.110	10066 (19568)	9117 (27124)	0.015	0.038	-4402 (31653)	-2506 (34175)	0.910	0.910	
	N-acetylglycoproteins (2.06)	-1836 (37305)	-530 (47671)	0.792	0.827	9630 (36189)	3874 (48409)	0.301	0.344	-11978 (35939)	-10479 (48740)	0.124	0.199	-36382 (52304)	-59242 (70458)	0.074	0.297	
	Pyruvate (2.39)	-17137 (56897)	-18683 (75129)	0.064	0.095	-11580 (66860)	-17897 (106364)	0.465	0.486	-22052 (47203)	-22435 (63322)	0.049	0.091	-61746 (80173)	-81040 (125554)	0.098	0.309	
	Glutamine (2.47)	14166 (16009)	14816 (25472)	0.000	0.000	19160 (13018)	16451 (17439)	0.000	0.001	9749 (17312)	5995 (26286)	0.016	0.038	18310 (21197)	12860 (29770)	0.039	0.234	
	Citrate (2.57)	20062 (17873)	21374 (23102)	0.000	0.000	23059 (18447)	22604 (13598)	0.000	0.001	17412 (17273)	16124 (27137)	0.000	0.006	5395 (8999)	3514 (8308)	0.164	0.328	
	Glycerophosphocholine (3.23)	-41358 (54635)	-29158 (55716)	0.000	0.000	-39517 (48384)	-25823 (44142)	0.000	0.003	-42987 (60541)	-33254 (75160)	0.002	0.015	26030 (57648)	26361 (56206)	0.203	0.348	
	Phosphocholine (3.24)	9969 (61259)	10784 (70698)	0.096	0.121	9365 (65617)	29930 (73246)	0.153	0.216	10503 (58442)	8468 (63750)	0.316	0.361	123174 (62357)	119014 (60598)	0.004	0.047	
	Acetoacetate (3.26)	27307 (44737)	12461 (59751)	0.000	0.000	31687 (53524)	17444 (53378)	0.010	0.026	23432 (35894)	9225 (53364)	0.014	0.038	-4643 (18501)	-3603 (19168)	0.570	0.839	
	Lactate (4.13)	-13079 (126846)	11625 (161170)	0.996	0.996	-15735 (154726)	31093 (193281)	0.903	0.903	-10729 (99062)	-2093 (132118)	0.829	0.829	-45255 (170501)	4731 (270131)	0.734	0.839	
	Glycerol of lipid (5.22)	-9607 (17657)	-6175 (10794)	0.000	0.000	-10087 (17610)	-6175 (10144)	0.001	0.005	-9182 (18036)	-7013 (12525)	0.004	0.019	-6696 (13922)	-2216 (12726)	0.203	0.348	
	a Glucose (5.25)	-46138 (291059)	-18136 (199028)	0.081	0.108	-46807 (392439)	-33395 (262042)	0.136	0.204	-45546 (164697)	-16523 (95129)	0.280	0.361	-142593 (203346)	-171887 (269992)	0.074	0.297	
	Tyrosine (6.92)	-1720 (6622)	-1487 (8510)	0.068	0.096	-1733 (6981)	-1164 (6350)	0.287	0.344	-1709 (6427)	-3449 (8562)	0.166	0.235	1483 (10135)	-1175 (14887)	0.820	0.856	
	Histidine (7.08)	914 (3178)	599 (3669)	0.021	0.034	1534 (3548)	1470 (2760)	0.012	0.028	366 (2765)	320 (3757)	0.485	0.529	2880 (4922)	2756 (4423)	0.129	0.309	
	Phenylalanine (7.44)	-2085 (3331)	-1927 (4596)	0.000	0.001	-1805 (3888)	-1564 (5344)	0.078	0.124	-2332 (2805)	-2339 (3698)	0.001	0.009	796 (5162)	-1096 (7410)	0.734	0.839	
	Formate (8.48)	1973 (3016)	1914 (3858)	0.000	0.000	3391 (3165)	3158 (3134)	0.000	0.001	720 (2273)	574 (2803)	0.151	0.227	3513 (3722)	1867 (5372)	0.020	0.156	

		All 3 months (n=30)		Wilcoxon Rank Test		RYGB 3 months (n=14)		Wilcoxon Rank Test		VSG 3 months (n=16)		Wilcoxon Rank Test		1Yr (n=9)		Wilcoxon Rank Test		
	Metabolite (ppm)	Mean change RI (SD)	Median change RI (IQR)	p	pFDR	Mean change RI (SD)	Median change RI (IQR)	p	pFDR	Mean change RI (SD)	Median change RI (IQR)	p	pFDR	Mean change RI (SD)	Median change RI (IQR)	p	pFDR	
Urine	2-Aminobutyrate (0.98)	57931 (59505)	50316 (66038)	0.000	0.000	65390 (40363)	62599 (60091)	0.000	0.002	51404 (73051)	45119 (60160)	0.008	0.044	-5975 (27461)	3938 (35354)	0.820	0.963	Urine
	Valine (1.0)	-10498 (20050)	-8411 (31024)	0.017	0.028	-12078 (22797)	-16288 (38295)	0.091	0.153	-9116 (17957)	-2983 (28585)	0.121	0.218	-18481 (23112)	-17989 (19134)	0.039	0.081	
	Isobutyrate (1.11)	-30363 (66898)	-34178 (89671)	0.015	0.027	-42402 (45328)	-55060 (75064)	0.013	0.033	-19829 (81351)	-20993 (100142)	0.278	0.300	-40778 (63607)	-18292 (93214)	0.250	0.397	
	3Hydroxyisovalerate (1.27)	-125846 (189676)	-84744 (167380)	0.001	0.002	-146192 (158791)	-137730 (191006)	0.002	0.013	-108043 (216745)	-65728 (134329)	0.056	0.137	-178622 (192904)	-172922 (106946)	0.012	0.045	
	Lactate (1.34)	-532835 (1162407)	-86707 (460471)	0.006	0.012	-542717 (778136)	-157851 (733809)	0.009	0.029	-524188 (1444773)	-45091 (299054)	0.278	0.300	-561390 (835851)	-345589 (402180)	0.027	0.067	
	Alanine (1.49)	-169814 (241140)	-144293 (305171)	0.001	0.002	-118708 (306550)	-130657 (205591)	0.268	0.380	-214532 (162502)	-170727 (270039)	0.001	0.005	-179593 (367053)	-38310 (136311)	0.301	0.451	
	Acetate (1.93)	-1429664 (5185382)	-56349 (345058)	0.614	0.638	-2522042 (7374846)	79575 (373400)	0.761	0.822	-473833 (1661026)	-78519 (328545)	0.278	0.300	-2994989 (10130320)	64867 (383424)	1.000	1.000	
	4-Cresyl sulfate(2.34)	556780 (637700)	423684 (878855)	0.000	0.000	534844 (805201)	301763 (630562)	0.011	0.029	575973 (472722)	589851 (849078)	0.000	0.005	479285 (314113)	547481 (437023)	0.004	0.042	
	Citrate (2.55)	485708 (1744117)	452695 (1719639)	0.052	0.082	294996 (1641968)	660709 (1275387)	0.173	0.274	652582 (1865706)	369826 (2055500)	0.134	0.226	-628438 (1177087)	-210479 (2046498)	0.359	0.485	
	Trimethylamine (2.87)	6937 (55223)	23241 (71370)	0.192	0.235	3245 (63967)	20144 (72710)	0.583	0.716	10167 (48227)	26157 (69668)	0.196	0.279	-29822 (60669)	-29585 (46777)	0.164	0.316	
	Dimethylglycine (2.93)	-122391 (207946)	-109451 (271824)	0.004	0.008	-104075 (223170)	-150976 (320673)	0.068	0.130	-138417 (199623)	-100836 (206799)	0.023	0.062	-288476 (276896)	-147000 (152984)	0.004	0.042	
	Proline Betaine (3.11)	-153600 (271866)	-77589 (305767)	0.005	0.010	-194915 (270041)	-105062 (260249)	0.007	0.029	-117448 (276963)	-32140 (219160)	0.179	0.268	91134 (670923)	12507 (599759)	1.000	1.000	
	Carnitine (3.23)	-432395 (748117)	-218136 (484939)	0.000	0.002	-253510 (344408)	-214029 (305019)	0.017	0.037	-588919 (960857)	-345434 (641425)	0.004	0.030	-262199 (282591)	-131959 (378872)	0.020	0.059	
	TMAO (3.28)	5112980 (8557158)	2839419 (6580864)	0.002	0.005	8446986 (9699192)	5182282 (8802966)	0.000	0.003	2195724 (6370623)	2250279 (8604393)	0.234	0.300	3686718 (15901362)	1705415 (16203749)	0.570	0.733	
	Glycine (3.57)	-684280 (2144372)	-238505 (1576729)	0.178	0.229	-692282 (2628105)	60575 (1589755)	0.952	0.988	-677278 (1704108)	-469969 (1477852)	0.098	0.204	-1047316 (3397050)	117367 (857945)	0.910	1.000	
	Creatine (3.94)	-1224238 (2438988)	-236401 (1029782)	0.001	0.002	-1861200 (3329442)	-381593 (2169218)	0.011	0.029	-666896 (1087923)	-220246 (913811)	0.015	0.051	-336759 (665130)	-31742 (715263)	0.359	0.485	
	Hippurate (3.97)	556305 (982731)	437293 (827655)	0.001	0.003	568479 (1086334)	448731 (857530)	0.025	0.051	545653 (918751)	381324 (795095)	0.020	0.060	1252775 (1276756)	973374 (908998)	0.008	0.042	
	Creatinine (4.06)	134548 (1790204)	342308 (2317727)	0.572	0.617	-388888 (1640436)	-341333 (2120282)	0.542	0.696	592555 (1839649)	624624 (2741390)	0.215	0.290	-3170684 (2865282)	-3316391 (2608061)	0.012	0.045	
	Methylnicotinate (4.44)	32564 (350855)	24259 (279860)	0.688	0.688	75141 (393705)	60789 (343283)	0.670	0.786	-4691 (316990)	-13565 (211171)	0.959	0.959	278736 (309649)	301170 (445668)	0.027	0.067	
	B Glucose (4.65)	-3718904 (16345394)	-13994 (73700)	0.060	0.085	-7028846 (23830350)	-30933 (1384624)	0.091	0.153	-822704 (2278676)	-7553 (78408)	0.326	0.338	-1441701 (3108689)	4585 (60778)	1.000	1.000	
	a Glucose (5.25)	-1721623 (7519672)	-14122 (133309)	0.057	0.085	-3223703 (10964331)	-23912 (680557)	0.217	0.325	-407303 (1088637)	-11136 (86703)	0.148	0.235	-740178 (1500055)	-15313 (122708)	0.039	0.081	
	4-Hydroxyphenylacetate (6.87)	94472 (153066)	45326 (134911)	0.000	0.002	150592 (203385)	116728 (139056)	0.002	0.012	45367 (62728)	35852 (67459)	0.015	0.051	68055 (73667)	53625 (34222)	0.020	0.059	
	PAG (7.43)	358543 (271370)	358328 (386103)	0.000	0.000	483260 (292639)	531895 (507103)	0.000	0.002	249417 (202324)	198955 (284287)	0.001	0.005	359336 (270772)	359143 (417432)	0.008	0.042	
	Indoxyl sulfate (7.70)	26903 (33505)	21901 (49616)	0.000	0.002	34744 (40136)	43536 (52980)	0.009	0.029	20042 (25820)	20288 (32651)	0.010	0.044	32075 (27287)	26548 (35379)	0.008	0.042	
	Formate (8.46)	-240518 (912205)	8862 (203823)	0.254	0.285	-390827 (1273655)	14273 (215218)	0.715	0.804	-108998 (404127)	-17017 (187631)	0.255	0.300	-460617 (1594438)	20830 (198044)	0.652	0.801	
	Methylnicotinamide (9.29)	1342 (5659)	1496 (5578)	0.213	0.250	199 (5777)	342 (9881)	1.000	1.000	2342 (5541)	1569 (3276)	0.121	0.218	-1498 (6530)	-2341 (8344)	0.250	0.397	

		All 3 months (n=24)		Wilcoxon Rank Test		RYGB 3 months (n=10)		Wilcoxon Rank Test		VSG 3 months (n=14)		Wilcoxon Rank Test		1Yr (n=5)		Wilcoxon Rank Test		
	Metabolite (ppm)	Mean change RI (SD)	Median change RI (IQR)	p	pFDR	Mean change RI (SD)	Median change RI (IQR)	p	pFDR	Mean change RI (SD)	Median change RI (IQR)	p	pFDR	Mean change RI (SD)	Median change RI (IQR)	p	pFDR	
Faeces	2-Methylbutyrate (0.86)	10691 (219592)	-18432 (139006)	0.4576	0.5882	147568 (254659)	21216 (294130)	0.275	0.999	-87078 (125341)	-69893 (155943)	0.025	0.049	80312 (169976)	115949 (234364)	0.438	0.758	Faeces
	Butyrate (0.90)	-1382587 (2744273)	-1074049 (2270431)	0.0119	0.0443	79826 (2874171)	-470423 (3060961)	1.000	1.000	-2427167 (2187437)	-1586706 (2599692)	0.000	0.005	-838234 (2498664)	345026 (4239846)	0.813	0.880	
	Isovalerate (0.92)	57120 (637418)	-70338 (274027)	0.2652	0.5303	487149 (751965)	27353 (1004799)	0.232	0.999	-250043 (288996)	-182375 (281512)	0.002	0.010	363211 (609145)	441961 (1083227)	0.313	0.580	
	Leucine (0.96)	-5086 (146339)	-225 (138259)	0.8415	0.8415	-4177 (152167)	14673 (86667)	0.625	0.999	-5736 (147840)	-46533 (128796)	0.626	0.678	-46423 (67007)	-82881 (78025)	0.188	0.580	
	Valine (0.99)	-13162 (189388)	-5062 (146055)	0.5485	0.6482	-20020 (157024)	7468 (101041)	0.846	0.999	-8263 (215236)	-44414 (178697)	0.463	0.547	-66158 (89128)	-120301 (127959)	0.313	0.580	
	Isoleucine (1.01)	-17534 (98362)	-7765 (97489)	0.4405	0.5882	-18329 (109245)	1708 (66193)	1.000	1.000	-16965 (94097)	-36192 (104791)	0.358	0.465	-38231 (62346)	-71721 (95837)	0.313	0.580	
	Propionate (1.06)	-726788 (2748683)	-1234903 (2271528)	0.1161	0.2515	243069 (2623911)	-492391 (4339479)	0.846	0.999	-1419543 (2712958)	-1468209 (1857391)	0.017	0.036	57701 (1499127)	656706 (1818251)	0.813	0.880	
	Ethanol (1.18)	-69797 (294308)	-45602 (98386)	0.0919	0.2171	36727 (300806)	1709 (131356)	0.770	0.999	-145885 (275025)	-69863 (68793)	0.013	0.035	-129725 (230618)	-35454 (85271)	0.313	0.580	
	Lactate (1.34)	-112948 (274658)	-39487 (162346)	0.052	0.1353	-124956 (326452)	-17511 (134138)	0.375	0.999	-104371 (243918)	-49231 (176749)	0.119	0.182	-276692 (449628)	-57579 (213512)	0.125	0.580	
	Lysine (1.72)	55485 (253208)	-4838 (204494)	0.5872	0.6638	173740 (301838)	116500 (364814)	0.232	0.999	-28983 (178657)	-9562 (135529)	0.715	0.743	-10822 (151809)	13724 (122346)	1.000	1.000	
	Putrescine (1.77)	84987 (208643)	-3438 (126327)	0.3173	0.55	115053 (207132)	32655 (146904)	0.131	0.999	63512 (214764)	-11502 (118618)	0.952	0.952	2496 (107487)	-45687 (12521)	0.625	0.813	
	Acetate (1.93)	-9789348 (13190905)	-9833632 (15938392)	0.0033	0.0211	-2952627 (11436444)	-4274453 (15136558)	0.557	0.999	-14672720 (12476437)	-13234711 (15564364)	0.001	0.010	-4496789 (8355608)	-2756839 (3833130)	0.313	0.580	
	Valerate (2.17)	-943740 (1798473)	-741592 (1223796)	0.0093	0.0443	17124 (1914649)	-483276 (1752552)	0.922	1.000	-1630072 (1402562)	-1115299 (1508052)	0.000	0.005	-552925 (1659872)	260251 (2567481)	0.813	0.880	
	Succinate (2.42)	1555596 (7716796)	-6959 (89990)	0.4751	0.5882	-9689 (505822)	8802 (75327)	1.000	1.000	2673656 (10096918)	-16135 (108943)	0.358	0.465	-217498 (214380)	-119560 (258957)	0.063	0.406	
	Methylamine (2.61)	-9694 (66223)	-4272 (68449)	0.3037	0.55	3044 (63886)	16794 (32909)	0.492	0.999	-18793 (68697)	-26676 (63244)	0.091	0.147	25684 (40177)	32136 (50711)	0.313	0.580	
	Trimethylamine (2.88)	61617 (253036)	-18400 (112516)	0.7751	0.8061	224615 (312741)	57803 (494726)	0.193	0.999	-54809 (102623)	-36469 (87379)	0.042	0.073	16533 (187513)	83692 (11552)	0.625	0.813	
	Dimethylbiguanide (3.05)	-171993 (4700327)	-55214 (1246945)	0.3914	0.5653	831681 (6183347)	-103298 (4615325)	0.846	0.999	-888902 (3360033)	-26245 (255453)	0.542	0.612	-792688 (2438061)	-386567 (1725535)	0.625	0.813	
	Methanol (3.37)	-291114 (468442)	-221316 (309253)	0.0011	0.0162	-173568 (297162)	-243663 (302647)	0.105	0.999	-375075 (555772)	-197790 (334211)	0.003	0.011	45059 (264892)	40198 (201398)	1.000	1.000	
	Glycine (3.57)	-107861 (285012)	-58342 (153134)	0.0321	0.0928	-34372 (181813)	15127 (91261)	0.846	0.999	-160354 (337210)	-89354 (71388)	0.013	0.035	-106905 (93425)	-70493 (147086)	0.063	0.406	
	a Glucose (5.24)	-14813 (21666)	-10084 (17352)	0.0012	0.0162	-10729 (20840)	-4237 (18855)	0.105	0.999	-17730 (22536)	-13004 (13717)	0.002	0.010	-29371 (18187)	-21520 (17980)	0.063	0.406	
	Uracil (5.81)	-8195 (12847)	-5278 (9330)	0.0025	0.0211	-2845 (7489)	-2951 (6928)	0.375	0.999	-12017 (14678)	-7379 (10592)	0.002	0.010	-8148 (6321)	-8000 (11182)	0.063	0.406	
	Fumarate (6.52)	-201 (24885)	-1740 (13060)	0.3914	0.5653	-2466 (17298)	1134 (10940)	0.846	0.999	1417 (29693)	-3773 (10134)	0.268	0.387	-8076 (19693)	-3383 (21619)	0.625	0.813	
	Tyrosine (6.91)	-1330 (35988)	-1750 (34103)	0.7103	0.7695	4906 (37998)	7093 (33514)	0.695	0.999	-5785 (35222)	-13192 (38067)	0.426	0.528	-3787 (5586)	-5833 (2025)	0.313	0.580	
	Phenylacetate (7.31)	-2135 (97166)	-22242 (80000)	0.3606	0.5653	46825 (117744)	-3064 (145354)	0.625	0.999	-37108 (62806)	-43537 (59100)	0.042	0.073	49117 (61448)	77638 (106831)	0.313	0.580	
	Formate (8.46)	-5977 (11124)	-5658 (9598)	0.0278	0.0904	-3698 (12289)	-3741 (11557)	0.695	0.999	-7606 (10369)	-6807 (9123)	0.013	0.035	-12102 (19966)	-87 (31382)	0.625	0.813	

Supplementary Data 6 – Univariate analysis of changes post Roux-en-Y Gastric Bypass (RYGB) and Vertical Sleeve Gastrectomy (VSG) procedures in semi-quantified metabolites (relative concentrations) measured from serum, urine and faecal water ^{1}H-NMR experiments.

Bariatric Surgery – Targeted analyses

Supplementary Data 7 – Change in bile acids post bariatric surgery in faecal and serum samples

Faecal

	All 3 months (n=26 paired)		Wilcoxon Rank Test		RYGB 3 months (n=11 paired)		Wilcoxon Rank Test		VSG 3 months (n=14 paired)		Wilcoxon Rank Test		1Yr (n=5 paired)	
	Mean change μM (SD)	Median change μM (IQR)	p	pFDR	Mean change μM (SD)	Median change μM (IQR)	p	pFDR	Mean change μM (SD)	Median change μM (IQR)	p	pFDR	Mean change μM (SD)	Median change μM (IQR)
All	-2208062 (1804799)	-2052188 (1729565)	0.000	0.000	-2425865 (1365628)	-2067888 (948536)	0.001	0.016	-2072497 (2179557)	-1942616 (2858310)	0.003	0.025	-3590314 (1783126)	-2895743 (1903243)
All [1]	-363131 (778416)	-21560 (312500)	0.003	0.015	-98702 (273657)	-18060 (41810)	0.083	0.296	-608471 (981006)	-39260 (1160525)	0.004	0.029	-447648 (756717)	-34660 (384780)
All [2]	-1259752 (1227716)	-1335710 (1617700)	0.000	0.000	-1613247 (895033)	-1655260 (777110)	0.001	0.016	-950353 (1434739)	-715810 (1937370)	0.007	0.037	-2013564 (1086978)	-2082240 (780660)
All [1c]	-5353 (191870)	-5999 (29263)	0.013	0.054	-20094 (53914)	-2893 (22958)	0.175	0.416	2229 (260909)	-12223 (67629)	0.013	0.048	-53590 (70270)	-5178 (120295)
All [2c]	-2282 (3704)	-1214 (1952)	0.000	0.000	-3315 (5359)	-998 (2187)	0.011	0.077	-1544 (1651)	-1378 (2139)	0.001	0.020	-6595 (6768)	-3185 (11703)
All Others	-577544 (832962)	-301485 (221583)	0.000	0.000	-690507 (1135282)	-295125 (235350)	0.001	0.016	-514358 (563818)	-324619 (267888)	0.000	0.006	-1068918 (1668377)	-341400 (119558)
[1] Cholic acid (CA)	-169215 (551364)	-3660 (5650)	0.031	0.098	50529 (127923)	-1020 (5290)	0.831	0.966	-367413 (692122)	-5950 (465550)	0.005	0.033	-256668 (542046)	-4760 (57160)
[1] Chenodeoxycholic acid (CDCA)	-48845 (320265)	-10300 (52760)	0.173	0.288	38684 (235608)	-6240 (31250)	0.520	0.722	-133230 (369761)	-24910 (246045)	0.119	0.229	-148772 (181881)	-28760 (314080)
CA:CDCA ratio	-0.27 (0.72)	-0.079 (0.528)	0.099	0.205	-0.019 (0.576)	-0.028 (0.202)	0.898	0.966	-0.54 (0.728)	-0.415 (0.705)	0.009	0.039	-0.158 (0.776)	-0.198 (0.89)
[1c] Glycocholic Acid (GCA)	-932 (2268)	-170 (949)	0.035	0.104	-544 (1594)	-155 (829)	0.123	0.342	-1370 (2709)	-306 (918)	0.065	0.154	-1241 (2572)	-218 (470)
[1c] Taurocholic acid (TCA)	-198.3 (1187.3)	-17.5 (138.8)	0.067	0.167	-19.1 (163.5)	-7.5 (78.8)	0.320	0.517	-371.6 (1617.1)	-47.5 (388.1)	0.057	0.145	-119.5 (162.7)	-37.5 (205)
[1c] Glycochenodeoxycholic acid (GCDCA)	-2078 (6862)	-181 (1646)	0.020	0.068	-3433 (10379)	-235 (956)	0.083	0.296	-1258 (2122)	-306 (1888)	0.068	0.154	-7624 (16412)	-473 (215)
[1c] Taurochenodeoxycholic acid (TCDCA)	-469 (190084)	-4534 (23322)	0.019	0.068	-14866 (47299)	-2595 (19381)	0.278	0.492	7108 (259453)	-11436 (64722)	0.013	0.048	-43902 (59411)	-3578 (89770)
G:T ratio	0.012 (0.114)	0.035 (0.179)	0.532	0.618	0.003 (0.131)	0.049 (0.205)	0.966	0.966	0.025 (0.105)	0.031 (0.137)	0.358	0.526	-0.007 (0.053)	0.005 (0.052)
G:T-CA ratio	-2.72 (10.32)	-1.11 (9.24)	0.291	0.394	-6.07 (10.8)	-6.11 (9.9)	0.102	0.299	0.06 (9.85)	1.16 (5.53)	0.502	0.643	-1.57 (5.5)	-0.65 (5.9)
G:T-CDCA ratio	0.02 (0.088)	0.031 (0.103)	0.099	0.205	0.008 (0.124)	0.042 (0.141)	0.700	0.854	0.033 (0.047)	0.031 (0.08)	0.020	0.068	-0.041 (0.088)	-0.048 (0.099)
Conjugated CA:CA ratio	0.208 (1.266)	0.011 (0.16)	0.237	0.339	0.453 (1.363)	-0.003 (0.139)	0.700	0.854	0.036 (1.251)	0.079 (0.21)	0.035	0.110	-0.03 (0.188)	-0.053 (0.209)
Conjugated CDCA:CDCA ratio	-0.134 (1.024)	-0.003 (0.377)	0.784	0.816	-0.059 (0.643)	-0.058 (0.317)	0.520	0.722	-0.21 (1.297)	0.003 (0.585)	1.000	1.000	0.017 (0.164)	-0.017 (0.167)
[1c]:[1] ratio	-0.086 (0.979)	-0.013 (0.373)	0.784	0.816	-0.03 (0.636)	-0.053 (0.154)	0.175	0.416	-0.135 (1.236)	0.058 (0.507)	0.626	0.711	0.029 (0.077)	0.001 (0.051)
[2] Deoxycholic acid (DCA)	-330218 (1006787)	-384000 (1082455)	0.063	0.166	-551022 (800726)	-660180 (461370)	0.042	0.175	-226013 (1153580)	-24750 (1211020)	0.583	0.711	-688472 (1283369)	-1099460 (1480640)
[2] Lithocholic acid	303951 (833101)	135610 (573465)	0.111	0.205	310744 (1023804)	143780 (601430)	0.577	0.780	315620 (723803)	229460 (561950)	0.119	0.229	-330744 (506193)	-316960 (431460)
[2] Ursodeoxycholic acid (UDCA)	126978 (711372)	4770 (56880)	0.544	0.618	-3315 (121742)	-1060 (30130)	0.966	0.966	235464 (966524)	6260 (56160)	0.626	0.711	-101904 (162030)	-20200 (89340)
[2]:[1] ratio	49.5 (73.2)	18.7 (107.4)	0.003	0.014	51.9 (85.6)	23.6 (106.6)	0.102	0.299	53 (64.9)	33.9 (106.4)	0.009	0.039	5.5 (27.2)	5.1 (31.1)
DCA:CA ratio	721 (3003)	4 (280)	0.437	0.521	1123 (4613)	-40 (232)	0.278	0.492	469 (843)	123 (290)	0.013	0.048	-328 (777)	6 (94)
LCA:CDCA ratio	55 (77)	22 (95)	0.000	0.001	67 (101)	26 (106)	0.019	0.103	50 (55)	32 (94)	0.002	0.021	5 (10)	3 (14)
UDCA:CDCA ratio	1.54 (2.12)	0.61 (1.82)	0.000	0.001	1.78 (2.43)	0.66 (3.16)	0.024	0.122	1.46 (1.96)	0.61 (1.68)	0.001	0.020	0.36 (0.63)	0.42 (0.57)
LCA:UDCA ratio	15.56 (71.08)	1.48 (46.69)	0.423	0.521	21.23 (97.85)	0.74 (59.2)	0.898	0.966	13.76 (47.04)	11.52 (26.79)	0.268	0.446	-7.11 (33.99)	3.1 (14.25)
[2c] Glycodeoxycholic acid (GDCA)	-230 (1456)	-164 (1164)	0.437	0.521	-345 (1273)	-333 (690)	0.320	0.517	-356 (1448)	-4 (1531)	0.626	0.711	-584 (980)	-650 (695)
[2c] Glycoursodeoxycholic acid (GUDCA)	2291 (9097)	160 (718)	0.016	0.063	3071 (13342)	245 (539)	0.175	0.416	1827 (4597)	78 (1658)	0.058	0.145	-2502 (5527)	-60 (68)
[2c] Glycolithocholic acid (GLCA)	18.1 (133.3)	35 (121.9)	0.426	0.521	44.5 (153.3)	37.5 (103.8)	0.480	0.707	-8.2 (119.7)	17.5 (123.8)	0.952	0.991	-29 (64.9)	-27.5 (97.5)
[2c] Taurodeoxycholic acid (TDCA)	-140 (3032)	-21 (196)	0.595	0.661	-1191 (4015)	-25 (173)	0.684	0.854	571 (1898)	-23 (231)	0.463	0.626	-2732 (5902)	-68 (238)
[2c] Tauro-ursodeoxycholic acid (TUDCA)	129 (640)	29 (153)	0.081	0.185	137 (654)	28 (30)	0.240	0.480	124 (677)	24 (181)	0.281	0.453	-198 (305)	-18 (258)
2c:2 ratio	0.000 (0.006)	0.000 (0.002)	0.960	0.960	-0.001 (0.008)	0.000 (0.003)	0.898	0.966	0.000 (0.002)	0.000 (0.002)	1.000	1.000	-0.006 (0.011)	-0.007 (0.012)
Conjugated DCA:DCA ratio	-0.001 (0.004)	0.000 (0.003)	0.353	0.465	-0.001 (0.005)	0.000 (0.003)	0.700	0.854	-0.001 (0.003)	0.000 (0.003)	0.241	0.416	-0.004 (0.007)	-0.005 (0.007)
Conjugated UDCA:UDCA ratio	0.059 (0.137)	0.004 (0.038)	0.111	0.205	0.054 (0.14)	0.015 (0.045)	0.240	0.480	0.067 (0.144)	0.004 (0.031)	0.358	0.526	-0.006 (0.013)	-0.008 (0.014)
G:T-DCA ratio	-0.22 (7.78)	-0.02 (10.19)	0.940	0.959	-2.02 (9.11)	0.09 (12.9)	0.765	0.910	1.47 (6.75)	0.39 (8.05)	0.583	0.711	8.84 (10.14)	5.32 (1.86)
G:T-UDCA ratio	1.37 (12.95)	1.71 (7.46)	0.163	0.281	-2.39 (17.71)	4.03 (19.48)	0.966	0.966	4.69 (7.44)	0.93 (9.59)	0.040	0.118	-5.29 (11.54)	-0.33 (3.42)
Isolithocholic acid	72587 (183643)	7860 (131865)	0.190	0.306	48869 (179446)	-7000 (158560)	0.966	0.966	96141 (196589)	42760 (101590)	0.058	0.145	-33768 (54020)	-32520 (50420)
3a-hydroxy-12 ketolithocholic acid (5ß-cholanic acid-3a-ol-12-one)	-71324 (107295)	-55210 (112025)	0.002	0.014	-107596 (127069)	-76980 (73170)	0.003	0.029	-46756 (87876)	-10030 (139395)	0.217	0.387	-6232 (109261)	-36720 (134980)
5ß-Cholanic acid-3ß, 12a-diol	-17529 (49007)	-15600 (52175)	0.059	0.165	-40873 (40840)	-40520 (40440)	0.007	0.057	-1073 (50293)	-3520 (53940)	0.903	0.965	-40512 (62617)	-62080 (61680)
3-Ketocholanic acid	284 (17065)	-346 (14274)	0.653	0.710	-4250 (17928)	-7145 (14403)	0.278	0.492	4431 (16484)	1620 (9819)	0.502	0.643	1698 (14416)	-2555 (13550)
Allolithocholic acid	1282 (4546)	1046 (5188)	0.227	0.334	-158 (6161)	-2028 (4435)	0.365	0.571	2552 (2601)	2370 (2495)	0.002	0.023	-773 (2178)	-525 (3343)
3,6 // 3,12-Diketocholanic Acid	-2390 (4077)	-948 (2694)	0.000	0.001	-3591 (5437)	-1288 (1978)	0.002	0.024	-1560 (2660)	-496 (2852)	0.091	0.189	241 (3803)	-650 (2010)
12-Dehydrocholic acid	-197647 (659617)	-4916 (23431)	0.075	0.179	-201858 (732020)	-2868 (16349)	0.206	0.448	-227018 (638344)	-7754 (216584)	0.091	0.189	-664603 (1445093)	-3060 (85500)
Murocholic acid (MuroCA)	6559 (33544)	1383 (6420)	0.208	0.315	1493 (9556)	785 (11201)	0.465	0.704	10854 (45282)	1923 (6420)	0.391	0.559	-6146 (7670)	-1483 (11363)
5a-Cholanic acid-3a-ol-6-one	1007 (1476)	1008 (1966)	0.003	0.014	1597 (1671)	1870 (2000)	0.018	0.103	560 (1237)	350 (1422)	0.173	0.320	-738 (2963)	133 (845)
9(11), (5B)-Cholenic Acid-3a-ol-12-one	-24 (122)	-34 (144)	0.205	0.315	-68 (86)	-75 (101)	0.032	0.146	1 (139)	-9 (173)	0.796	0.884	-84 (62)	-60 (38)
Ursocholanic acid	0.1 (23.4)	2.5 (8.8)	0.254	0.352	5.5 (24.5)	5 (6.3)	0.093	0.299	-4.1 (23.4)	2.5 (8.8)	0.907	0.965	2 (6.9)	5 (2.5)
Glyco-ursocholanic acid	1.6 (8.7)	2.5 (9.4)	0.107	0.205	2.5 (6.4)	2.5 (7.5)	0.187	0.424	1.1 (10.5)	2.5 (8.8)	0.403	0.560	-1 (7.4)	0 (5)
Tauro-ursocholanic acid (TUCA)	3 (11.7)	2.5 (9.4)	0.147	0.262	2.5 (10.6)	2.5 (10)	0.285	0.492	3.6 (13.3)	3.8 (10.6)	0.317	0.496	-5 (11.6)	-5 (2.5)

Faecal

Serum

	All 3 months (n=45)		Wilcoxon Rank Test		RYGB 3 months (n=18)		Wilcoxon Rank Test		VSG 3 months (n=24)		Wilcoxon Rank Test		1Yr (n=5)	
	Mean change μM (SD)	Median change μM (IQR)	p	pFDR	Mean change μM (SD)	Median change μM (IQR)	p	pFDR	Mean change μM (SD)	Median change μM (IQR)	p	pFDR	Mean change μM (SD)	Median change μM (IQR)
All	-631 (45482)	1582 (40532)	0.955	0.973	9893 (57508)	12532 (57735)	0.212	0.354	-10005 (31647)	-8202 (25939)	0.136	0.246	728 (17121)	-6832 (26327)
All 1	26 (1990)	-104 (840)	0.453	0.580	940 (2355)	258 (1517)	0.130	0.246	-665 (1522)	-293 (924)	0.005	0.033	2000 (3183)	640 (1328)
All 2	1939 (4625)	351 (2215)	0.016	0.051	2946 (5339)	911 (6433)	0.081	0.179	1499 (4237)	357 (1488)	0.016	0.053	847 (1414)	1413 (1080)
All 1c	-10071 (35809)	-3306 (19513)	0.022	0.057	-8442 (43137)	-1796 (16431)	0.523	0.599	-13936 (27268)	-7893 (19160)	0.004	0.027	-1819 (17396)	-3073 (27386)
All 2c	3277 (7531)	404 (5115)	0.015	0.050	6608 (10778)	3525 (10532)	0.048	0.126	1229 (2690)	46 (2936)	0.169	0.273	-208 (1519)	-369 (1658)
All Others	4197 (7346)	1303 (4981)	0.000	0.001	7841 (10092)	4659 (12984)	0.008	0.052	1867 (3189)	1096 (2084)	0.002	0.016	-92 (721)	257 (599)
[1] Cholic acid (CA)	84 (1204)	-48 (320)	0.576	0.689	712 (1405)	132 (1145)	0.081	0.179	-369 (886)	-91 (234)	0.008	0.040	426 (398)	325 (390)
[1] Chenodeoxycholic acid (CDCA)	-58 (1015)	-63 (644)	0.364	0.517	229 (1260)	96 (1232)	0.265	0.383	-296 (806)	-194 (717)	0.012	0.049	1574 (3063)	446 (309)
CA:CDCA ratio	0.102 (1.641)	0.127 (1.146)	0.639	0.747	0.22 (1.149)	0.212 (0.991)	0.347	0.454	-0.008 (1.988)	-0.054 (1.049)	0.790	0.869	0.253 (1.037)	-0.072 (0.286)
[1c] Glycocholic Acid (GCA)	-104 (553)	-85 (286)	0.059	0.112	141 (423)	-31 (423)	0.393	0.480	-302 (585)	-159 (263)	0.000	0.007	127 (299)	14 (53)
[1c] Taurocholic acid (TCA)	-22.08 (67.6)	-8.7 (18.7)	0.001	0.005	-9.93 (47.39)	-4.35 (14.58)	0.246	0.383	-33.82 (81.64)	-11.5 (19.93)	0.000	0.006	2.15 (17.57)	6.19 (9.5)
[1c] Glycochenodeoxycholic acid (GCDCA)	-1075 (5065)	-407 (4397)	0.140	0.231	-246 (6079)	428 (3029)	0.609	0.684	-2102 (3431)	-1430 (3948)	0.003	0.020	-2161 (7460)	-1020 (4292)
[1c] Taurochenodeoxycholic acid (TCDCA)	-8869 (32950)	-4840 (10913)	0.019	0.055	-8327 (40897)	-2195 (11037)	0.325	0.436	-11498 (24819)	-6272 (14247)	0.012	0.049	213 (12148)	-2002 (13107)
G:T 1c ratio	0.178 (0.496)	0.028 (0.167)	0.026	0.059	0.347 (0.691)	0.092 (0.389)	0.024	0.093	0.095 (0.268)	0.038 (0.166)	0.143	0.246	-0.128 (0.32)	-0.021 (0.07)
G:T-CA ratio	4.601 (11.632)	1.559 (10.956)	0.021	0.057	11.917 (14)	8.242 (14.022)	0.000	0.012	0.12 (6.397)	0.404 (7.382)	0.989	0.989	8.028 (9.177)	12.075 (14.621)
G:T-CDCA ratio	0.176 (0.489)	0.044 (0.18)	0.017	0.053	0.338 (0.689)	0.087 (0.378)	0.030	0.104	0.094 (0.25)	0.052 (0.17)	0.101	0.206	-0.137 (0.331)	-0.017 (0.039)
Conjugated CA:CA ratio	-0.645 (6.743)	-0.279 (2.286)	0.069	0.126	0.892 (8.047)	-0.532 (2.744)	0.393	0.480	-1.635 (5.796)	-0.247 (2.276)	0.114	0.224	-2.444 (2.502)	-1.577 (0.881)
TCA:CA ratio	-0.287 (1.138)	-0.021 (0.271)	0.024	0.057	-0.458 (1.651)	-0.039 (0.252)	0.130	0.246	-0.177 (0.64)	-0.02 (0.142)	0.069	0.152	-0.227 (0.195)	-0.174 (0.293)
GCA:CA ratio	-0.358 (6.279)	-0.282 (1.914)	0.082	0.146	1.349 (7.578)	-0.424 (1.765)	0.393	0.480	-1.457 (5.203)	-0.235 (2)	0.121	0.229	-2.217 (2.341)	-1.481 (0.667)
Conjugated CDCA:CDCA ratio	-50 (299.7)	-4.1 (106.4)	0.341	0.506	-165.7 (386.5)	-36.2 (144.4)	0.081	0.179	30 (208.1)	-0.9 (52.3)	0.944	0.962	-150.7 (248.1)	-153.7 (152.6)
[1c]:[1] ratio	-45.5 (252.9)	-3.5 (72.3)	0.276	0.422	-117.5 (378.2)	-20.2 (78.2)	0.154	0.282	3.5 (97)	-2.5 (55.8)	0.705	0.808	-71.2 (58.4)	-72.1 (76.4)
[2] Deoxycholic acid (DCA)	-2.9 (747.3)	-45.9 (684.7)	0.771	0.840	-138.1 (817.4)	-168.8 (762.4)	0.523	0.599	183.7 (646.9)	-23.3 (426.4)	0.422	0.528	1131.9 (1684)	1346.9 (1729.6)
[2] Lithocholic acid (LCA)	8.85 (29.05)	1.6 (23.33)	0.143	0.231	10.65 (34.64)	6.1 (36.14)	0.246	0.383	11.03 (23.97)	0.11 (22.18)	0.178	0.280	6.64 (29.35)	13.72 (23.45)
[2] Ursodeoxycholic acid (UDCA)	1933 (4245)	377 (1174)	0.000	0.002	3073 (4855)	873 (5092)	0.009	0.052	1305 (3898)	323 (761)	0.007	0.036	-291 (755)	-3 (68)
2:1 ratio	3.484 (12.1)	0.351 (3.508)	0.056	0.110	5.707 (16.349)	0.38 (5.45)	0.304	0.418	2.501 (8.57)	0.946 (2.892)	0.016	0.053	-2.628 (3.407)	-1.777 (2.911)
DCA:CA ratio	-1.992 (9.335)	-0.034 (5.633)	0.257	0.404	-4.317 (9.48)	-1.732 (5.731)	0.043	0.125	0.877 (7.971)	0.224 (3.043)	0.345	0.486	3.141 (21.639)	2.558 (5.241)
LCA:CDCA ratio	0.043 (0.465)	0.013 (0.122)	0.407	0.546	-0.034 (0.286)	0.011 (0.194)	0.899	0.915	0.114 (0.584)	0.016 (0.099)	0.084	0.178	-0.302 (0.355)	-0.082 (0.498)
UDCA:CDCA ratio	6.538 (15.021)	1.047 (6.058)	0.000	0.001	10.941 (20.849)	3.862 (10.498)	0.010	0.052	4.044 (9.123)	0.852 (5.087)	0.000	0.007	-1.65 (2.572)	-0.702 (1.606)
UDCA:LCA ratio	-27.11 (499.36)	21.88 (88.99)	0.000	0.003	-106.24 (791.65)	78.88 (86.67)	0.012	0.055	23.72 (72.57)	6.73 (27.61)	0.056	0.129	-639.31 (1431.16)	-0.01 (3.24)
LCA:UDCA ratio	-0.167 (0.24)	-0.085 (0.335)	0.000	0.001	-0.221 (0.237)	-0.2 (0.329)	0.001	0.012	-0.124 (0.247)	-0.044 (0.224)	0.016	0.053	-0.125 (0.209)	0.001 (0.266)
[2c] Glycolithocholic acid (GLCA)	14.87 (114.88)	2.66 (31.5)	0.134	0.230	13.62 (153.07)	8.87 (40.17)	0.074	0.179	20.39 (87.52)	0.17 (17.44)	0.410	0.528	7.72 (26.46)	7.94 (12.81)
[2c] Glycodeoxycholic acid (GDCA)	-150 (1112)	-36 (756)	0.376	0.517	-373 (1386)	-332 (1290)	0.284	0.400	92 (780)	-20 (308)	0.855	0.888	27 (1178)	268 (1097)
[2c] Glycoursodeoxycholic acid (GUDCA)	3442 (6861)	222 (4031)	0.000	0.003	7091 (9562)	3262 (9230)	0.006	0.051	1058 (2226)	144 (676)	0.027	0.071	-164 (283)	-41 (123)
[2c] Taurolithocholic acid (TLCA)	0.871 (19.967)	0.076 (3.914)	0.779	0.840	-3.788 (23.701)	-0.38 (4.693)	0.640	0.704	5.35 (16.982)	0.19 (4.218)	0.280	0.416	-3.513 (10.337)	0.332 (13.908)
[2c] Tauro-ursodeoxycholic acid (TUDCA)	102.21 (199.97)	7.87 (105.72)	0.001	0.005	189.13 (253.13)	51.57 (393.21)	0.010	0.052	45.77 (137.61)	5.02 (30.11)	0.039	0.099	-4.61 (3.49)	-5.51 (3.19)
[2c] Taurodeoxycholic acid (TDCA)	-132.7 (601.5)	-11.8 (167.7)	0.048	0.097	-310.1 (909.7)	-90 (261.6)	0.016	0.067	8.8 (134.7)	-10.9 (65.3)	0.684	0.800	-70.9 (288.9)	15.7 (350.8)
[2c]:[2] ratio	0.698 (5.044)	-0.034 (1.869)	0.947	0.973	1.125 (7.335)	0.011 (2.356)	0.932	0.932	0.384 (2.883)	-0.103 (1.287)	0.768	0.862	-0.134 (1.076)	-0.003 (0.383)
Conjugated DCA:DCAratio	-0.149 (2.713)	-0.178 (2.115)	0.495	0.605	-0.634 (2.86)	-0.475 (1.58)	0.196	0.338	0.242 (2.742)	-0.061 (1.746)	0.812	0.875	-0.16 (1.084)	-0.129 (0.671)
Conjugated UDCA:UDCA ratio	1.264 (5.855)	-0.169 (1.625)	0.737	0.828	2.549 (8.604)	0.227 (2.573)	0.265	0.383	0.374 (2.89)	-0.266 (0.973)	0.375	0.515	-0.722 (1.076)	-0.143 (1.543)
Conjugated LCA:LCA ratio	0.156 (1.941)	-0.058 (1.672)	0.973	0.973	0.089 (2.455)	-0.137 (2.229)	0.899	0.915	0.274 (1.63)	0.063 (1.55)	0.663	0.793	0.326 (0.88)	0.78 (1.429)
G:T-DCA ratio	2.243 (6.086)	1.152 (4.55)	0.023	0.057	4.668 (5.797)	2.832 (4.761)	0.001	0.012	1.162 (5.875)	-0.04 (3.615)	0.833	0.881	4.232 (4.75)	2.799 (4.958)
G:T-UDCA ratio	10.422 (25.228)	5.782 (15.886)	0.002	0.009	19.686 (33.647)	12.66 (25.984)	0.002	0.027	6.357 (14.587)	5.753 (16.745)	0.056	0.129	8.177 (8.684)	10.409 (12.717)
G:T-LCA ratio	-1.534 (27.048)	0.184 (9.849)	0.370	0.517	0.854 (39.212)	5.532 (7.607)	0.034	0.111	-3.247 (15.912)	-0.872 (7.014)	0.390	0.524	-20.791 (53.713)	1.258 (1.41)
Murocholic acid (MuroCA)	370 (890)	182 (713)	0.000	0.003	225 (659)	190 (719)	0.108	0.221	497 (1065)	176 (660)	0.002	0.016	-205 (479)	-30 (141)
Taurohyocholic acid (THCA)	285 (851)	28 (289)	0.031	0.066	452 (1215)	34 (298)	0.048	0.126	178 (503)	9 (150)	0.422	0.528	105 (149)	45 (55)
12-Dehydrocholic acid	68.2 (214)	29.5 (116.5)	0.006	0.022	59.6 (188.3)	36.6 (113.7)	0.265	0.383	80.1 (246.7)	30.7 (179.4)	0.013	0.050	89.3 (169)	4.6 (57.2)
5ß-Cholanic acid-3ß, 12a-diol	-8.9 (59.67)	-0.23 (30.78)	0.420	0.550	-34.8 (58.95)	-8.91 (50.79)	0.038	0.118	15.05 (51.87)	1.52 (25.87)	0.160	0.267	46.29 (106)	43.59 (118.71)
5-Cholenic acid-3ß-ol	3.57 (7.84)	2.58 (8.59)	0.003	0.014	2.47 (7.91)	2.24 (6.37)	0.099	0.209	4.55 (8.27)	3.86 (9.79)	0.027	0.071	4.8 (9.37)	3 (13.26)
3a-hydroxy-7 ketolithocholic acid (5ß-cholanic acid-3a-ol-7-one)	15.84 (49.64)	8.7 (43.02)	0.029	0.064	26.02 (47.26)	21.58 (43.91)	0.027	0.098	7.95 (53.45)	5.68 (45.01)	0.603	0.737	-6.63 (22.05)	0.72 (10.79)
Isolithocholic acid	11.7 (33.97)	1.9 (16.42)	0.013	0.048	6.39 (28.39)	1.92 (23.27)	0.468	0.560	19 (37.56)	2.34 (16.34)	0.000	0.006	5.78 (26.43)	5.62 (28.5)
3a-hydroxy-12 ketolithocholic acid (5ß-cholanic acid-3a-ol-12-one)	-0.98 (20)	0.15 (6.76)	0.797	0.843	-10.58 (25.47)	-1.77 (16.69)	0.167	0.297	6.83 (12.02)	1.67 (13.95)	0.025	0.071	11.28 (11.99)	7.41 (0.87)
Glycohyocholic acid (GHCA)	14.7 (33.65)	1.75 (15.81)	0.001	0.006	26.06 (46.89)	7.33 (30.43)	0.004	0.046	7.14 (18.94)	0.61 (8.56)	0.141	0.246	10.78 (19.38)	1.48 (4.75)
Allolithocholic acid	1.09 (5.52)	-0.08 (4.56)	0.693	0.795	-0.09 (3.7)	-0.44 (4.05)	0.710	0.766	2.37 (6.61)	0.15 (5.93)	0.243	0.372	0.36 (2.74)	1.44 (0.27)
8(14),(5ß)-Cholenic acid-3a, 12a-diol	-0.12 (3.33)	-0.11 (1.29)	0.472	0.590	-0.5 (4.7)	-0.04 (1.62)	0.899	0.915	0.02 (2)	-0.29 (1.25)	0.320	0.463	-1.25 (4.25)	-0.15 (0.76)

Serum

Supplementary Data 7 – Change in measured bile acids in serum and faeces in participants post Roux-en-Y Gastric Bypass (RYGB) and Vertical Sleeve Gastrectomy (VSG) procedures.

Supplementary Data 8 – Change in SCFA and other carboxylic acids post bariatric surgery in faecal, urine and serum samples

Serum

	All 3 months (n=49 paired)		Wilcoxon Rank Test		RYGB 3 months (n=23 paired)		Wilcoxon Rank Test		VSG 3 months (n=26 paired)		Wilcoxon Rank Test		All 1 Yr (n=9 paired)		Wilcoxon Rank Test	
	Mean change µM (SD)	Median change µM (IQR)	p	pFDR	Mean change µM (SD)	Median change µM (IQR)	p	pFDR	Mean change µM (SD)	Median change µM (IQR)	p	pFDR	Mean change µM (SD)	Median change µM (IQR)	p	pFDR
Acetic acid	3.4 (26.7)	3.2 (30.9)	0.410	0.558	0.4 (23.5)	3.1 (25.5)	0.540	0.665	5.9 (29.3)	5.4 (40)	0.413	0.534	-7.1 (32.2)	6.5 (28.4)	1.000	1.000
Propionic acid	-0.7 (2.73)	-0.2 (3.7)	0.121	0.261	-0.64 (2.78)	-0.16 (3.01)	0.360	0.562	-0.75 (2.73)	-0.24 (3.91)	0.248	0.497	-0.77 (3.22)	-2.17 (5.63)	0.496	0.932
Isobutyric acid	-0.11 (0.61)	-0.18 (0.95)	0.253	0.422	-0.13 (0.59)	-0.14 (0.67)	0.393	0.562	-0.09 (0.64)	-0.24 (1.04)	0.427	0.534	-0.16 (0.59)	-0.29 (0.8)	0.570	0.932
Butyric acid	-0.04 (0.57)	0.00 (0.58)	0.804	0.856	-0.03 (0.46)	-0.12 (0.58)	0.665	0.665	-0.06 (0.66)	0.01 (0.41)	0.953	0.953	-0.13 (0.56)	-0.06 (0.74)	0.652	0.932
2 Methylbutyric acid	-0.02 (0.07)	-0.01 (0.06)	0.034	0.168	-0.02 (0.08)	-0.01 (0.06)	0.234	0.469	-0.02 (0.05)	-0.02 (0.06)	0.081	0.324	-0.01 (0.07)	-0.01 (0.08)	0.910	1.000
Isovaleric acid	-0.04 (0.17)	-0.04 (0.2)	0.093	0.261	-0.05 (0.15)	-0.04 (0.17)	0.160	0.469	-0.04 (0.18)	-0.04 (0.22)	0.336	0.534	0.05 (0.18)	0.03 (0.13)	0.496	0.932
Valeric acid	0.004 (0.04)	0.000 (0.06)	0.446	0.558	0.00 (0.05)	0.00 (0.04)	0.609	0.665	0.01 (0.04)	0.00 (0.05)	0.097	0.324	0.01 (0.06)	0.02 (0.04)	0.461	0.932
Caproic acid	0.005 (0.18)	0.017 (0.25)	0.856	0.856	-0.05 (0.16)	-0.02 (0.21)	0.211	0.469	0.05 (0.18)	0.09 (0.26)	0.220	0.497	-0.03 (0.19)	-0.01 (0.23)	0.820	1.000
Lactic acid	-359 (787)	-165 (798)	0.004	0.037	-493 (911)	-344 (1166)	0.023	0.233	-244 (660)	-146 (626)	0.058	0.324	-432 (917)	27 (1379)	0.426	0.932
2 Hydroxybutyric acid	-8.8 (46)	-9.9 (40.8)	0.130	0.261	-12.43 (55.82)	-12.86 (44.6)	0.119	0.469	-5.79 (36.38)	-7.25 (40.72)	0.594	0.660	-28.47 (21.17)	-26.16 (25.69)	0.008	0.078

Urine

	All 3 months (n=30 paired)		Wilcoxon Rank Test		RYGB 3 months (n=14 paired)		Wilcoxon Rank Test		VSG 3 months (n=16 paired)		Wilcoxon Rank Test		All 1 Yr (n=8 paired)		Wilcoxon Rank Test	
	Mean change µM (SD)	Median change µM (IQR)	p	pFDR	Mean change µM (SD)	Median change µM (IQR)	p	pFDR	Mean change µM (SD)	Median change µM (IQR)	p	pFDR	Mean change µM (SD)	Median change µM (IQR)	p	pFDR
Acetic acid	-464 (2187)	-41 (240)	0.480	0.884	-823 (3165)	-11 (276)	0.670	1.000	-169 (788)	-56 (232)	0.678	0.963	-1289 (5301)	5 (956)	0.742	0.825
A (Osmo norm)	-356 (1813)	-30 (566)	0.568	0.884	-532 (2675)	31 (772)	1.000	1.000	-212 (550)	-39 (627)	0.329	0.963	-831 (4347)	441 (834)	0.250	0.625
A (Creat norm)	-502 (2459)	-14 (512)	0.456	0.884	-792 (3651)	26 (810)	0.903	1.000	-263 (615)	-63 (482)	0.243	0.963	-1202 (5710)	644 (1243)	0.313	0.658
Propionic acid	-0.9 (89.9)	13.1 (47.5)	0.176	0.884	-6.2 (127.9)	14.6 (65.2)	0.153	0.724	3.4 (42.8)	6.5 (44.8)	0.712	0.963	-28.3 (172.7)	25.1 (45.6)	0.195	0.625
P (Osmo norm)	27 (127.5)	9.5 (94.7)	0.281	0.884	58.9 (174.1)	41.4 (167.2)	0.135	0.724	0.8 (65)	-19.2 (60.4)	0.927	0.963	12.5 (169.3)	45.9 (105.6)	0.461	0.658
P (Creat norm)	26 (161)	16 (93)	0.410	0.884	62 (221.2)	22.3 (171.5)	0.194	0.724	-3.6 (81.4)	-8.8 (100.9)	0.890	0.963	14.4 (245.7)	43.5 (140)	0.547	0.684
Isobutyric acid	0.34 (2.67)	-0.07 (2.44)	0.839	0.884	0.53 (1.45)	-0.05 (2.02)	0.463	0.926	0.19 (3.41)	-0.75 (3.09)	0.712	0.963	1.76 (3.35)	1.27 (1.62)	0.148	0.557
IB (Osmo norm)	2.95 (10.67)	1.05 (8.62)	0.256	0.884	7.38 (13.31)	1.46 (14.58)	0.135	0.724	-0.69 (6.18)	0.12 (9.53)	0.963	0.963	4.88 (12.04)	2.46 (13.86)	0.250	0.625
IB (Creat norm)	3.02 (13.4)	0.02 (8.48)	0.839	0.884	8.02 (17.25)	0.49 (8.7)	0.391	0.926	-1.1 (7.41)	-1.21 (6.67)	0.579	0.963	6.95 (19.75)	2.72 (19.42)	0.461	0.658
Butyric acid	0.25 (4.06)	-0.15 (1.7)	0.568	0.884	0.18 (2.44)	-0.22 (1.78)	0.761	1.000	0.31 (5.1)	-0.15 (1.63)	0.678	0.963	2.29 (4.79)	1.1 (2.55)	0.148	0.557
B (Osmo norm)	2.1 (8.7)	-0.07 (8.49)	0.456	0.884	5.42 (10.8)	1.57 (13.83)	0.241	0.724	-0.64 (5.42)	-0.22 (8.3)	0.818	0.963	6.51 (14.74)	1.35 (11.55)	0.313	0.658
B (Creat norm)	2.06 (10.82)	-0.5 (6.81)	0.839	0.884	5.89 (13.98)	-0.17 (11.3)	0.761	1.000	-1.09 (6.13)	-2.38 (6.3)	0.517	0.963	8.77 (21.2)	1.63 (18.8)	0.383	0.658
2 Methylbutyric acid	0.25 (0.83)	-0.02 (0.25)	0.399	0.884	0.27 (0.69)	-0.03 (0.38)	0.808	1.000	0.24 (0.95)	0.08 (0.21)	0.459	0.963	0.14 (0.48)	-0.06 (0.33)	1.000	1.000
2MB (Osmo norm)	0.22 (0.54)	0.08 (0.38)	0.058	0.884	0.37 (0.6)	0.16 (0.49)	0.035	0.724	0.1 (0.47)	0.02 (0.3)	0.644	0.963	0.39 (1.14)	0.14 (0.35)	0.461	0.658
2MB (Creat norm)	0.21 (0.59)	0.03 (0.53)	0.209	0.884	0.39 (0.72)	0.17 (0.67)	0.194	0.724	0.06 (0.43)	0.03 (0.29)	0.927	0.963	0.49 (1.4)	0.09 (0.55)	0.547	0.684
Isovaleric acid	0.25 (0.97)	0.06 (0.24)	0.169	0.884	0.22 (0.61)	0.06 (0.24)	0.583	0.972	0.27 (1.21)	0.06 (0.19)	0.284	0.963	0.24 (0.7)	-0.05 (0.47)	0.844	0.873
IV (Osmo norm)	0.23 (0.67)	0.04 (0.4)	0.076	0.884	0.39 (0.72)	0.15 (0.5)	0.049	0.724	0.1 (0.61)	0.04 (0.28)	0.747	0.963	0.61 (1.6)	0.08 (0.63)	0.461	0.658
IV (Creat norm)	0.22 (0.71)	0.01 (0.52)	0.480	0.884	0.42 (0.83)	0.04 (0.99)	0.194	0.724	0.05 (0.57)	-0.04 (0.35)	0.747	0.963	0.77 (1.94)	0.06 (1.1)	0.383	0.658
Valeric acid	0.06 (0.56)	0 (0.38)	0.824	0.884	0.15 (0.74)	-0.02 (0.47)	1.000	1.000	-0.02 (0.36)	0.03 (0.35)	0.712	0.963	0.14 (0.38)	0.02 (0.25)	0.547	0.684
V (Osom norm)	0.2 (0.91)	-0.04 (0.63)	0.721	0.884	0.5 (1.16)	0.12 (0.71)	0.241	0.724	-0.05 (0.56)	-0.05 (0.42)	0.517	0.963	0.29 (0.7)	0.21 (0.65)	0.250	0.625
V (Creat norm)	0.22 (1)	-0.03 (0.55)	0.931	0.931	0.54 (1.27)	0.04 (1.04)	0.463	0.926	-0.05 (0.64)	-0.09 (0.53)	0.487	0.963	0.42 (1.1)	0.19 (0.95)	0.383	0.658
Caproic acid	-0.1 (0.67)	-0.06 (0.52)	0.347	0.884	-0.11 (0.45)	-0.11 (0.52)	0.426	0.926	-0.09 (0.81)	-0.05 (0.46)	0.678	0.963	-0.03 (0.54)	-0.13 (0.43)	0.742	0.825
C (Osmo norm)	0.23 (1.67)	-0.03 (1.05)	0.809	0.884	0.73 (2.03)	0.03 (1.21)	0.463	0.926	-0.18 (1.22)	-0.03 (1.04)	0.782	0.963	0.23 (1.48)	0.04 (0.75)	0.742	0.825
C (Creat norm)	0.26 (1.93)	-0.1 (0.78)	0.595	0.884	0.78 (2.37)	-0.09 (0.67)	1.000	1.000	-0.18 (1.41)	-0.1 (0.9)	0.487	0.963	0.45 (2.42)	0.02 (1.41)	0.844	0.873
Lactic acid	-211 (602)	-4 (282)	0.433	0.884	-176 (524)	-2 (386)	0.542	0.956	-240 (673)	-19 (126)	0.548	0.963	-253 (449)	-99 (199)	0.109	0.547
L (Osmo norm)	-213 (902)	-26 (301)	0.517	0.884	-17 (602)	-16 (328)	0.670	1.000	-374 (1081)	-26 (139)	0.782	0.963	-208 (368)	-77 (189)	0.078	0.547
L (Creat norm)	-226 (975)	-24 (342)	0.410	0.884	-46 (763)	47 (546)	0.903	1.000	-374 (1121)	-36 (187)	0.329	0.963	-223 (472)	-42 (134)	0.109	0.547
2 Hydroxybutyric acid	-6.84 (23.4)	-0.06 (9.62)	0.735	0.884	-7.01 (18.1)	-0.33 (21.27)	0.502	0.940	-6.7 (27.57)	0.57 (6.69)	0.890	0.963	-8.35 (9.47)	-4.01 (13.9)	0.008	0.234
2HB (Osmo norm)	-4.73 (27.63)	0.16 (10.11)	0.854	0.884	-0.51 (21.85)	-0.78 (20.51)	0.903	1.000	-8.2 (31.86)	0.53 (5.85)	0.963	0.963	-8.37 (10.19)	-4.51 (10.77)	0.016	0.234
2HB (Creat norm)	-5 (27.7)	0.87 (12.29)	0.706	0.884	-1.73 (28.21)	0.16 (24.54)	0.903	1.000	-7.7 (27.84)	0.89 (5.57)	0.747	0.963	-8.72 (9.48)	-6.77 (12.08)	0.039	0.391

Faeces

	All 3 months (n=28)		Wilcoxon Rank Test		RYGB 3 months (n=14)		Wilcoxon Rank Test		VSG 3 months (n=14)		Wilcoxon Rank Test		All 1 Yr (n=9)		Wilcoxon Rank Test	
	Mean change µM (SD)	Median change µM (IQR)	p	pFDR	Mean change µM (SD)	Median change µM (IQR)	p	pFDR	Mean change µM (SD)	Median change µM (IQR)	p	pFDR	Mean change µM (SD)	Median change µM (IQR)	p	pFDR
Acetic Acid	-19426 (21488)	-17635 (27688)	0.000	0.000	-21964 (19067)	-20293 (26802)	0.003	0.034	-19399 (23133)	-16526 (24325)	0.007	0.034	-27970 (25947)	-18275 (18034)	0.008	0.078
Propionic Acid	-3307 (9993)	-5601 (12203)	0.053	0.178	-4141 (10585)	-9083 (14255)	0.244	0.488	-3280 (9741)	-5290 (9802)	0.104	0.208	-8171 (10820)	-6865 (5751)	0.055	0.273
Isobutyric Acid	13 (1110)	12 (1128)	0.678	0.678	498 (1261)	239 (818)	0.191	0.477	-318 (724)	-342 (879)	0.091	0.208	-74 (1253)	198 (1495)	0.820	1.000
Butyric Acid	-5763 (13193)	-5704 (15935)	0.020	0.102	-1407 (14521)	1726 (16710)	0.787	0.893	-10241 (11112)	-7188 (12946)	0.005	0.034	-6107 (14969)	-1725 (5380)	0.301	0.752
2 Methylbutyric Acid	149 (726)	94 (764)	0.399	0.499	513 (756)	418 (1159)	0.048	0.160	-92 (480)	-25 (542)	0.542	0.542	33 (690)	316 (771)	0.734	1.000
Isovaleric Acid	367 (1278)	42 (1211)	0.305	0.436	1058 (1413)	782 (1843)	0.010	0.052	-154 (746)	-124 (610)	0.296	0.329	212 (1237)	726 (1510)	0.496	0.992
Valeric Acid	-323 (2069)	-429 (1615)	0.227	0.436	21 (2747)	272 (2588)	0.839	0.893	-601 (1302)	-733 (1303)	0.025	0.082	-242 (2112)	721 (2869)	0.910	1.000
Caproic Acid	-139 (694)	-31 (271)	0.227	0.436	106 (625)	-35 (239)	0.893	0.893	-310 (709)	-18 (630)	0.268	0.329	-78 (595)	17 (82)	1.000	1.000
Lactic Acid	-83 (415)	-4 (94)	0.582	0.647	5 (102)	12 (87)	0.588	0.840	-175 (574)	-24 (108)	0.135	0.225	-168 (738)	29 (73)	0.652	1.000
2 Hydroxybutyric Acid	-1.18 (14.82)	-0.36 (3.87)	0.264	0.436	2.80 (15.39)	-0.45 (3.25)	0.340	0.566	-5.47 (14.02)	-0.65 (3.85)	0.296	0.329	-7.46 (15.41)	-0.74 (7.69)	0.129	0.430

Supplementary Data 8 – Change in SCFAs and other carboxylic acids in serum, urine and faeces of participants post Roux-en-Y Gastric Bypass (RYGB) and Vertical Sleeve Gastrectomy (VSG) procedures.

Supplementary Data 9 – Changes in serum metabolites post bariatric surgery

		All 3 months (n=49 paired)				Wilcoxon Rank Test		RYGB 3 months (n=23 paired)				Wilcoxon Rank Test		VSG 3 months (n=26 paired)				Wilcoxon Rank Test		
		Mean (µM)	Median (µM)	Mean (µM)	Median (µM)	p	pFDR	Mean (µM)	Median (µM)	Mean (µM)	Median (µM)	p	pFDR	Mean (µM)	Median (µM)	Mean (µM)	Median (µM)	p	pFDR	
		Pre	Pre	Post	Post			Pre	Pre	Post	Post			Pre	Pre	Post	Post			
Acylcarnitines	Carnitine (C0)	41.53	41.90	38.16	37.30	0.041	0.071	40.41	38.30	39.26	39.30	0.386	0.561	42.51	43.15	37.19	35.50	0.036	0.080	Acylcarnitines
	Acetylcarnitine (C2)	6.81	6.09	9.30	8.53	0.000	0.001	7.66	6.94	10.24	9.38	0.019	0.074	6.05	5.76	8.47	8.10	0.007	0.028	
	Propionylcarnitine (C3)	0.369	0.332	0.262	0.245	0.000	0.001	0.390	0.332	0.284	0.249	0.018	0.071	0.350	0.335	0.241	0.242	0.001	0.005	
	Hydroxybutyrylcarnitine (C4-OH)	0.074	0.061	0.098	0.090	0.009	0.023	0.081	0.076	0.112	0.106	0.073	0.173	0.068	0.056	0.086	0.077	0.066	0.122	
	Hydroxypropionylcarnitine (C3-OH)	0.014	0.013	0.011	0.011	0.003	0.009	0.013	0.013	0.012	0.011	0.248	0.404	0.014	0.013	0.011	0.011	0.001	0.009	
	Propenoylcarnitine (C3:1)	0.011	0.011	0.009	0.008	0.002	0.007	0.011	0.011	0.010	0.008	0.116	0.239	0.012	0.012	0.009	0.008	0.005	0.020	
	Butyrylcarnitine (C4)	0.210	0.186	0.177	0.153	0.016	0.034	0.213	0.186	0.207	0.176	0.574	0.726	0.207	0.185	0.151	0.138	0.002	0.010	
	Butenylcarnitine (C4:1)	0.013	0.012	0.014	0.013	0.226	0.288	0.013	0.013	0.016	0.015	0.117	0.239	0.013	0.012	0.013	0.013	0.920	0.925	
	Valerylcarnitine (C5)	0.151	0.144	0.107	0.098	0.000	0.000	0.151	0.128	0.118	0.101	0.029	0.088	0.151	0.155	0.097	0.096	0.000	0.003	
	Glutarylcarnitine (C6-OH)	0.025	0.023	0.028	0.025	0.175	0.238	0.027	0.024	0.031	0.029	0.110	0.231	0.023	0.022	0.025	0.023	0.476	0.574	
	Methylglutarylcarnitine (C5-M-DC)	0.026	0.022	0.028	0.022	0.679	0.752	0.030	0.024	0.036	0.024	0.160	0.301	0.022	0.021	0.021	0.021	0.524	0.620	
	Hydroxyvalerylcarnitine (C5-OH)	0.035	0.033	0.030	0.028	0.000	0.000	0.036	0.031	0.031	0.028	0.010	0.048	0.035	0.036	0.028	0.028	0.000	0.004	
	Tiglylcarnitine (C5:1)	0.022	0.021	0.020	0.019	0.007	0.018	0.022	0.021	0.021	0.020	0.380	0.557	0.021	0.021	0.018	0.019	0.002	0.011	
	Glutaconylcarnitine (C5:1-DC)	0.017	0.016	0.016	0.015	0.043	0.072	0.016	0.015	0.016	0.016	0.948	0.996	0.017	0.018	0.015	0.015	0.012	0.045	
	Fumarylcarnitine (C6)	0.075	0.074	0.076	0.073	0.731	0.804	0.073	0.077	0.077	0.073	0.889	0.958	0.076	0.071	0.075	0.069	0.583	0.663	
	Fumarylcarnitine (C6:1)	0.015	0.014	0.014	0.013	0.223	0.286	0.015	0.014	0.015	0.015	1.000	1.000	0.015	0.014	0.013	0.013	0.101	0.173	
	Pimeloylcarnitine (C7-DC)	0.028	0.023	0.036	0.032	0.009	0.023	0.032	0.022	0.040	0.033	0.061	0.152	0.024	0.024	0.032	0.032	0.005	0.020	
	Octanoylcarnitine (C8)	0.158	0.139	0.194	0.173	0.114	0.166	0.150	0.139	0.189	0.176	0.023	0.081	0.166	0.142	0.198	0.159	0.541	0.635	
	Nonaylcarnitine (C9)	0.029	0.024	0.025	0.021	0.082	0.125	0.029	0.022	0.027	0.021	0.276	0.443	0.028	0.026	0.024	0.024	0.198	0.290	
	Decanoylcarnitine (C10)	0.210	0.175	0.276	0.218	0.019	0.038	0.203	0.175	0.279	0.261	0.039	0.110	0.216	0.173	0.274	0.206	0.303	0.405	
	Decenoylcarnitine (C10:1)	0.335	0.316	0.356	0.357	0.283	0.344	0.340	0.342	0.378	0.370	0.207	0.356	0.330	0.304	0.336	0.309	0.708	0.776	
	Decadienylcarnitine (C10:2)	0.055	0.053	0.051	0.049	0.042	0.071	0.058	0.055	0.056	0.053	0.584	0.729	0.053	0.050	0.047	0.045	0.015	0.047	
	Dodecanoylcarnitine (C12)	0.092	0.081	0.101	0.092	0.268	0.330	0.093	0.081	0.102	0.107	0.420	0.588	0.091	0.085	0.099	0.087	0.547	0.638	
	Dodecanedioylcarnitine (C12-DC)	0.045	0.044	0.045	0.044	0.831	0.892	0.044	0.045	0.046	0.044	0.330	0.503	0.047	0.044	0.044	0.044	0.300	0.404	
	Dodecenoylcarnitine (C12:1)	0.232	0.225	0.263	0.253	0.070	0.110	0.244	0.231	0.287	0.281	0.089	0.198	0.222	0.218	0.242	0.221	0.449	0.548	
	Tetradecanoylcarnitine (C14)	0.063	0.061	0.065	0.063	0.234	0.294	0.064	0.061	0.068	0.066	0.175	0.315	0.062	0.061	0.063	0.061	0.716	0.780	
	Tetradecenoylcarnitine (C14:1)	0.063	0.060	0.074	0.068	0.042	0.071	0.062	0.058	0.078	0.073	0.040	0.113	0.064	0.065	0.071	0.065	0.419	0.519	
	Hydroxytetradecenoylcarnitine (C14:1-OH)	0.013	0.013	0.015	0.014	0.031	0.057	0.014	0.013	0.016	0.015	0.054	0.141	0.013	0.014	0.014	0.014	0.299	0.404	
	Tetradecadienylcarnitine (C14:2)	0.024	0.022	0.030	0.027	0.032	0.057	0.024	0.022	0.031	0.030	0.103	0.229	0.024	0.024	0.030	0.027	0.171	0.257	
	Hydroxytetradecadienylcarnitine (C14:2-OH)	0.007	0.007	0.006	0.006	0.504	0.572	0.007	0.006	0.007	0.006	0.712	0.847	0.007	0.007	0.006	0.006	0.363	0.468	
	Hexadecanoylcarnitine (C16)	0.102	0.102	0.123	0.117	0.000	0.001	0.101	0.102	0.126	0.117	0.004	0.026	0.103	0.102	0.120	0.118	0.015	0.047	
	Hydroxyhexadecanoylcarnitine (C16-OH)	0.006	0.006	0.007	0.007	0.051	0.083	0.007	0.006	0.008	0.007	0.405	0.583	0.006	0.006	0.007	0.007	0.057	0.113	
	Hexadecenoylcarnitine (C16:1)	0.027	0.026	0.033	0.030	0.020	0.039	0.027	0.025	0.035	0.035	0.036	0.104	0.026	0.027	0.031	0.029	0.222	0.316	
	Hydroxyhexadecenoylcarnitine (C16:1-OH)	0.011	0.010	0.014	0.012	0.000	0.001	0.011	0.010	0.014	0.013	0.003	0.025	0.011	0.010	0.013	0.012	0.018	0.053	
	Hexadecadienylcarnitine (C16:2)	0.009	0.008	0.010	0.009	0.083	0.125	0.008	0.007	0.010	0.009	0.077	0.181	0.009	0.009	0.010	0.009	0.466	0.565	
	Hydroxyhexadecadienylcarnitine (C16:2-OH)	0.013	0.013	0.014	0.013	0.048	0.078	0.013	0.013	0.014	0.013	0.062	0.152	0.013	0.013	0.014	0.014	0.404	0.503	
	Octadecanoylcarnitine (C18)	0.035	0.033	0.037	0.036	0.314	0.377	0.035	0.033	0.037	0.037	0.306	0.478	0.035	0.035	0.037	0.036	0.837	0.875	
	Octadecenoylcarnitine (C18:1)	0.118	0.112	0.168	0.156	0.000	0.000	0.124	0.108	0.177	0.172	0.000	0.009	0.112	0.115	0.160	0.151	0.000	0.004	
	Hydroxyoctadecenoylcarnitine (C18:1-OH)	0.009	0.008	0.011	0.010	0.000	0.001	0.009	0.008	0.012	0.011	0.003	0.025	0.009	0.009	0.010	0.010	0.051	0.103	
	Octadecadienylcarnitine (C18:2)	0.039	0.038	0.047	0.045	0.003	0.009	0.037	0.038	0.046	0.044	0.001	0.016	0.041	0.038	0.048	0.046	0.162	0.246	
Amino Acids	Alanine	439.612	415.000	365.122	364.000	0.000	0.001	444.261	415.000	371.261	347.000	0.009	0.046	435.500	412.000	359.692	371.500	0.002	0.009	Amino Acids
	Arginine	111.863	108.000	103.953	101.000	0.078	0.119	102.635	98.500	102.004	97.600	0.855	0.941	120.027	116.000	105.677	104.500	0.025	0.066	
	Asparagine	43.412	41.200	40.202	38.900	0.054	0.087	41.235	38.800	39.500	38.900	0.308	0.478	45.338	42.350	40.823	39.000	0.088	0.154	
	Aspartate	25.441	23.800	22.792	22.100	0.014	0.031	24.383	22.500	22.709	22.100	0.186	0.329	26.377	24.450	22.866	21.800	0.032	0.075	
	Citrulline	26.435	26.000	24.316	21.300	0.189	0.253	25.887	24.800	24.739	21.200	0.796	0.897	26.919	27.050	23.942	23.250	0.134	0.214	
	Glutamine	611.347	612.000	659.776	653.000	0.006	0.016	594.087	601.000	662.000	653.000	0.010	0.048	626.615	616.500	657.808	652.500	0.158	0.242	
	Glutamate	94.331	97.300	67.802	63.700	0.000	0.001	93.422	102.000	70.843	60.200	0.055	0.141	95.135	92.250	65.112	65.600	0.000	0.003	
	Glycine	234.286	224.000	299.224	292.000	0.000	0.000	231.957	229.000	319.870	297.000	0.000	0.009	236.346	220.500	280.962	270.500	0.007	0.028	
	Histidine	79.127	77.100	74.743	74.300	0.110	0.163	77.117	73.900	73.800	73.700	0.584	0.729	80.904	78.550	75.577	76.350	0.109	0.180	
	Isoleucine	89.167	81.600	68.602	59.400	0.000	0.001	86.039	75.700	68.022	59.400	0.004	0.025	91.935	86.850	69.115	58.750	0.013	0.045	
	Leucine	160.155	146.000	122.890	110.000	0.000	0.001	155.057	136.000	119.452	105.000	0.005	0.030	164.665	157.000	125.931	112.000	0.014	0.047	
	Lysine	217.102	212.000	198.041	200.000	0.017	0.035	212.304	211.000	194.304	191.000	0.173	0.315	221.346	222.500	201.346	201.000	0.047	0.097	
	Methionine	23.237	22.300	19.587	19.300	0.006	0.017	21.648	21.400	18.663	18.400	0.089	0.198	24.642	22.800	20.404	20.100	0.038	0.082	
	Ornithine	81.678	77.000	68.888	64.400	0.010	0.025	77.026	73.600	69.122	66.500	0.355	0.528	85.792	80.900	68.681	63.400	0.015	0.047	
	Phenylalanine	83.994	79.200	64.155	62.500	0.000	0.000	83.117	76.800	64.057	62.200	0.000	0.009	84.769	81.850	64.242	63.350	0.000	0.002	
	Proline	216.673	215.000	183.892	177.000	0.002	0.006	230.435	234.000	194.043	177.000	0.023	0.081	204.500	193.500	174.912	182.000	0.030	0.072	
	Serine	115.745	115.000	136.159	135.000	0.000	0.000	112.100	113.000	140.761	137.000	0.000	0.009	118.969	118.000	132.088	134.000	0.021	0.059	
	Threonine	122.131	120.000	109.312	100.000	0.015	0.032	111.948	106.000	101.030	93.300	0.144	0.283	131.138	129.500	116.638	106.500	0.064	0.121	
	Tryptophan	61.243	61.400	50.671	50.200	0.000	0.001	56.613	53.100	45.217	45.500	0.003	0.025	65.338	67.850	55.496	53.900	0.016	0.050	
	Tyrosine	77.851	74.200	63.522	60.400	0.001	0.003	74.004	69.100	60.265	56.400	0.029	0.088	81.254	78.250	66.404	63.650	0.014	0.047	
	Valine	249.184	237.000	195.939	188.000	0.000	0.000	243.913	233.000	194.043	188.000	0.006	0.032	253.846	254.500	197.615	189.500	0.001	0.008	
Biogenic Amines	Asymmetric dimethylarginine (ADMA)	0.542	0.563	0.522	0.487	0.346	0.413	0.535	0.563	0.539	0.487	0.721	0.849	0.547	0.563	0.507	0.487	0.089	0.154	Biogenic Amines
	alpha-Aminoadipic acid	0.924	0.664	0.506	0.400	0.011	0.026	0.992	0.638	0.515	0.300	0.108	0.231	0.865	0.821	0.499	0.400	0.038	0.082	
	cis-4-Hydroxyproline	0.073	0.100	0.071	0.100	0.962	0.978	0.065	0.100	0.091	0.100	0.242	0.403	0.081	0.100	0.054	0.000	0.140	0.219	
	Creatinine	91.678	76.500	92.771	68.800	0.145	0.208	104.248	69.100	113.557	67.000	0.855	0.941	80.558	78.050	74.385	71.100	0.080	0.142	
	Dihydroxyphenylalanine (DOPA)	0.192	0.200	0.180	0.200	0.351	0.415	0.196	0.200	0.178	0.200	0.463	0.614	0.188	0.200	0.181	0.200	0.817	0.859	
	Dopamine	0.124	0.100	0.147	0.100	0.378	0.442	0.130	0.100	0.135	0.100	1.000	1.000	0.119	0.100	0.158	0.150	0.199	0.290	
	Histamine	0.173	0.200	0.171	0.200	0.835	0.892	0.170	0.200	0.174	0.200	1.000	1.000	0.177	0.200	0.169	0.200	0.791	0.841	
	Kynurenine	2.929	2.680	2.408	2.310	0.000	0.002	3.194	2.690	2.547	2.410	0.003	0.025	2.694	2.510	2.286	2.310	0.028	0.069	
	Methionine sulfoxide	0.573	0.561	0.542	0.422	0.027	0.049	0.608	0.654	0.675	0.422	0.248	0.404	0.543	0.561	0.425	0.422	0.043	0.090	
	Nitrotyrosine	0.027	0.000	0.029	0.000	0.835	0.892	0.030	0.000	0.026	0.000	1.000	1.000	0.023	0.000	0.031	0.000	0.791	0.841	
	Putrescine	0.127	0.100	0.122	0.100	0.847	0.899	0.117	0.100	0.104	0.100	0.250	0.404	0.135	0.100	0.138	0.100	0.809	0.856	
	Sarcosine	1.165	1.150	0.865	0.839	0.000	0.000	1.155	0.973	0.863	0.875	0.002	0.023	1.173	1.260	0.867	0.812	0.000	0.003	
	Symmetric dimethylarginine (SDMA)	0.452	0.397	0.548	0.496	0.000	0.000	0.507	0.397	0.623	0.496	0.001	0.016	0.403	0.397	0.481	0.496	0.004	0.017	
	Serotonin	0.533	0.442	0.705	0.481	0.038	0.065	0.597	0.481	0.683	0.481	0.230	0.385	0.477	0.385	0.724	0.517	0.084	0.148	
	Spermidine	0.169	0.167	0.173	0.167	0.850	0.899	0.163	0.167	0.175	0.167	0.762	0.886	0.175	0.167	0.172	0.167	0.615	0.686	
	Spermine	0.131	0.100	0.110	0.100	0.012	0.028	0.122	0.100	0.109	0.100	0.453	0.614	0.138	0.100	0.112	0.100	0.039	0.084	
	trans-4-Hydroxyproline	12.209	10.600	11.447	10.800	0.881	0.912	13.143	9.210	11.917	10.800	0.879	0.956	11.383	11.100	11.031	10.850	0.901	0.921	
	Taurine	97.722	98.900	99.098	96.500	0.882	0.912	89.052	86.900	95.904	93.200	0.709	0.847	105.392	101.800	101.923	101.000	0.578	0.663	
Glycerophospholipids	Lyso-phosphatidylcholine C14:0	4.818	4.800	4.372	4.430	0.019	0.038	4.759	4.670	4.453	4.500	0.346	0.519	4.870	4.855	4.302	4.365	0.015	0.047	Glycerophospholipids
	Lyso-phosphatidylcholine C16:0	61.210	59.800	55.657	54.200	0.080	0.122	57.970	55.500	55.900	55.400	0.465	0.614	64.077	67.100	55.442	52.950	0.059	0.116	
	Lyso-phosphatidylcholine C16:1	2.310	2.190	1.689	1.650	0.000	0.000	2.300	2.100	1.784	1.700	0.023	0.081	2.318	2.250	1.605	1.590	0.000	0.003	
	Lyso-phosphatidylcholine C17:0	1.145	1.080	1.021	0.928	0.183	0.246	1.085	1.070	1.024	0.899	0.659	0.801	1.199	1.150	1.019	0.933	0.155	0.240	
	Lyso-phosphatidylcholine C18:0	18.376	17.200	13.905	13.300	0.000	0.001	16.796	15.500	13.776	13.100	0.073	0.173	19.773	19.300	14.019	13.800	0.000	0.003	
	Lyso-phosphatidylcholine C18:1	14.335	13.200	13.220	12.700	0.381	0.442	14.455	12.700	13.654	12.500	0.783	0.889	14.229	14.000	12.836	12.750	0.340	0.445	
	Lyso-phosphatidylcholine C18:2	19.939	18.600	15.085	13.100	0.000	0.001	18.183	18.000	13.346	12.800	0.002	0.021	21.492	20.300	16.624	14.850	0.017	0.051	
	Lyso-phosphatidylcholine C20:3	1.861	1.750	1.161	1.110	0.000	0.000	1.777	1.680	1.137	1.080	0.001	0.012	1.936	1.850	1.182	1.140	0.000	0.001	
	Lyso-phosphatidylcholine C20:4	4.581	4.350	4.528	4.590	0.762	0.833	4.680	3.950	4.651	4.450	0.927	0.980	4.493	4.475	4.419	4.775	0.760	0.818	
	Lyso-phosphatidylcholine C24:0	0.287	0.259	0.226	0.201	0.012	0.026	0.277	0.242	0.235	0.178	0.294	0.463	0.297	0.266	0.218	0.208	0.011	0.043	
	Lyso-phosphatidylcholine C26:0	0.644	0.475	0.414	0.342	0.006	0.017	0.629	0.474	0.448	0.317	0.110	0.231	0.658	0.506	0.385	0.377	0.021	0.058	
	Lyso-phosphatidylcholine C26:1	0.289	0.223	0.262	0.207	0.601	0.674	0.304	0.239	0.291	0.183	0.820	0.918	0.275	0.220	0.237	0.218	0.595	0.669	
	Lyso-phosphatidylcholine C28:0	0.464	0.363	0.288	0.273	0.000	0.002	0.448	0.363	0.301	0.269	0.045	0.119	0.479	0.371	0.276	0.277	0.002	0.011	
	Lyso-phosphatidylcholine C28:1	0.520	0.465	0.406	0.357	0.009	0.023	0.520	0.492	0.439	0.319	0.191	0.335	0.520	0.441	0.376	0.358	0.022	0.060	
	Diacyl-phosphatidylcholine C24:0	0.267	0.219	0.217	0.169	0.149	0.212	0.275	0.208	0.237	0.163	0.465	0.614	0.259	0.220	0.200	0.176	0.190	0.281	
	Diacyl-phosphatidylcholine C26:0	1.381	1.170	1.129	0.859	0.200	0.264	1.330	1.110	1.195	0.845	0.543	0.701	1.426	1.210	1.071	1.051	0.241	0.335	
	Diacyl-phosphatidylcholine C28:1	2.635	2.650	2.069	1.970	0.000	0.000	2.592	2.500	2.125	1.970	0.005	0.030	2.673	2.775	2.020	1.930	0.001	0.005	
	Diacyl-phosphatidylcholine C30:0	3.420	3.140	2.549	2.440	0.000	0.001	3.299	3.070	2.650	2.460	0.042	0.114	3.527	3.495	2.460	2.360	0.001	0.009	
	Diacyl-phosphatidylcholine C32:0	12.158	12.300	12.843	12.100	0.161	0.225	12.217	12.800	13.361	13.500	0.080	0.184	12.105	12.250	12.384	11.850	0.911	0.925	
	Diacyl-phosphatidylcholine C32:1	18.272	16.500	10.839	9.880	0.000	0.000	18.515	16.600	11.522	10.200	0.001	0.015	18.057	16.000	10.235	9.360	0.000	0.002	
	Diacyl-phosphatidylcholine C32:2	3.623	3.650	1.840	1.710	0.000	0.000	3.384	3.080	1.779	1.250	0.000	0.009	3.833	3.950	1.895	1.910	0.000	0.000	
	Diacyl-phosphatidylcholine C32:3	0.384	0.374	0.340	0.332	0.017	0.035	0.359	0.338	0.363	0.332	0.715	0.847	0.406	0.413	0.320	0.330	0.003	0.015	
	Diacyl-phosphatidylcholine C34:1	118.027	119.000	117.418	118.000	0.820	0.892	117.974	121.000	119.052	121.000	0.603	0.748	118.073	119.000	115.973	117.000	0.438	0.538	
	Diacyl-phosphatidylcholine C34:2	129.710	131.000	125.388	130.000	0.380	0.442	128.913	132.000	123.478	130.000	0.964	1.000	130.415	130.000	127.077	127.000	0.402	0.503	
	Diacyl-phosphatidylcholine C34:3	8.904	8.980	5.151	4.930	0.000	0.000	8.431	7.920	5.144	4.450	0.000	0.009	9.323	9.335	5.158	5.245	0.000	0.000	
	Diacyl-phosphatidylcholine C34:4	1.039	0.975	0.516	0.427	0.000	0.000	0.997	0.932	0.513	0.418	0.000	0.009	1.077	1.004	0.519	0.431	0.000	0.000	
	Diacyl-phosphatidylcholine C36:0	1.671	1.500	2.343	1.960	0.000	0.002	1.709	1.500	2.368	1.960	0.013	0.056	1.637	1.480	2.320	1.950	0.013	0.045	
	Diacyl-phosphatidylcholine C36:1	51.157	45.900	32.820	31.900	0.000	0.000	47.891	44.600	33.052	28.600	0.001	0.012	54.046	54.000	32.615	32.800	0.000	0.000	
	Diacyl-phosphatidylcholine C36:2	112.127	113.000	105.104	109.000	0.016	0.034	111.513	113.000	104.865	109.000	0.107	0.231	112.669	113.500	105.315	109.500	0.078	0.141	
	Diacyl-phosphatidylcholine C36:3	86.508	91.400	73.839	75.300	0.000	0.001	82.996	88.800	74.809	76.200	0.029	0.088	89.615	94.600	72.981	73.050	0.001	0.009	
	Diacyl-phosphatidylcholine C36:4	111.539	113.000	113.008	115.000	0.861	0.905	111.361	114.000	114.104	115.000	0.408	0.583	111.696	111.500	112.038	113.000	0.583	0.663	
	Diacyl-phosphatidylcholine C36:5	14.414	13.800	9.392	7.660	0.000	0.000	14.827	15.000	9.871	7.360	0.001	0.016	14.049	12.950	8.967	7.950	0.000	0.004	
	Diacyl-phosphatidylcholine C36:6	0.365	0.358	0.233	0.178	0.000	0.000	0.361	0.328	0.244	0.172	0.001	0.015	0.369	0.369	0.222	0.208	0.000	0.003	
	Diacyl-phosphatidylcholine C38:0	1.962	1.890	2.042	1.880	0.944	0.965	1.854	1.840	2.033	1.850	0.553	0.709	2.057	1.940	2.050	1.885	0.657	0.725	
	Diacyl-phosphatidylcholine C38:3	46.729	44.200	32.367	30.600	0.000	0.000	43.743	42.900	32.974	29.200	0.004	0.025	49.369	47.200	31.831	31.300	0.000	0.000	
	Diacyl-phosphatidylcholine C38:4	82.176	84.200	80.382	76.900	0.409	0.473	82.800	84.000	82.678	76.600	0.784	0.889	81.623	84.400	78.350	77.100	0.380	0.484	
	Diacyl-phosphatidylcholine C38:5	34.584	33.100	32.649	30.700	0.121	0.174	35.235	34.300	35.600	31.200	0.784	0.889	34.008	32.700	30.038	30.400	0.066	0.122	
	Diacyl-phosphatidylcholine C38:6	45.108	45.300	52.969	49.100	0.037	0.065	44.274	41.700	54.265	52.900	0.033	0.098	45.846	47.150	51.823	47.200	0.629	0.698	
	Diacyl-phosphatidylcholine C40:1	0.307	0.294	0.301	0.302	0.198	0.263	0.297	0.290	0.297	0.291	0.648	0.795	0.316	0.303	0.305	0.309	0.239	0.335	
	Diacyl-phosphatidylcholine C40:2	0.324	0.311	0.294	0.292	0.008	0.021	0.316	0.303	0.290	0.266	0.107	0.231	0.331	0.322	0.298	0.294	0.035	0.080	
	Diacyl-phosphatidylcholine C40:3	0.507	0.497	0.478	0.473	0.151	0.213	0.512	0.503	0.493	0.471	0.465	0.614	0.503	0.496	0.466	0.476	0.188	0.280	
	Diacyl-phosphatidylcholine C40:4	3.462	3.440	2.708	2.690	0.000	0.001	3.282	3.220	2.855	2.730	0.042	0.114	3.622	3.485	2.579	2.630	0.001	0.005	
	Diacyl-phosphatidylcholine C40:5	7.693	7.250	6.162	5.860	0.000	0.001	7.599	7.240	6.649	6.060	0.153	0.293	7.775	8.025	5.730	5.590	0.000	0.003	
	Diacyl-phosphatidylcholine C40:6	18.664	18.600	17.898	16.000	0.231	0.292	17.632	17.000	17.886	17.100	0.927	0.980	19.577	19.350	17.910	15.700	0.137	0.217	
	Diacyl-phosphatidylcholine C42:0	0.412	0.405	0.464	0.437	0.021	0.040	0.399	0.367	0.457	0.449	0.045	0.119	0.424	0.421	0.471	0.434	0.152	0.236	
	Diacyl-phosphatidylcholine C42:1	0.202	0.192	0.220	0.212	0.238	0.298	0.195	0.192	0.215	0.212	0.323	0.496	0.208	0.194	0.224	0.211	0.519	0.618	
	Diacyl-phosphatidylcholine C42:2	0.188	0.183	0.172	0.163	0.015	0.032	0.174	0.169	0.167	0.161	0.420	0.588	0.200	0.186	0.176	0.166	0.019	0.055	
	Diacyl-phosphatidylcholine C42:4	0.179	0.177	0.162	0.158	0.008	0.022	0.172	0.175	0.161	0.158	0.166	0.306	0.185	0.185	0.163	0.162	0.026	0.066	
	Diacyl-phosphatidylcholine C42:5	0.276	0.275	0.265	0.257	0.205	0.266	0.278	0.279	0.274	0.277	0.891	0.958	0.275	0.275	0.257	0.245	0.131	0.212	
	Diacyl-phosphatidylcholine C42:6	0.446	0.441	0.376	0.365	0.001	0.003	0.439	0.430	0.395	0.372	0.176	0.315	0.452	0.453	0.359	0.359	0.001	0.007	
	Acyl-alkyl-phosphatidylcholine C30:0	0.286	0.272	0.229	0.207	0.000	0.002	0.266	0.256	0.230	0.221	0.060	0.150	0.304	0.304	0.228	0.207	0.002	0.009	
	Acyl-alkyl-phosphatidylcholine C30:1	0.291	0.240	0.229	0.201	0.044	0.073	0.276	0.230	0.254	0.204	0.370	0.546	0.304	0.249	0.206	0.193	0.041	0.086	
	Acyl-alkyl-phosphatidylcholine C30:2	0.133	0.126	0.115	0.115	0.017	0.035	0.130	0.124	0.122	0.132	0.429	0.595	0.136	0.134	0.110	0.107	0.013	0.045	
	Acyl-alkyl-phosphatidylcholine C32:1	2.101	2.060	2.333	2.200	0.023	0.043	1.967	1.960	2.290	2.330	0.023	0.081	2.219	2.165	2.370	2.145	0.377	0.483	
	Acyl-alkyl-phosphatidylcholine C32:2	0.588	0.593	0.615	0.580	0.420	0.483	0.555	0.543	0.616	0.565	0.224	0.378	0.616	0.646	0.615	0.609	0.915	0.925	
	Acyl-alkyl-phosphatidylcholine C34:0	1.139	1.130	0.998	0.938	0.022	0.041	1.098	1.040	1.027	0.892	0.144	0.283	1.175	1.140	0.973	0.983	0.060	0.117	
	Acyl-alkyl-phosphatidylcholine C34:1	7.345	7.130	7.600	7.270	0.306	0.369	7.438	6.440	7.919	7.490	0.201	0.350	7.262	7.220	7.317	7.135	0.856	0.886	
	Acyl-alkyl-phosphatidylcholine C34:2	7.231	7.070	6.588	6.030	0.012	0.027	6.635	6.280	6.475	5.790	0.078	0.181	7.758	7.550	6.688	6.335	0.049	0.101	
	Acyl-alkyl-phosphatidylcholine C34:3	4.437	4.300	4.848	4.560	0.275	0.336	4.047	3.970	4.473	4.340	0.685	0.827	4.782	4.995	5.180	5.070	0.291	0.399	
	Acyl-alkyl-phosphatidylcholine C36:0	0.604	0.588	0.605	0.579	0.432	0.493	0.595	0.579	0.616	0.579	0.438	0.603	0.611	0.595	0.596	0.582	0.741	0.802	
	Acyl-alkyl-phosphatidylcholine C36:1	16.130	15.800	13.762	13.300	0.002	0.007	15.692	15.200	14.259	13.300	0.136	0.272	16.518	17.050	13.323	13.200	0.005	0.020	
	Acyl-alkyl-phosphatidylcholine C36:2	9.145	9.070	8.070	7.730	0.004	0.012	8.566	8.540	7.968	7.200	0.055	0.141	9.657	9.465	8.160	7.900	0.035	0.080	
	Acyl-alkyl-phosphatidylcholine C36:3	4.537	4.540	3.734	3.660	0.000	0.001	4.289	4.270	3.634	3.400	0.005	0.030	4.757	5.035	3.822	3.820	0.005	0.020	
	Acyl-alkyl-phosphatidylcholine C36:4	11.070	10.600	10.608	10.100	0.171	0.234	10.523	10.600	10.212	9.470	0.412	0.584	11.553	10.750	10.958	10.400	0.250	0.344	
	Acyl-alkyl-phosphatidylcholine C36:5	7.192	7.010	8.341	7.770	0.035	0.063	6.800	6.610	7.918	6.710	0.287	0.456	7.539	7.125	8.716	8.400	0.063	0.121	
	Acyl-alkyl-phosphatidylcholine C38:0	0.787	0.754	0.628	0.527	0.000	0.002	0.760	0.712	0.632	0.490	0.031	0.092	0.812	0.863	0.624	0.625	0.003	0.016	
	Acyl-alkyl-phosphatidylcholine C38:1	1.518	1.570	1.223	1.170	0.002	0.006	1.480	1.550	1.202	1.140	0.028	0.088	1.552	1.575	1.243	1.170	0.029	0.071	
	Acyl-alkyl-phosphatidylcholine C38:2	2.011	1.970	1.605	1.500	0.001	0.002	1.866	1.830	1.565	1.420	0.023	0.081	2.139	2.030	1.641	1.575	0.010	0.039	
	Acyl-alkyl-phosphatidylcholine C38:3	5.196	5.260	4.262	3.960	0.000	0.001	4.985	4.840	4.327	3.710	0.031	0.092	5.383	5.275	4.205	4.000	0.001	0.006	
	Acyl-alkyl-phosphatidylcholine C38:4	8.149	8.060	8.262	7.790	0.574	0.647	7.934	7.620	8.546	8.020	0.879	0.956	8.339	8.350	8.012	7.780	0.398	0.502	
	Acyl-alkyl-phosphatidylcholine C38:5	10.264	10.000	11.521	10.900	0.025	0.045	10.113	9.790	11.709	11.200	0.024	0.082	10.397	10.450	11.354	10.750	0.331	0.439	
	Acyl-alkyl-phosphatidylcholine C38:6	3.677	3.520	3.840	3.570	0.933	0.958	3.476	3.420	3.740	3.340	0.738	0.863	3.854	3.845	3.928	3.665	0.847	0.880	
	Acyl-alkyl-phosphatidylcholine C40:1	1.024	1.010	0.937	0.900	0.070	0.110	0.975	0.908	0.960	0.900	0.773	0.889	1.068	1.065	0.918	0.900	0.031	0.073	
	Acyl-alkyl-phosphatidylcholine C40:2	1.873	1.810	2.086	2.090	0.072	0.111	1.800	1.780	2.122	2.120	0.020	0.076	1.938	1.920	2.055	2.025	0.592	0.669	
	Acyl-alkyl-phosphatidylcholine C40:3	1.355	1.330	1.303	1.250	0.223	0.286	1.282	1.250	1.305	1.250	0.831	0.926	1.420	1.415	1.301	1.280	0.127	0.209	
	Acyl-alkyl-phosphatidylcholine C40:4	1.555	1.540	1.505	1.470	0.166	0.229	1.506	1.510	1.505	1.420	0.649	0.795	1.599	1.610	1.505	1.490	0.134	0.214	
	Acyl-alkyl-phosphatidylcholine C40:5	2.874	2.840	3.409	3.310	0.001	0.003	2.814	2.580	3.520	3.390	0.004	0.027	2.927	3.015	3.310	3.305	0.067	0.123	
	Acyl-alkyl-phosphatidylcholine C40:6	2.517	2.420	2.830	2.690	0.061	0.098	2.433	2.330	2.914	2.690	0.027	0.088	2.592	2.670	2.756	2.680	0.561	0.650	
	Acyl-alkyl-phosphatidylcholine C42:0	0.587	0.584	0.596	0.583	0.874	0.912	0.598	0.584	0.613	0.584	0.574	0.726	0.578	0.582	0.581	0.581	0.519	0.618	
	Acyl-alkyl-phosphatidylcholine C42:1	0.405	0.381	0.371	0.348	0.114	0.166	0.395	0.361	0.396	0.404	0.910	0.972	0.413	0.383	0.349	0.342	0.026	0.066	
	Acyl-alkyl-phosphatidylcholine C42:2	0.469	0.449	0.412	0.396	0.003	0.008	0.445	0.438	0.426	0.403	0.346	0.519	0.490	0.476	0.400	0.385	0.001	0.009	
	Acyl-alkyl-phosphatidylcholine C42:3	0.676	0.653	0.643	0.589	0.085	0.127	0.627	0.599	0.618	0.574	0.523	0.685	0.720	0.703	0.665	0.590	0.103	0.176	
	Acyl-alkyl-phosphatidylcholine C42:4	0.621	0.620	0.579	0.533	0.052	0.084	0.598	0.620	0.559	0.521	0.153	0.293	0.642	0.644	0.598	0.571	0.208	0.300	
	Acyl-alkyl-phosphatidylcholine C42:5	1.791	1.750	2.113	2.100	0.001	0.002	1.786	1.670	2.133	2.080	0.014	0.058	1.795	1.800	2.095	2.200	0.013	0.045	
	Acyl-alkyl-phosphatidylcholine C44:3	0.145	0.131	0.123	0.112	0.016	0.034	0.139	0.125	0.122	0.109	0.223	0.378	0.151	0.136	0.124	0.124	0.033	0.077	
	Acyl-alkyl-phosphatidylcholine C44:4	0.341	0.321	0.314	0.305	0.023	0.043	0.315	0.316	0.301	0.305	0.162	0.301	0.364	0.334	0.325	0.311	0.074	0.135	
	Acyl-alkyl-phosphatidylcholine C44:5	1.218	1.200	1.514	1.400	0.000	0.001	1.260	1.190	1.494	1.400	0.017	0.070	1.181	1.205	1.532	1.400	0.004	0.018	
	Acyl-alkyl-phosphatidylcholine C44:6	0.752	0.721	0.845	0.806	0.012	0.026	0.737	0.720	0.815	0.768	0.157	0.298	0.766	0.730	0.872	0.849	0.039	0.084	
Sphingomyelins	Sphingomyelin ((OH) C14:1)	5.378	5.640	5.653	5.380	0.615	0.685	5.187	5.640	5.740	5.320	0.543	0.701	5.547	5.680	5.576	5.485	0.880	0.905	Sphingomyelins
	Sphingomyelin ((OH) C16:1)	3.179	3.000	3.705	3.530	0.016	0.034	3.044	2.980	3.844	3.510	0.019	0.074	3.298	3.055	3.582	3.620	0.337	0.445	
	Sphingomyelin ((OH) C22:1)	11.208	10.900	7.710	6.750	0.000	0.000	10.321	10.300	7.263	6.420	0.001	0.015	11.993	12.450	8.106	7.400	0.000	0.000	
	Sphingomyelin ((OH) C22:2)	9.366	8.920	8.859	8.260	0.162	0.225	9.012	8.520	8.919	8.070	0.447	0.611	9.679	9.850	8.805	8.460	0.215	0.308	
	Sphingomyelin ((OH) C24:1)	1.151	1.120	0.905	0.852	0.000	0.001	1.049	1.060	0.884	0.817	0.006	0.034	1.241	1.270	0.923	0.895	0.002	0.011	
	Sphingomyelin (C16:0)	97.614	98.700	112.812	110.000	0.004	0.012	95.422	98.700	115.122	115.000	0.011	0.051	99.554	99.150	110.769	102.000	0.106	0.178	
	Sphingomyelin (C16:1)	17.299	17.300	18.624	17.800	0.255	0.316	16.757	17.100	19.259	19.000	0.121	0.244	17.778	17.550	18.063	17.350	1.000	1.000	
	Sphingomyelin (C18:0)	20.594	19.200	27.159	25.900	0.000	0.001	20.391	18.600	29.530	27.200	0.004	0.025	20.773	19.500	25.062	25.150	0.017	0.050	
	Sphingomyelin (C18:1)	12.309	11.500	16.580	15.800	0.000	0.001	12.040	11.000	18.030	17.400	0.004	0.026	12.547	11.800	15.298	14.850	0.022	0.060	
	Sphingomyelin (C20:2)	0.401	0.391	0.519	0.489	0.002	0.007	0.404	0.396	0.582	0.539	0.014	0.058	0.399	0.372	0.463	0.466	0.058	0.116	
	Sphingomyelin (C24:0)	18.418	17.400	14.285	13.500	0.000	0.000	16.530	17.000	13.925	13.200	0.008	0.041	20.088	19.450	14.603	14.600	0.000	0.001	
	Sphingomyelin (C24:1)	47.835	48.200	60.314	59.400	0.000	0.001	46.604	46.500	63.165	59.400	0.001	0.015	48.923	49.100	57.792	59.550	0.025	0.066	
	Sphingomyelin (C26:0)	0.148	0.142	0.141	0.135	0.203	0.266	0.140	0.136	0.147	0.135	0.964	1.000	0.155	0.150	0.137	0.131	0.109	0.180	
	Sphingomyelin (C26:1)	0.323	0.309	0.401	0.377	0.002	0.007	0.306	0.305	0.433	0.384	0.001	0.012	0.338	0.331	0.373	0.360	0.342	0.445	
Sugar	Sugar	5903.490	5100.000	5192.878	4640.000	0.011	0.026	6532.696	6190.000	5166.870	5319.000	0.012	0.053	5346.885	4645.500	5215.885	4517.500	0.237	0.334	Sugar

Supplementary Data 9 – Change in measured serum acylcarnitines, amino acids, biogenic amines, glycerophospholipids and sphingomyelins in participants post Roux-en-Y Gastric Bypass (RYGB) and Vertical Sleeve Gastrectomy (VSG) procedures.

Gut Microbiome Analysis

Supplementary Figure 10 – PCoA comparing gut microbiota analysis in participants with T2D Vs participants without diabetes

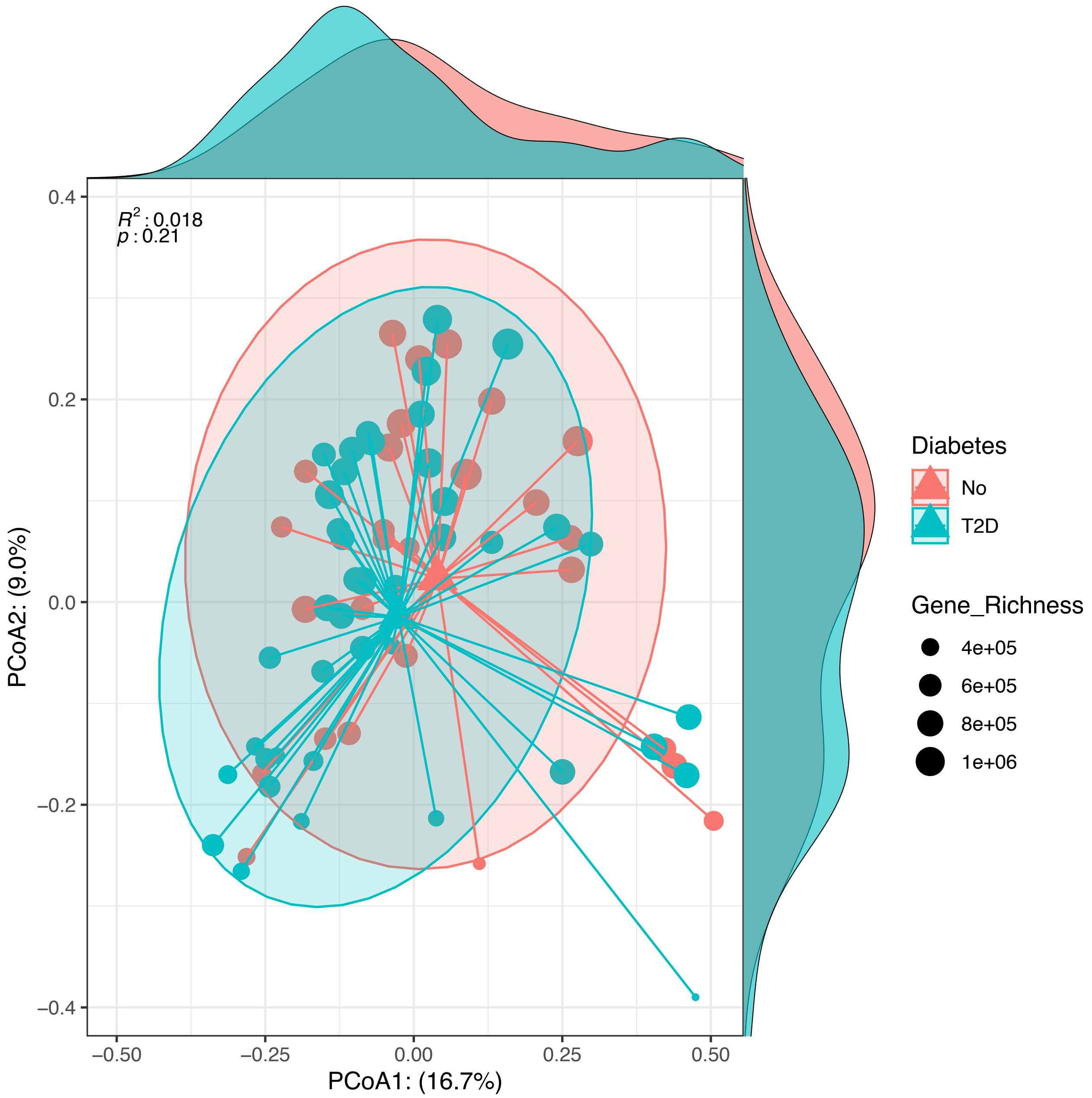

Supplementary Figure 10 – Principal Coordinate Analysis (PCoA) based on Bray-Curtis dissimilarity (β-diversity) of bacterial species, comparing participants with type 2 diabetic (T2D, n=42) versus participants without diabetes (No, n=27). Percent variance explained by each component shown in brackets. Ellipses indicate 90% confidence intervals around samples from each category. Significance of group separation in β-diversity (p) was assessed by permutational multivariate analysis of variance (PERMANOVA).

Supplementary Figure 11 – PCoA comparing gut microbiota analysis in participants with T2D, impaired glucose tolerance and participants without diabetes

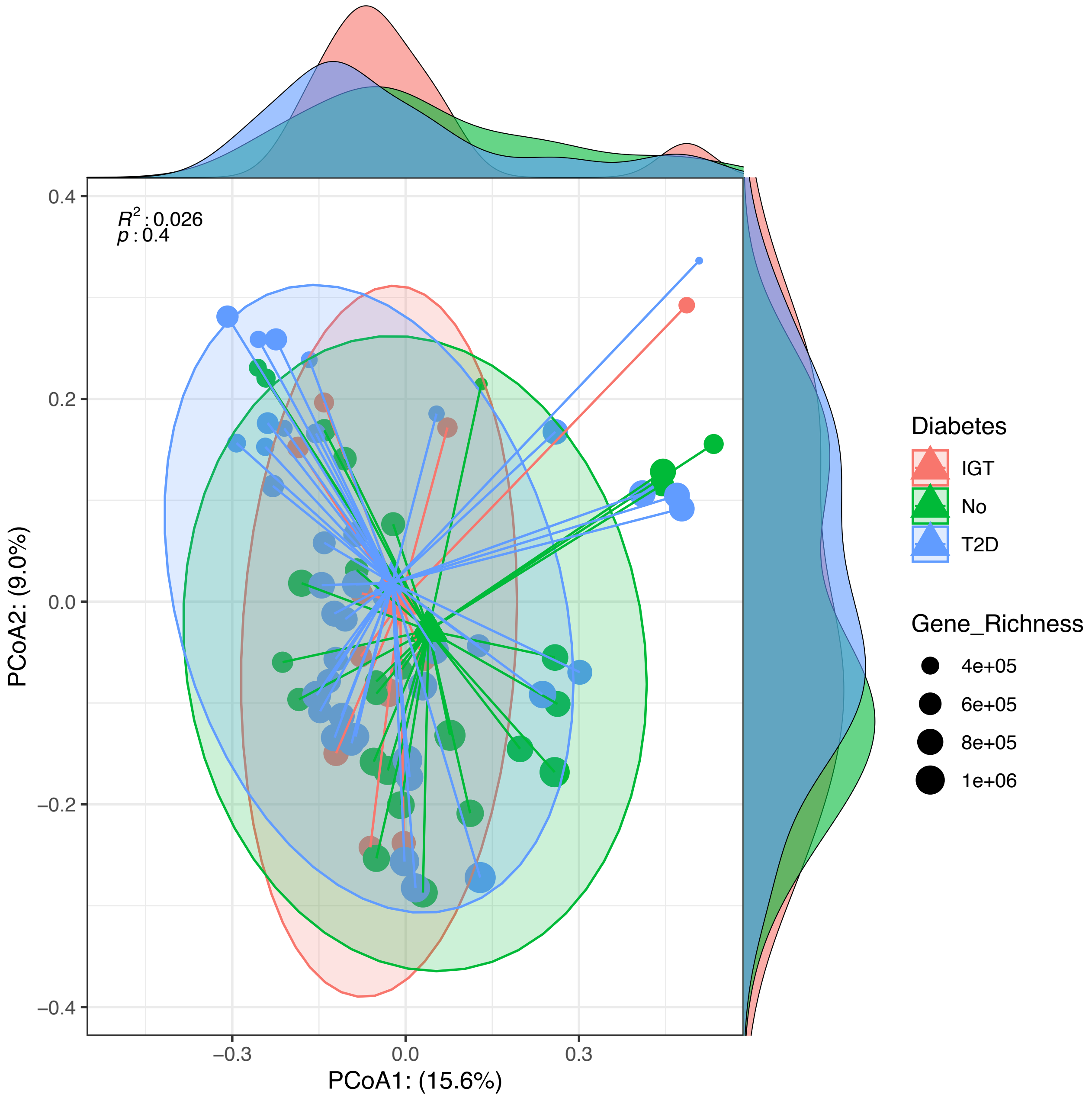

Supplementary Figure 11 – Principal Coordinate Analysis (PCoA) based on Bray-Curtis dissimilarity (β-diversity) of bacterial species, comparing participants with type 2 diabetes (T2D, n=42), impaired glucose tolerance (IGT, n=11) and participants without diabetes (No, n=27). Percent variance explained by each component shown in brackets. Ellipses indicate 90% confidence intervals around samples from each category. Significance of group separation in β-diversity (p) was assessed by permutational multivariate analysis of variance (PERMANOVA).

Supplementary Figure 12 – Phylogenetic tree comparing gut microbiota of participants with T2D to participants without diabetes

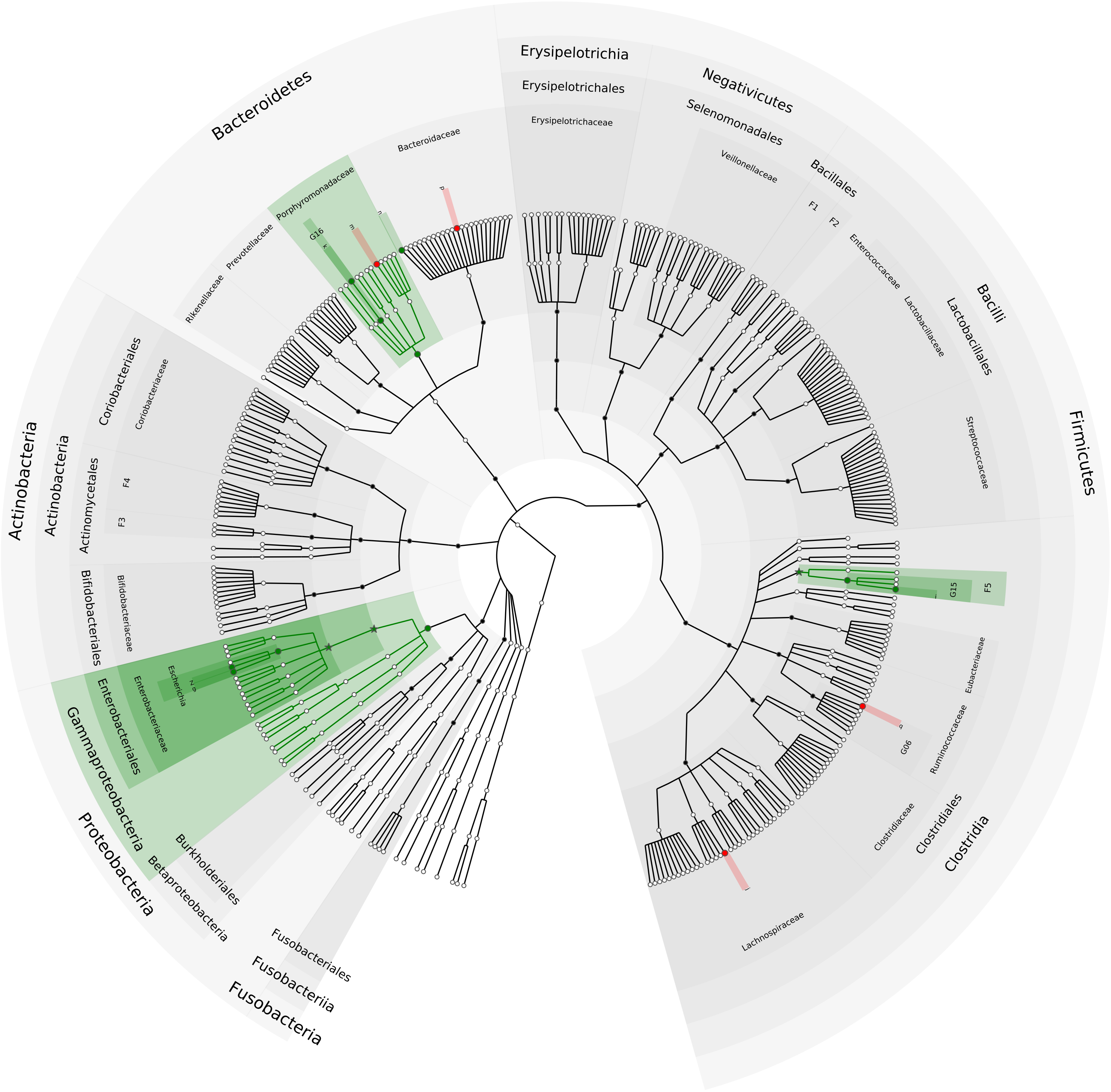

Supplementary Figure 12 – Phylogenetic tree of significant differentially abundant taxa from phyla to species comparing participants with type 2 diabetes (T2D, n=42) to participants without diabetes (n=27). Taxa significantly lower in participants with T2D Vs participants without diabetes are shown in green, taxa significantly higher in participants with T2D Vs participants without diabetes are shown in red. Changes that remain significant after Benjamini-Hochberg multiple testing corrections are denoted with an asterisk (*).

***Species:** Z: Escherichia coli, g: Escherichia unclassified, i: Clostridium bartlettii, j: Ruminococcus torques, k: Barnesiella intestinihominis, m: Parabacteroides unclassified, n: Bacteroides xylanisolvens, p: Bacteroides massiliensis, q: Ruminococcus sp 5.1.39 BFAA. **Genus:** G06: Ruminococcus, G15: Peptostreptococcaceae^, G16: Barnesiella. **Family:** F1: Bacillales^, F2: Carnobacteriaceae, F3: Micrococcaceae, F4: Actinomycetaceae, F5: Peptostreptococcaceae.*

Supplementary Figure 13 – PcoA comparing gut microbiota analysis of participants pre and post RYGB

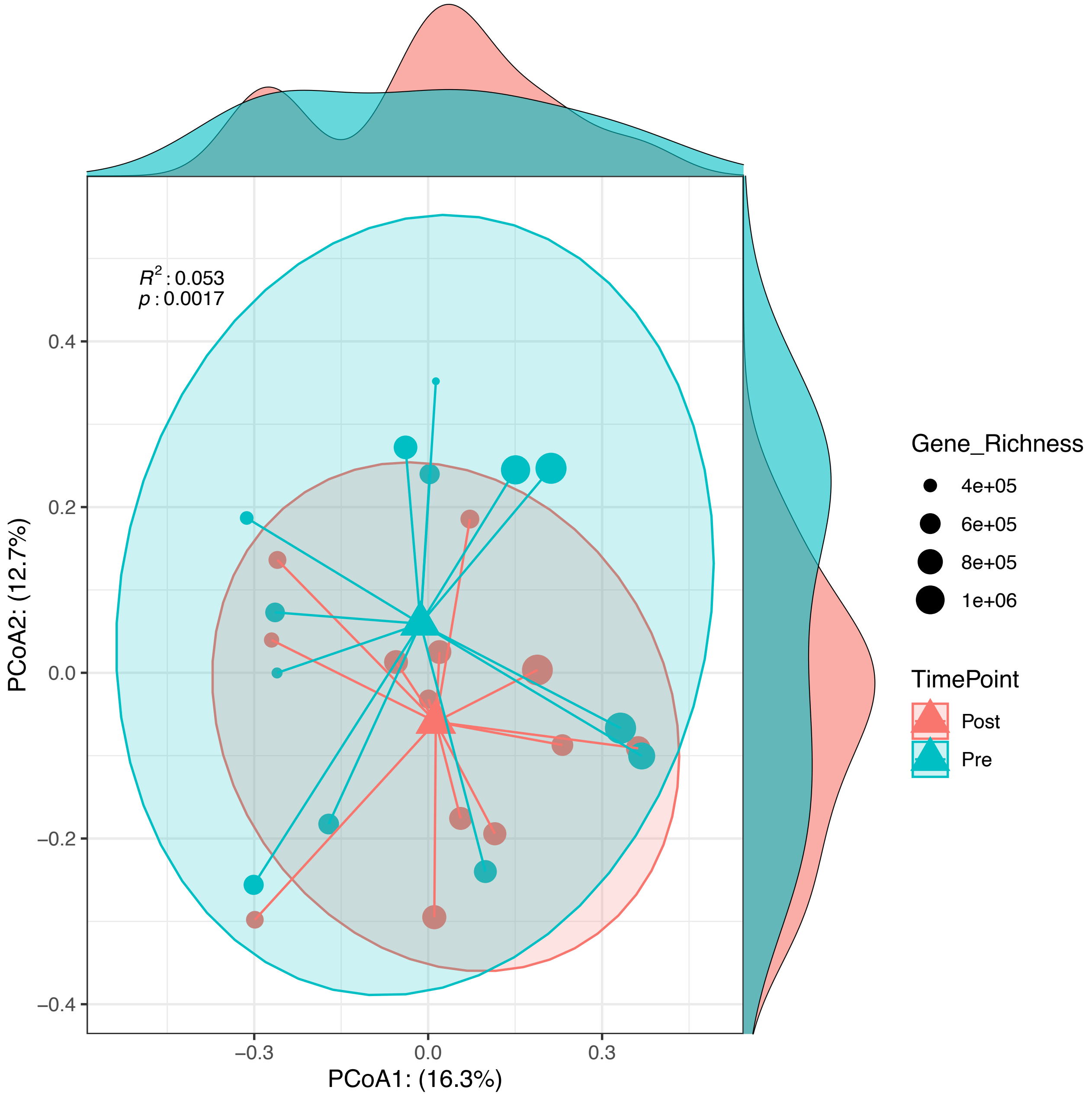

Supplementary Figure 13 – Principal Coordinate Analysis (PCoA) based on Bray-Curtis dissimilarity (β-diversity) of bacterial species, comparing subjects pre and post Roux-en-Y Gastric Bypass (RYGB, n=13). Percent variance explained by each component shown in brackets. Ellipses indicate 90% confidence intervals around samples from each category. Significance of group separation in β-diversity (p) was assessed by permutational multivariate analysis of variance (PERMANOVA).

Supplementary Figure 14 – Phylogenetic tree comparing gut microbiota of participants pre and post RYGB

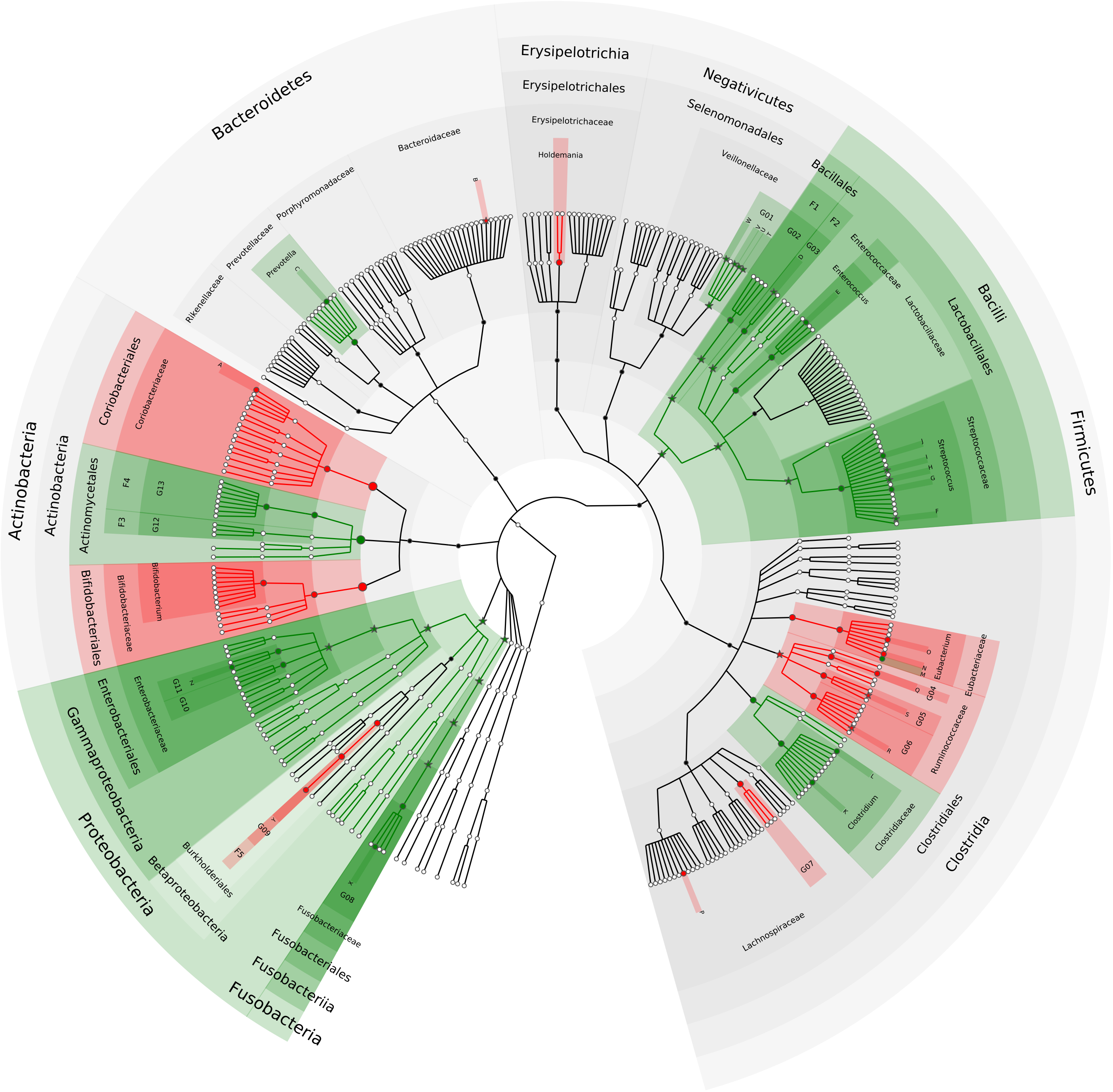

Supplementary Figure 14 - Phylogenetic tree of significant differentially abundant taxa from phyla to species 3-months post Roux-en-Y Gastric Bypass (RYGB, n=13). Taxa significantly increased post-surgery are shown in green, taxa significantly decreased post-surgery are shown in red. Changes that remain significant after Benjamini-Hochberg multiple testing corrections are denoted with an asterisk (*).

Species: *A: Collinsella aerofaciens, B: Bacteroides dorei, C: Prevotella copri, D: Granulicatella unclassified, E: Enterococcus faecalis, F: Streptococcus anginosus, G: Streptococcus infantis, H: Streptococcus mitis oralis pneumoniae, I: Streptococcus parasanguinis, J: Streptococcus salivarius, K: Clostridium hathewayi, L: Clostridium symbiosum, M: Eubacterium eligens, N: Eubacterium hallii, O: Eubacterium rectale, P: Lachnospiraceae bacterium 5.1.63FAA, Q: Faecalibacterium prausnitzii, R: Ruminococcus bromii, S: Subdoligranulum unclassified, T: Veillonella atypica, U: Veillonella dispar, V: Veillonella parvula, W: Veillonella unclassified, X: Fusobacterium nucleatum, Y: Burkholderiales bacterium 1.1.47, Z: Escherichia coli.* ***Genus:*** *G01: Veillonella, G02: Gemella, G03: Granulicatella, G04: Faecalibacterium, G05: Subdoligranulum, G06: Ruminococcus, G07: Anaerostipes, G08: Fusobacterium, G09: Burkholderiales^, G10: Klebsiella, G11: Escherichia, G12: Rothia, G13: Actinomyces.* ***Family:*** *F1: Bacillales^, F2: Carnobacteriaceae, F3: Micrococcaceae, F4: Actinomycetaceae, F5: Burkholderiales^.*

Supplementary Figure 15 - Phylogenetic tree comparing gut microbiota KEGG pathways in participants pre and post RYGB

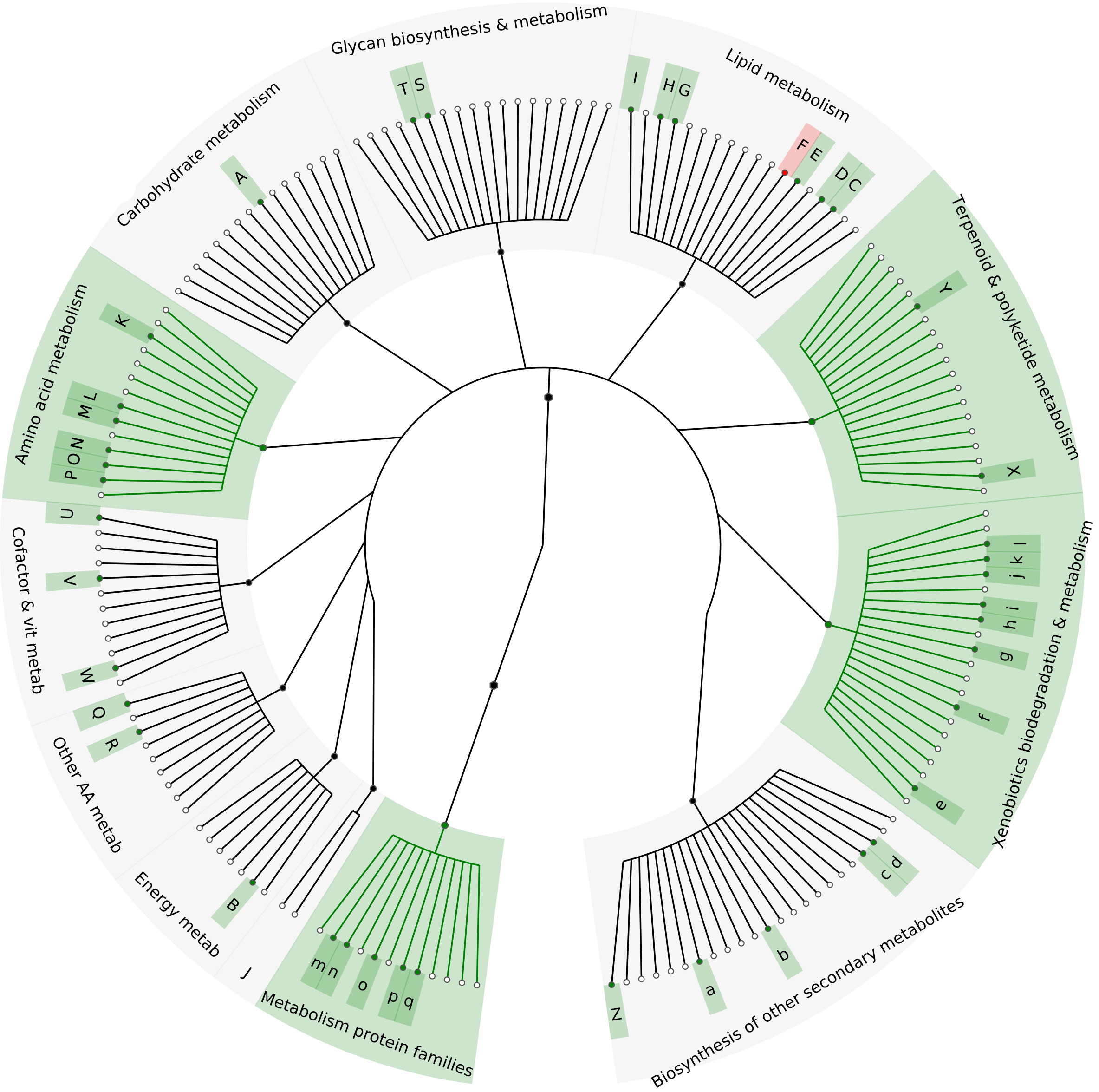

Supplementary Figure 15 – Phylogenetic tree of significant differentially abundant level 2 and level 3 gut microbiota KEGG pathways 3-months post Roux-en-Y Gastric Bypass (RYGB, n=13). KEGG pathways significantly increased post-surgery are shown in green, KEGG pathways significantly decreased post-surgery are shown in red. Changes that remain significant after Benjamini-Hochberg multiple testing corrections are denoted with an asterisk (*).

A: ko00053 Ascorbate and aldarate_metabolism, B: ko00910 Nitrogen metabolism, C: ko00071 Fatty acid degradation, D: ko00072 Synthesis and degradation of ketone bodies, E: ko00100 Steroid biosynthesis, F: ko00120 Primary bile acid biosynthesis, G: ko00591 Linoleic acid metabolism, H: ko00592 alpha Linolenic acid metabolism, I: ko01040 Biosynthesis of unsaturated fatty acids, J: Nucleotide metabolism, K: ko00260 Glycine serine and threonine metabolism, L: ko00310 Lysine degradation, M: ko00330 Arginine and proline metabolism, N: ko00350 Tyrosine metabolism, O: ko00360 Phenylalanine metabolism, P: ko00380 Tryptophan metabolism, Q: ko00410 beta Alanine metabolism, R: ko00440 Phosphonate and phosphinate metabolism, S: ko00537 Glycosylphosphatidylinositol anchored proteins, T: ko00540 Lipopolysaccharide biosynthesis, U: ko00130 Ubiquinone and other terpenoidquinone biosynthesis, V: ko00750 Vitamin B6 metabolism, W: ko00830 Retinol metabolism, X: ko00281 Geraniol degradation, Y: ko01053 Biosynthesis of siderophore group nonribosomal peptides, Z: ko00232 Caffeine metabolism, a: ko00333 Prodigiosin biosyntheses, b: ko00524 Neomycin kanamycin and gentamicin biosynthesis, c: ko00950 Isoquinoline alkaloid biosynthesis, d: ko00960 Tropane piperidine and pyridine alkaloid biosynthesis, e: ko00362 Benzoate degradation, f: ko00623 Toluene degradation, g: ko00627 Aminobenzoate degradation, h: ko00642 Ethylbenzene degradation, i: ko00643 Styrene degradation, j: ko00930 Caprolactam degradation, k: ko00980 Metabolism of xenobiotics by cytochrome P450, l: ko00982 Drug metabolism cytochrome P450, m: ko00199 Cytochrome P450, n: ko01001 Protein kinases, o: ko01003 Glycosyltransferases, p: ko01005 Lipopolysaccharide biosynthesis proteins, q: ko01006 Prenyltransferases.

Supplementary Figure 16 – PcoA comparing gut microbiota analysis of participants pre and post VSG

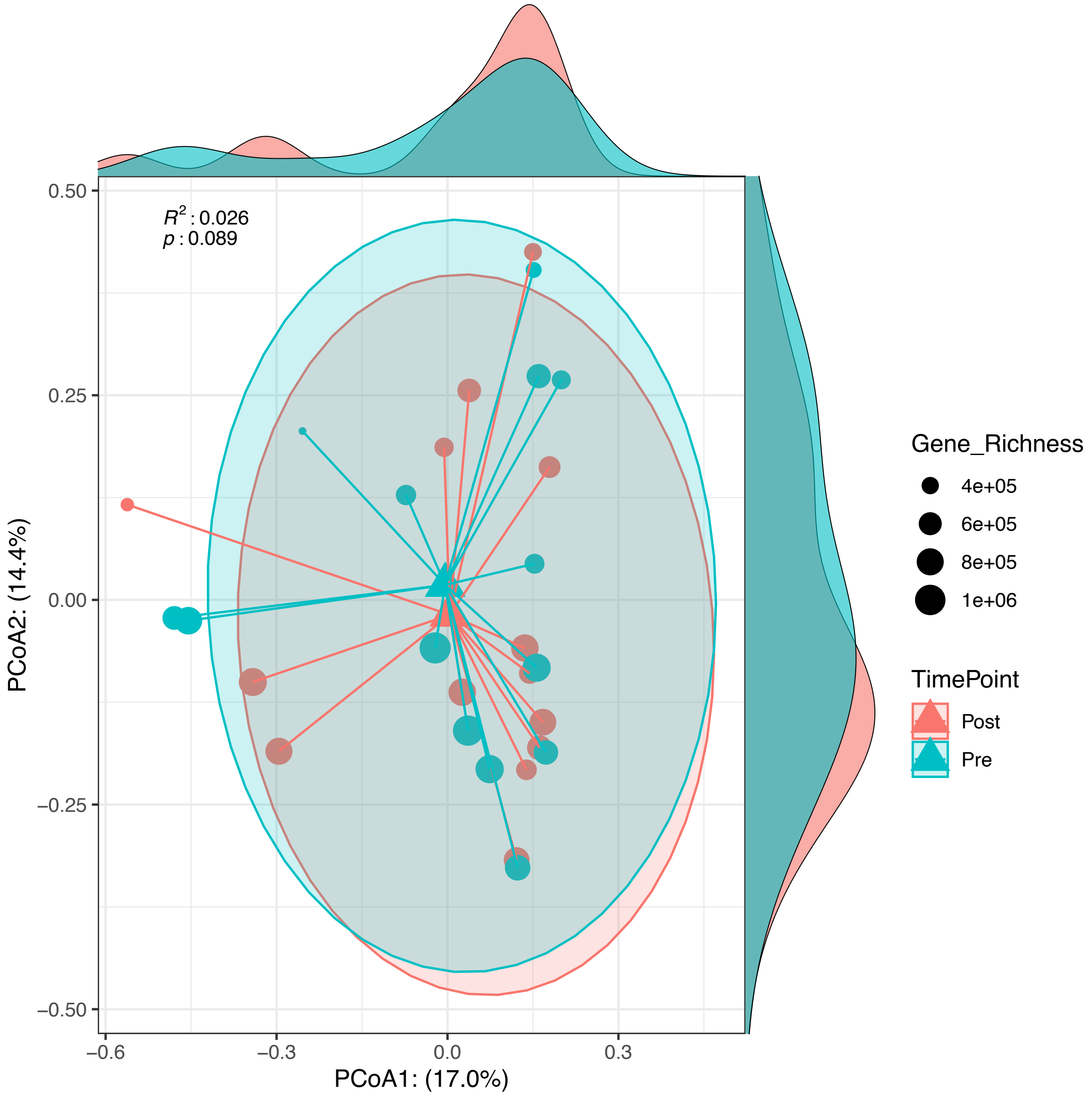

Supplementary Figure 16 – Principal Coordinate Analysis (PCoA) based on Bray-Curtis dissimilarity (β-diversity) of bacterial species, comparing subjects pre and post Vertical Sleeve Gastrectomy (VSG, n=14). Percent variance explained by each component shown in brackets. Ellipses indicate 90% confidence intervals around samples from each category. Significance of group separation in β-diversity (p) was assessed by permutational multivariate analysis of variance (PERMANOVA).

Supplementary Figure 17 – Phylogenetic tree comparing gut microbiota of participants pre and post VSG

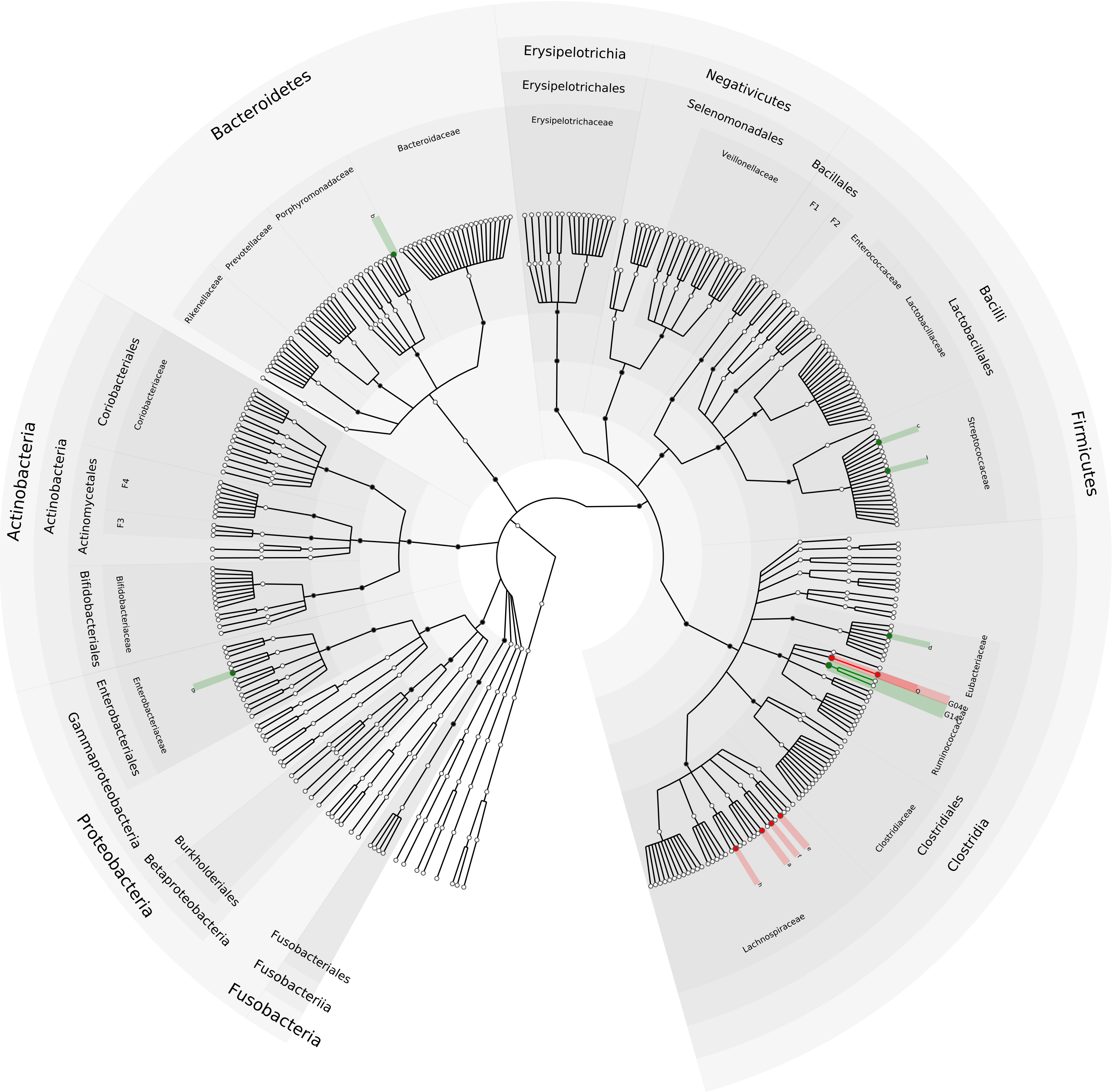

Supplementary Figure 17 – Phylogenetic tree of significant differentially abundant taxa from phyla to species 3-months post Vertical Sleeve Gastrectomy (VSG, n=14). Taxa significantly increased post-surgery are shown in green, taxa significantly decreased post-surgery are shown in red. Changes that remain significant after Benjamini-Hochberg multiple testing corrections are denoted with an asterisk (*).

Species: *I: Streptococcus parasanguinis, Q: Faecalibacterium prausnitzii, a: Roseburia unclassified, b: Parabacteroides distasonis, c: Streptococcus thermophilus, d: Eubacterium siraeum, e: Dorea formicigenerans, f: Anaerostipes hadrus, g: Escherichia unclassified, h: Coprococcus comes.* ***Genus:*** *G04: Faecalibacterium, G14: Anaerotruncus.* ***Family:*** *F1: Bacillales^, F2: Carnobacteriaceae, F3: Micrococcaceae, F4: Actinomycetaceae.*

Integrative Analysis

Supplementary Figure 18 – DIABLO model comparing integrated datasets of participants with and without T2D

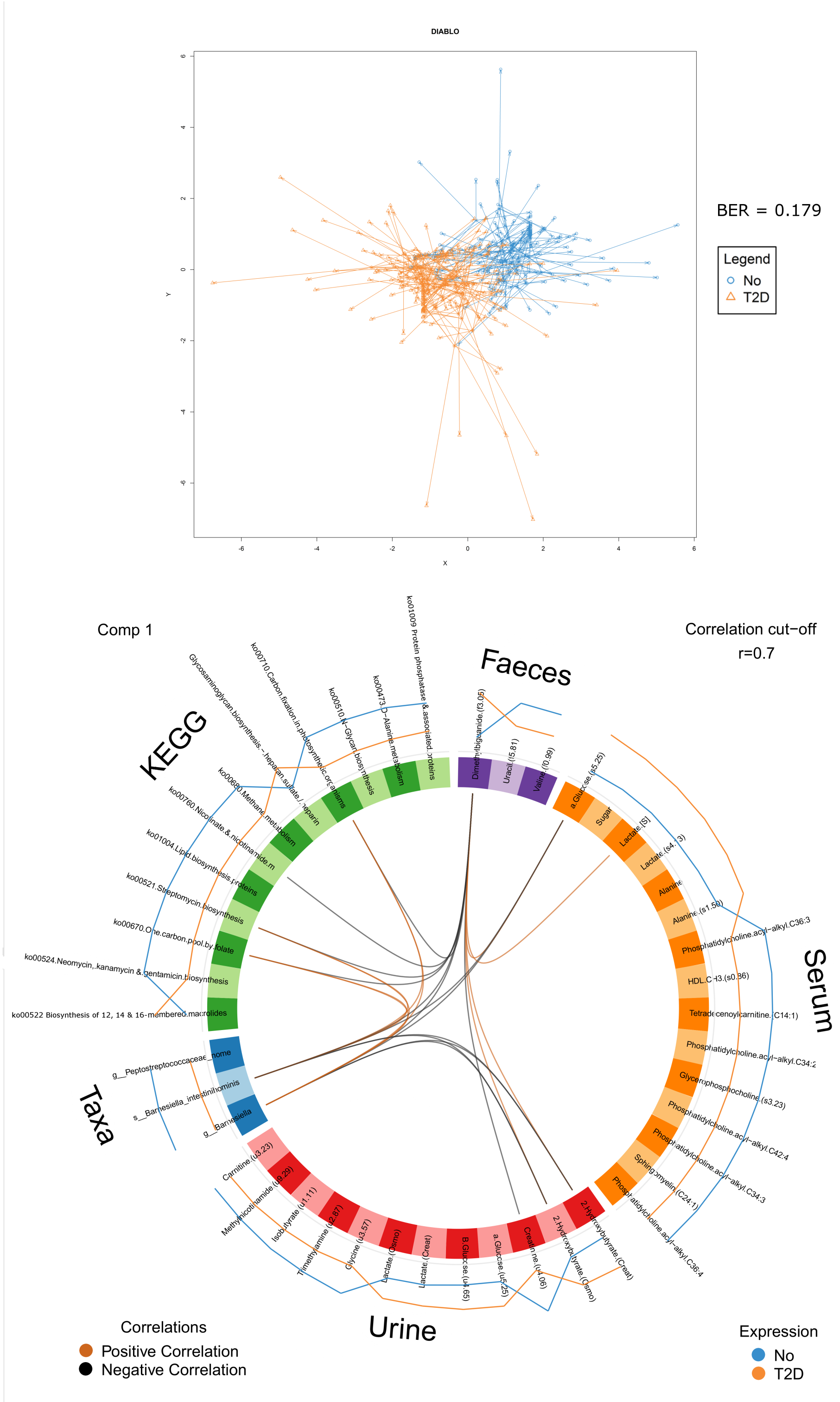

Supplementary Figure 18 – Comparison of integrated metabolite & gut microbiome datasets derived from participants with type 2 diabetes (T2D) versus participants without diabetes using Data Integration Analysis for Biomarker discovery using Latent cOmponents (DIABLO). Top – Arrow scores plots of participants with T2D (n=42) & without diabetes (n=27) projected in latent space. Start of arrows indicate the centroid between all data sets for a given sample, tips of arrows show the location of that sample in each dataset block. Bottom – Variables from component 1 (coloured by dataset) discriminating between T2D and non-diabetic groups are displayed. Differences in expression of variables between groups are shown by the colour-coded outer lines. Correlations (r>0.7) between variables are shown by the colour-coded inner lines. Cross-validated balanced error rate (BER) = 0.179.

Supplementary Figure 19 – DIABLO model comparing changes in integrated datasets after RYGB Vs VSG

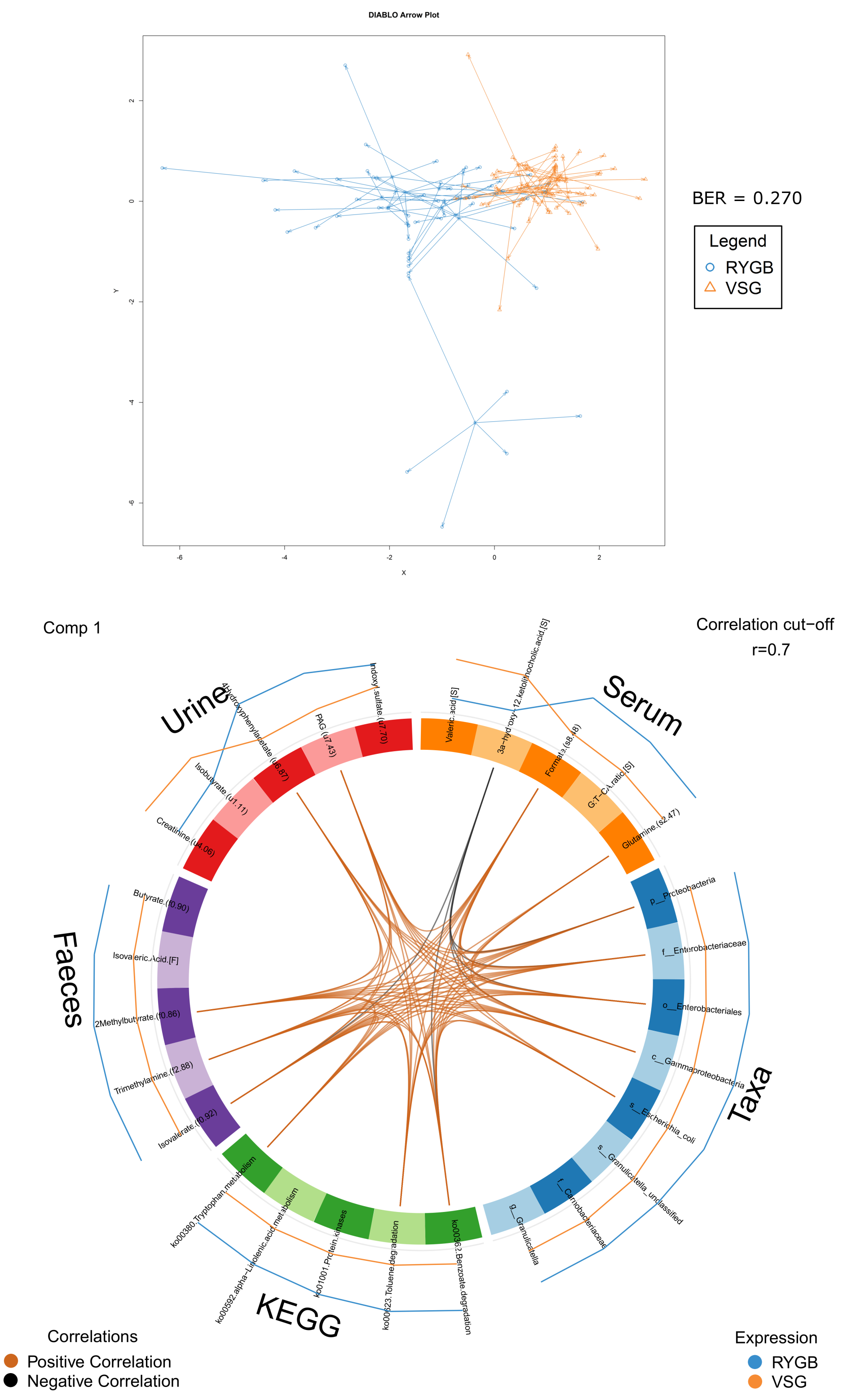

Supplementary Figure 19 – Comparison of changes in integrated metabolite & gut microbiome datasets 3-months after Roux-en-Y Gastric Bypass (RYGB) versus Vertical Sleeve Gastrectomy (VSG) using Data Integration Analysis for Biomarker discovery using Latent cOmponents (DIABLO). Top – Arrow scores plots of RYGB (n=10) & VSG (n=14) samples projected in latent space. Start of arrows indicate the centroid between all data sets for a given sample, tips of arrows show the location of that sample in each dataset block. Bottom – Variables from component 1 (coloured by dataset) discriminating between RYGB and VSG groups are displayed. Differences in expression of variables between groups are shown by the colour-coded outer lines. Correlations (r>0.7) between variables are shown by the colour-coded inner lines. Cross-validated balanced error rate (BER) = 0.270.

Supplementary Figure 20 – Metabolite – Microbiome correlations

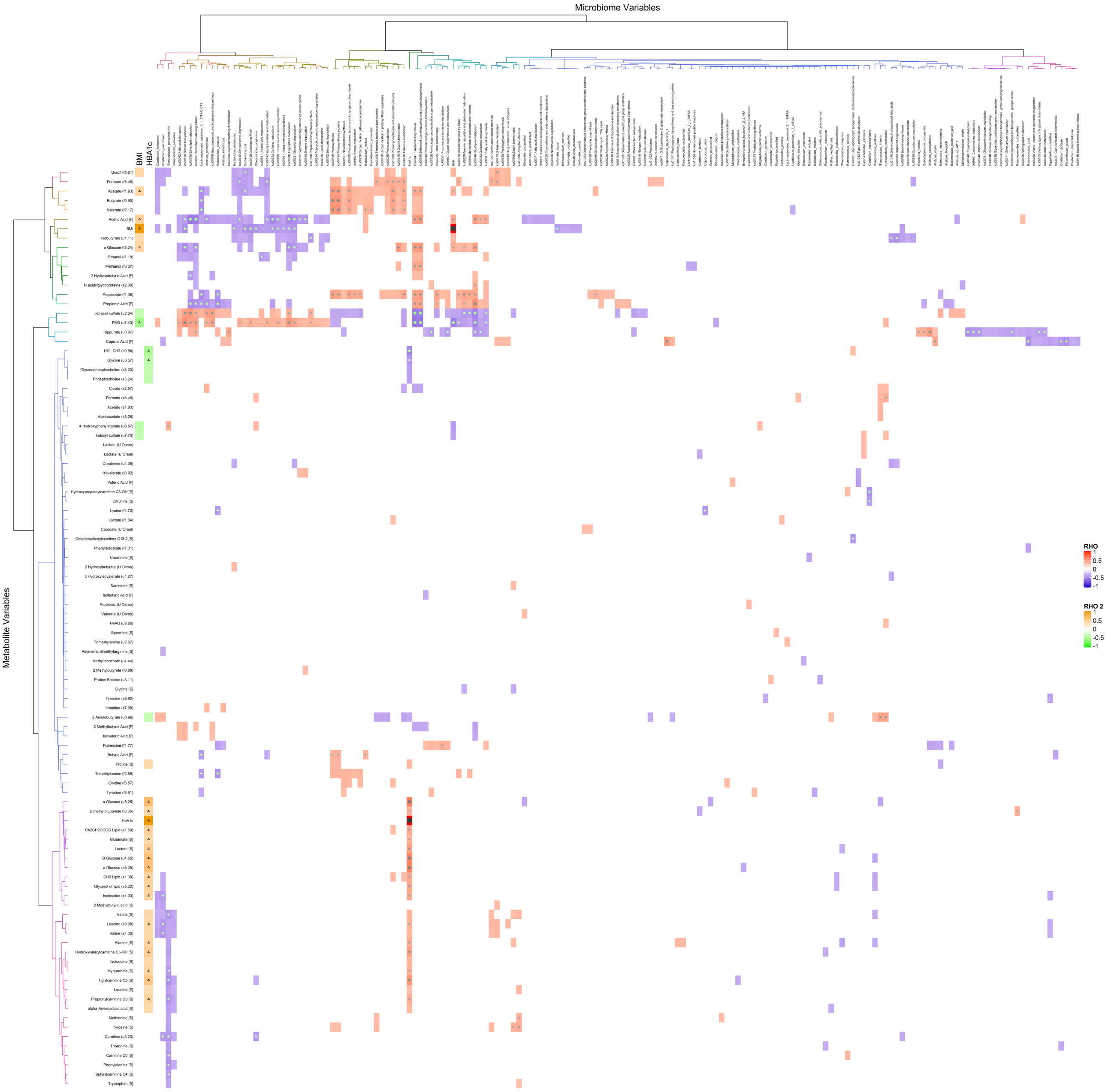

Supplementary Figure 20 – Gut microbial species / KEGG pathways – metabolite interactions (n=108). Significant Spearman's correlations (pFDR <0.05) are shown and shaded according to strength of correlation coefficient (Rho). Highly significant correlations (pFDR<0.01) are highlighted with a circle. Circle size is inversely proportional to the pFDR value. Correlations are clustered according to Euclidean distances. Correlations to BMI and HbA1c are highlighted in the first two columns. Correlations to BMI and HbA1c with a pFDR<0.01 are highlighted with a triangle. Metabolites measured in serum, urine and faeces are marked with [S, U & F] respectively. Bile acid and lipid datasets have been excluded.
