# Supplementary material for: Multi-omic phenotyping reveals host-microbe responses to bariatric surgery, glycaemic control and obesity
Source: Commun Med (Lond). 2022 Oct 7;2:127. doi: 10.1038/s43856-022-00185-6 (PMC9546886; doi:10.1038/s43856-022-00185-6)
Supplement: Supplementary file 11 — Description of Additional Supplementary Files [file 43856_2022_185_MOESM11_ESM.docx]

**Description of Additional Supplementary Files**

**File Name:** Supplementary Data 1

**Description:** Table of identified metabolites associated with Bariatric Surgery, Weight / BMI, T2D and Diet. Metabolites from Serum, Urine and Faecal biofluids with associations (pFDR<0.05) to 1) post- versus pre-bariatric surgery, 2) lower weight / BMI, 3) lower HbA1c / participants without Vs with T2D, 4) lower dietary substrate / higher AHEI score. Metabolites with concordant changes are grouped together. See Figure 8 for individual dietary correlations.

**File Name:** Supplementary Data 2

**Description:** Univariate analysis of semi-quantified (relative concentrations) of measured metabolites from serum, urine and faecal water ^1^H-NMR experiments in participants with type 2 diabetes (T2D), impaired glucose tolerance (IGT) and participants without diabetes.

**File Name:** Supplementary Data 3

**Description:** Measured bile acids in serum and faeces in participants with type 2 diabetes (T2D), impaired glucose tolerance (IGT) and participants without diabetes.

**File Name:** Supplementary Data 4

**Description:** Measured SCFAs and other carboxylic acids in serum, urine and faeces of participants with type 2 diabetes (T2D), impaired glucose tolerance (IGT) and participants without diabetes.

**File Name:** Supplementary Data 5

**Description:** Measured serum acylcarnitines, amino acids, biogenic amines, glycerophospholipids and sphingomyelins in participants with type 2 diabetes (T2D), impaired glucose tolerance (IGT) and participants without diabetes.

**File Name:** Supplementary Data 6

**Description:** Univariate analysis of changes post Roux-en-Y Gastric Bypass (RYGB) and Vertical Sleeve Gastrectomy (VSG) procedures in semi-quantified metabolites (relative concentrations) measured from serum, urine and faecal water ^1^H-NMR experiments.

**File Name:** Supplementary Data 7

**Description:** Change in measured bile acids in serum and faeces in participants post Roux-en- Y Gastric Bypass (RYGB) and Vertical Sleeve Gastrectomy (VSG) procedures.

**File Name:** Supplementary Data 8

**Description:** Change in SCFAs and other carboxylic acids in serum, urine and faeces of participants post Roux-en-Y Gastric Bypass (RYGB) and Vertical Sleeve Gastrectomy (VSG) procedures.

**File Name:** Supplementary Data 9

**Description:** Change in measured serum acylcarnitines, amino acids, biogenic amines, glycerophospholipids and sphingomyelins in participants post Roux-en-Y Gastric Bypass (RYGB) and Vertical Sleeve Gastrectomy (VSG) procedures.
