# Supplementary material for: Multi-omic phenotyping reveals host-microbe responses to bariatric surgery, glycaemic control and obesity
Source: Commun Med (Lond). 2022 Oct 7;2:127. doi: 10.1038/s43856-022-00185-6 (PMC9546886; doi:10.1038/s43856-022-00185-6)
Supplement: Supplementary file 13 — Reporting Summary [file 43856_2022_185_MOESM13_ESM.pdf]

nature research

Corresponding author(s): Nicholas Penney, Elaine Holmes

Last updated by author(s): 30/07/22

Reporting Summary

Nature Research wishes to improve the reproducibility of the work that we publish. This form provides structure for consistency and transparency in reporting. For further information on Nature Research policies, see our Editorial Policies and the Editorial Policy Checklist.

Statistics

For all statistical analyses, confirm that the following items are present in the figure legend, table legend, main text, or Methods section.

n/a	Confirmed	
☐	☒	The exact sample size (n) for each experimental group/condition, given as a discrete number and unit of measurement
☐	☒	A statement on whether measurements were taken from distinct samples or whether the same sample was measured repeatedly
☐	☒	The statistical test(s) used AND whether they are one- or two-sided<br>*Only common tests should be described solely by name; describe more complex techniques in the Methods section.*
☐	☒	A description of all covariates tested
☐	☒	A description of any assumptions or corrections, such as tests of normality and adjustment for multiple comparisons
☐	☒	A full description of the statistical parameters including central tendency (e.g. means) or other basic estimates (e.g. regression coefficient) AND variation (e.g. standard deviation) or associated estimates of uncertainty (e.g. confidence intervals)
☐	☒	For null hypothesis testing, the test statistic (e.g. F, t, r) with confidence intervals, effect sizes, degrees of freedom and P value noted<br>*Give P values as exact values whenever suitable.*
☐	☒	For Bayesian analysis, information on the choice of priors and Markov chain Monte Carlo settings
☐	☒	For hierarchical and complex designs, identification of the appropriate level for tests and full reporting of outcomes
☐	☒	Estimates of effect sizes (e.g. Cohen's d, Pearson's r), indicating how they were calculated

Our web collection on statistics for biologists contains articles on many of the points above.

Software and code

Policy information about availability of computer code

Data collection	Online 24hr dietary recall questionnaire: www.myfood24.org<br>NMR aquisition: TopSpin 3.1<br>LC-MS aquisition: TargetLynx XS (version 4.1)<br>GC-MS aquisition: MassHunter Quantitative Analysis (version B.07.01)

Data analysis

NMR analysis: MATLAB R2014a (Mathworks)
LC-MS quantification: TargetLynx XS (version 4.1)
GC-MS quantification: MassHunter Quantitative Analysis (version B.07.01)
Metagenomic sequencing: SCalable Metagenome Pipeline (SCamP) available at https://github.com/jamesabbott/SCaMP
NMR multivariate statistical analysis: SIMCA 15 (Umetrics)
General statistical analysis e.g. Multiple testing corrections, univariate analysis, correlation analysis: Matlab scripts
DIABLO: mixOmics package in R (version 6.10.8)
RM-MCCV-PLSDA: custom script in Matlab
NMR metabolite identification e.g. STORM, STOCSY analysis: custom scripts in Matlab
Euler diagram: Eulerr package in R (version 6.1.1)
Phylogenetic Trees: GraPhlAn in Python
Correlation network diagram: Cytoscape 3.8.0
Correlation heatmaps: ComplexHeatmap in R (version 2.2.0)

The code to execute RM-MCCV-PLSDA (and also PLS, OSC-PLS, CA-PLS) is provided in https://bitbucket.org/jmp111/capls/src. The code for executing both the STOCSY and STORM algorithms is in https://bitbucket.org/jmp111/storm/src. These codes can be executed in a Matlab environment.

For manuscripts utilizing custom algorithms or software that are central to the research but not yet described in published literature, software must be made available to editors and reviewers. We strongly encourage code deposition in a community repository (e.g. GitHub). See the Nature Research guidelines for submitting code & software for further information.

Data

Policy information about availability of data

All manuscripts must include a data availability statement. This statement should provide the following information, where applicable:

- Accession codes, unique identifiers, or web links for publicly available datasets
- A list of figures that have associated raw data
- A description of any restrictions on data availability

Metagenomic data has been deposited with GenBank, EMBL and DDBJ databases under the BioProject accession number PRJNA473348.
Further metagenomic and metabolic source data have been deposited at Mendeley Data: http://dx.doi.org/10.17632/t76nm3yfzh.3
Further information and requests for resources should be directed to and will be fulfilled by Elaine Holmes (elaine.holmes@imperial.ac.uk).

Field-specific reporting

Please select the one below that is the best fit for your research. If you are not sure, read the appropriate sections before making your selection.

☒ Life sciences ☐ Behavioural & social sciences ☐ Ecological, evolutionary & environmental sciences

For a reference copy of the document with all sections, see nature.com/documents/nr-reporting-summary-flat.pdf

Life sciences study design

All studies must disclose on these points even when the disclosure is negative.

Sample size

Formal sample size calculations for multi-omic datasets are difficult. The sample sizes used were based on prior studies exploring the gut microbiota or metabolome in diabetic cohorts as well as bariatric surgery cohorts. (Few studies have explored combined longitudinal host-microbe interactions in human cohorts following bariatric surgery, with most studies focusing on either the microbiota or the metabolome). The large differences between groups, combined with the use of paired data sets with participants pre- and post-surgery has enabled us to establish high degrees of statistical significance for the detected changes. In addition we have applied multiple testing corrections where relevant to reduce the risk of type-1 errors.

Data exclusions

Recruitment inclusion and exclusion criteria are detailed below. No data that met quality control standards was excluded from the study analysis. A small numbers of samples did not meat quality control criteria after NMR / MS analysis and were excluded from downstream analysis. The number of samples used for each analysis are detailed in the manuscript and / or supplementary results.

Replication

All metabolic and metagenomic anlyses were performed using a number of quality control checks to ensure accurate results. For example all metabolic analyses included pooled quality control samples interspersed after every 10 samples to ensure a low QC sample coefficient of variation.
Study analyses were not replicated.

Randomization

Participants were allocated into diabetic or non-diabetic groups according to their disease state. To control for obesity associated with type-2 diabetes the non-diabetic group were also obese.
Patients were allocated to either the RYGB or VSG procedure according to patient choice on the advice of the multidisciplinary medical team. Although this was not randomised, patient demographics were similar between groups as described in the manuscript. Furthermore, participants were profiled longitudinally (pre- and post-surgery) to allow assessment of the profound intra-individual changes that bariatric surgery induces and reducing the influence of inter-individual differences.
The sample order for metabolic and metagenomic analyses performed in the study were randomised using a random number generator.

Blinding

Participants were not blinded to their disease state (diabetic / non-diabetic) or the surgical procedure that they underwent.

Reporting for specific materials, systems and methods

We require information from authors about some types of materials, experimental systems and methods used in many studies. Here, indicate whether each material, system or method listed is relevant to your study. If you are not sure if a list item applies to your research, read the appropriate section before selecting a response.

Materials & experimental systems

n/a	Involved in the study
☒	☐ Antibodies
☒	☐ Eukaryotic cell lines
☒	☐ Palaeontology and archaeology
☒	☐ Animals and other organisms
☐	☒ Human research participants
☐	☒ Clinical data
☒	☐ Dual use research of concern

Methods

n/a	Involved in the study
☒	☐ ChIP-seq
☒	☐ Flow cytometry
☒	☐ MRI-based neuroimaging

Human research participants

Policy information about studies involving human research participants

Population characteristics	Patients referred for consideration of bariatric surgery who were obese (BMI >30kg/m2), aged ≥18, had failed efforts at lifestyle modification and dieting and were willing to comply with the trial protocol were recruited prospectively. Diabetics (HbA1c >48mmol/mol or treated) and non-diabetics were eligible for recruitment. Patients who had previously undergone bariatric or major abdominal surgery, were or intended to become pregnant during trial period, or took long-term antibiotics were excluded. Major abdominal surgery included patients who had undergone small or large bowel resection, liver, pancreatic, splenic or stomach surgery, as these could influence the gut microbiota and / or the patient's metabolic state. Patients that had previously had an appendicectomy, cholecystectomy or hernia repair were not excluded.
Recruitment	Individuals meeting the criteria for study inclusion were approached for recruitment by the research team at the time of their clinic appointments for work up towards potential bariatric surgery. The study protocol and sample collection instructions were co-developed with patient representatives to help reduce the study burden for patients. To improve patient compliance recruitment and sample collection occurred at the time of patients' usual NHS appointments prior to surgery and at 3-months post-procedure. A small exploratory cohort were also sampled at 1-year post-procedure. As stated above, patients were allocated to either the RYGB or VSG procedure according to patient choice on the advice of the multidisciplinary medical team. Although this was not randomised, patient demographics were similar between groups as described in the manuscript. Furthermore, participants were profiled longitudinally (pre- and post-surgery) to allow assessment of the profound intra-individual changes that bariatric surgery induces and reducing the influence of inter-individual differences.
Ethics oversight	NHS Research Ethics Committee (15/ES/0026)

Note that full information on the approval of the study protocol must also be provided in the manuscript.

Clinical data

Policy information about clinical studies

All manuscripts should comply with the ICMJE guidelines for publication of clinical research and a completed CONSORT checklist must be included with all submissions.

Clinical trial registration	ClinicalTrials: NCT02421055
Study protocol	The study protocol is described in full in the methods section of the manuscript.
Data collection	Participants were recruited over a 20 month period at St Mary's Hospital, Imperial College Healthcare NHS Trust, London. Sample and other data collections (e.g. participant diet and anthropometric data) occurred at baseline before surgery, and 3 months post-surgery. Data / sample collection also occurred 1 year post-surgery in a small cohort of patients.
Outcomes	The primary clinical outcome was weight loss. The secondary clinical outcome was diabetes resolution (HbA1c < 48mmol/mol). However, the primary aim of the study was to to establish changes in the host-microbe interactions in volunteers with obesity +/- T2D and in individuals undergoing bariatric surgery with and without T2D to identify dysregulated metabolic and gut microbial pathways in T2D that are functionally restored after bariatric surgery.
